# Genome-wide association study reveals candidate genes for body size and reproductive traits in Hu sheep

**DOI:** 10.5713/ab.250716

**Published:** 2025-11-10

**Authors:** Mao Li, Xin Xiang, Wei Gao, Liran Zhao, Zhengguang Wang, Kui Li

**Affiliations:** 1 College of Animal Sciences, Zhejiang University, Hangzhou, China; 2 Hainan Institute, Zhejiang University, Sanya, China; 3 Zhejiang Key Laboratory of Nutrition and Breeding for High-Quality Animal Products, Hangzhou, China; 4 Zhejiang Provincial Animal Husbandry Technology Extension and Breeding Livestock and Poultry Monitoring Station, Hangzhou, China

**Keywords:** Body Size, Genome-wide Association Study, Hu Sheep, Reproductive Performance, Sheep Breeding

## Abstract

**Objective:**

Larger body size and enhanced reproductive performance are correlated with increased profitability for sheep farmers. Hu sheep have smaller statures compared to other meat sheep breeds, necessitating improvement. The molecular mechanisms underlying the high fecundity of Hu sheep remain unclear. Body size and reproductive traits are economically important traits in Hu sheep production, necessitating further research.

**Methods:**

We conducted direct measurements of the body size traits, including body weight, body height and length, chest circumference and cannon bone circumference, for 558 Hu sheep. Additionally, we statistically recorded their reproductive traits, specifically litter size and teat number. Candidate genes for these traits were identified using a mixed linear model in a genome-wide association study (GWAS). The significant threshold for single nucleotide polymorphism was determined using a 1,000 permutation test. Subsequently, functional annotations were conducted on the candidate genes.

**Results:**

We identified *CHST3* as a key candidate gene affecting the body size, along with *SCMH1* and *BAZ2B* as key candidate genes influencing reproductive traits in Hu sheep. *CHST3* affected multiple body size traits and was highly expressed in the muscle tissues of Hu sheep. The *SCMH1* and *BAZ2B* were significantly annotated by GWAS and selection signature, and they were highly expressed in the reproductive system of Hu sheep. Furthermore, through a phenome-wide association study in humans, we found that these key candidate genes were significantly associated with similar traits in human population.

**Conclusion:**

This represents the initial evidence linking these genes to body size and fecundity in Hu sheep. These findings provide genetic markers for selective breeding, and contribute to the selection process for high-quality breeding sheep.

## INTRODUCTION

Body size and reproductive performance are important traits in sheep. Economically, body size directly correlates with meat yield, while reproductive performance dictates production efficiency. Biologically, these traits have been subject to natural and artificial selection over long periods, representing the direction of adaptation and evolution. Therefore, understanding the genetic architecture of these traits is crucial for selective breeding and elucidating the underlying genetic mechanisms. Previous studies have primarily focused on single trait and have not performed functional annotations for candidate genes. Hu sheep is an indigenous breed in China, characterized by rapid growth and high fertility. However, Hu sheep is small in stature compared to other meat sheep breeds; and the molecular mechanisms underlying high fertility remain unclear.

Sheep body size and reproductive performance can be influenced by various factors, especially genetics [1]. Currently, most studies on the body size of sheep only focus on body weight, while neglecting other body size traits. Specifically, the cannon bone circumference (CBC) is an important body size trait in sheep. Increasing the CBC of sheep can improve the weight-bearing capacity of legs and gain larger body size [2]. The study of CBC will be beneficial in proposing new breeding strategies to improve the body size of sheep. Additionally, litter size (LS) is important reproductive trait that affect the production yield of sheep [3]. Teat number is important reproductive trait that directly affects the lactation rate of ewes and the survival rate of lambs [4]. Despite the importance of LS and teat number, the underlying genetic mechanisms have not been elucidated, which has hindered the effective advancement of sheep breeding.

Previous studies on economic traits in sheep use a single analytical approach to screen for relevant loci. The differences between genotypes at specific loci and the expression of candidate genes in tissues and systems have not been focused on. More recently, phenome-wide association study (PheWAS), which is a complementary approach to GWAS, has been used to associate certain genetic variants with many phenotypes to study their pleiotropy and causality among big data [5]. Orthologous genes often show similar functions across species. The use of rich GWAS data from humans to conduct a PheWAS might contribute to improve the characterization of the pleiotropic effects of candidate genes and elucidate the genetic architecture of complex traits in the target species [6]. Therefore, it is critical to use more comprehensive analytical approaches to identify relevant loci and candidate genes, and explore the underlying molecular mechanisms.

Hu sheep are renowned worldwide for prolificacy. It has the advantages of early sexual maturity, high fertility, high carcass yield, tender meat, and low fatty content. In this study, we sequenced 558 Hu sheep, and estimated the heritability of each trait as well as the correlations between body size traits. The related loci and candidate genes were screened using GWAS, PheWAS, selection signatures (Fst, ROH, ROHet, integrated haplotype score [IHS]) and functional region annotation. Furthermore, we had analysed the expression level of the candidate genes in the tissues and systems of Hu sheep and explored the potential molecular mechanisms. This study aimed to identify candidate genes influencing body size and reproductive traits in Hu sheep using integrative genomic approaches. These genes will provide valuable genetic markers for selective breeding.

## MATERIALS AND METHODS

### Management and phenotypic measurement

All Hu sheep (n = 558) used in this study were obtained from the Yihui Ecological Agriculture. All Hu sheep were raised under consistent feeding environment and nutritional conditions with the same commercial diets and unrestricted access to water. Each phenotype was measured by the same person to reduce measurement error. Body weight of Hu sheep was measured using an electronic scale. Body height (BH) is the vertical distance from the highest point of the withers to the ground. Body length (BL) is the straight-line distance from the anterior margin of the shoulder to the posterior margin of the rump. Chest circumference is the circumference of the torso at the posterior margin of the scapulae. Cannon bone circumference is the circumference at the one-third point of the left forelimb. The aforementioned body size traits were measured using a professional measuring stick and a measuring tape. LS and teat number were recorded. LS refers to the number of lambs born to a ewe in a single reproductive cycle. The sex, birth date, age at months, age at lambing (in months), and lambing date could be found in the supplementary materials (Phenotypic data).

### DNA extraction methods

Blood samples were collected from the posterior veins of Hu sheep and stored at −80°C until DNA extraction. The CWE9600 magnetic bead Blood DNA Kit and the magnetic bead method were used for DNA extraction. The extracted DNA samples were subjected to DNA sample integrity testing using a 1.5% agarose gel, followed by DNA purity testing using the NanoDrop 2000 Nucleic Acid Protein Assay Instrument and accurate quantification using the dsDNA HS Assay Kit for Qubit.

### Library construction and genotyping

The Universal Library Construction Kit (MGI) NadPrep DNA was used for library construction. More than 1 μg of genomic DNA was randomly fragmented into pieces of approximately 300–350 bp using the Covaris crusher. Following end repair, addition of A-tail, and ligation of sequencing junctions, DNA fragments of approximately 300–350 bp were screened using NadPrep SP Beads. The polymerase chain reaction (PCR) amplification was then performed, and the resulting PCR products were purified using NadPrep SP Beads to produce a sequencing library. After library construction was completed, preliminary quantification was performed using Qubit 2.0 and the library was tested for inserts using a Bioanalyzer (Agilent).

The resequencing data were filtered and indexed based on the sheep reference genome ARS-UI_Lamb_v2.0. The BWA software was used to compare clean reads with the reference genome, and samtools was used to sort and build indexes. GATK was used to deduplicate the Bam files, and calculated the sequencing depth and genome coverage for each sample based on the Bam files, which were prepared for subsequent mutation detection. Using the VariatFilter module to strictly filter SNPs. The following SNP loci would be excluded: a minimum allele frequency of <0.05, a Hardy-Weinberg test p-value of <0.000001, and typing deletions >10%.

The chip data was genotyped using the sheep 50K chips. The chip was manufactured and designed by Beijing Compass Biotechnology. SNPs with a call rate lower than 90%, and a minor allele frequency lower than 5% were filtered out using the Plink (ver. 1.90) software [7]. The overlapping SNPs shared by both datasets was extracted and served as the basis for downstream analyses.

### Heritability and correlations calculation

The following model was used to calculate the heritability and correlations between traits:


(1) Y=Xb+Za+e

In the above equation, where *y* is the trait vector; *b* is the fixed effects (body size traits include sex, measurement year and season, and measurement age of Hu sheep; reproductive traits include year and season of lambing, age of ewes); *a* is a random effect and obeys normal distribution with mean 0 and variance $G\sigma_{a}^{2}$, which can be noted as, $a\sim N(0,G\sigma_{a}^{2}),\sigma_{a}^{2}$ is the hereditary variance; *X* and *Z* are the correlation matrices of *b* and *a*, respectively; *e* is the residual effect, obeying a normal distribution $N(0,I\sigma_{a}^{2}),\sigma_{a}^{2}$ is the residual variance; *G* is the kinship matrix between individuals. The formula for calculating heritability is: $h^{2}=\frac{\sigma_{a}^{2}}{\sigma_{a}^{2}+\sigma_{e}^{2}}$. Genetic and phenotypic correlations between traits were calculated using HIBLUP software [8]. The genetic (*r_g_*) and phenotypic correlations (*r_p_*) between traits were estimated using two-trait model for pair-wise traits. The form of the model remains as shown in equation (1). Where *a* and *e* are assumed to follow normal distributions *N* (0, *G*⊗*H*) and *N* (0, *I*⊗*R*), respectively. *H* is the additive genetic (co)variance matrix between each pair of traits, and *R* is the residual (co)variance matrix between traits; ⊗is the Kronecker product of matrices. The equations for calculating *r_g_* and *r_p_* are as follows [9]:


(2) $$r_{g}=\frac{\sigma_{a_{1}a_{2}}}{\sqrt{\sigma_{a_{1}}^{2}\sigma_{a_{2}}^{2}}};r_{p}=\frac{\sigma_{a_{1}a_{2}}+\sigma_{e_{1}e_{2}}}{\sqrt{\left(\sigma_{a_{1}}^{2}+\sigma_{e_{1}}^{2}\right)\left(\sigma_{a_{2}}^{2}+\sigma_{e_{2}}^{2}\right)}}$$

Where *σ_a_*_1_*_a_*_2_ and *σ_e_*_1_*_e_*_2_ are the additive genetic covariance and the residual covariance between each pair of traits; $\sigma_{a1}^{2},\sigma_{a2}^{2}$ and $\sigma_{e1}^{2},\sigma_{e2}^{2}$ are the additive genetic variance and the residual variance of each trait.

### Genome-wide association study

The GWAS was conducted using the rMVP software [10]. The GWAS analysis uses a mixed linear model that accurately accounts for population structure and complex kinship relationships within the population [11]. The formula for this model is as follows:


(3) $$y=X\beta +Z_{k}\gamma_{k}+\xi +e$$

Where *y* is the phenotype vector, *Xβ* is the fixed effects, including population structure, sex, measurement year and season, and measurement age of Hu sheep, *Z_k_γ_k_* is the marker effect to be tested, ξ~*N* (0, *Kφ*^2^) represents the polygenic effect, and *e*~*N* (0, *Iσ*^2^) is the residual effect. *K* is the polygenic effect in the marker-inferred kinship matrix. The qqman package in R software was used, to plot the Manhattan and Q-Q plots [12]. The significant SNP threshold was determined using a 1,000 permutation test.

### Gene annotation and QTL enrichment

The reference genome of Ovis aries (assembly ARS-UI_Ramb_v2.0) was downloaded from the ENSEMBL website, and significant SNPs were annotated to their corresponding genes using the ANNOVAR software [13]. Additionally, the Animal QTL Database (http://www.animalgenome.org/QTLdb) was used to annotate potential functions of the selected regions. The QTL annotation was performed using the GALLO package of the R software [14].

### Functional region annotation and expression level analysis

The significant SNP loci were annotated to the reference genome Oar_v4.0 using LiftOver software [15]. Subsequently, functional annotations of the significant SNPs were performed in the sheep enhancer database (http://genome.ucsc.edu/s/zhypan/oviAri4_chr_state). The significant SNPs were annotated for alternative polyadenylation (APA) in the Animal-APA Database [16]. The eRNA in genes were annotated in the Animal-eRNA database [17]. The expression level of candidate genes in tissues and systems of Hu sheep were queried in the Herbivore Transcriptome Information Resource Database, and KEGG pathways enrichment analysis was conducted for the candidate genes [18]. To explore whether the orthologues of candidate genes detected for body size traits in sheep were associated with complex traits in humans, we conducted a PheWAS for these genes based on the human GWAS data in the GWASATLAS database [19].

### Selection signature analysis

We conducted ROH detection to identify regions of genetic homozygosity in Plink version 1.90 using a sliding window method with the following parameters: (1) the minimum length of the ROH was 1,000 kb, (2) each ROH had at least one SNP per 150 kb, (3) a sliding window of 25 SNPs and one heterozygous genotype was allowed in a window, and (4) each ROH contained at least 25 consecutive SNPs. The percentage of SNP occurrences in ROHs was calculated to characterize the genomic regions of ROH hotspots, and the threshold of ROH hotspots was set as the top 0.1% of SNP occurrences. Population differentiation was assessed by calculating Fst values using VCFtools software [20], the threshold was set as the top 0.1% of Fst values. The number of individuals in the high-performance group for LS is 199, while the number in the low-performance group is 229. The number of individuals in the high-performance group for teat number is 131, while the number in the low-performance group is 390. Runs of heterozygosity (ROHet) were identified and analyzed using the detectRUNS R package [21]. Additionally, IHS analysis was conducted using selscan software to detect positive selections [22]. For each analysis, the threshold was set as the top 0.1% of SNPs values.

## RESULTS

### Phenotypic statistics and its heritability calculation

In this study, 558 Hu sheep (36 rams and 522 ewes) were randomly selected, and their body size and reproductive traits were recorded. Body size traits included body weight, BH, BL, chest circumference (CC), and CBC. Reproductive traits included LS, teat number (TN). These traits were statistically analyzed to provide a preliminary overall characteristic. Additionally, the heritability of all traits was estimated using the GBLUP model, ranging from 2.22E-03 to 0.76. (Table 1).

### Genetic and phenotypic correlations between body size traits

As shown in Table 2, in the body size traits, CBC showed high genetic correlations with BW, BH, and CC. Simultaneously, CBC also displayed high phenotypic correlations with BW, BH, and CC. The genetic and phenotypic correlations between CBC and BL were both low. The genetic and phenotypic correlations between BW and BH were both higher than the correlations between BW and BL. Furthermore, the genetic and phenotypic correlations between BH and BL were both negative.

### Genome-wide association study of body size traits in Hu sheep

**Figure 1:**
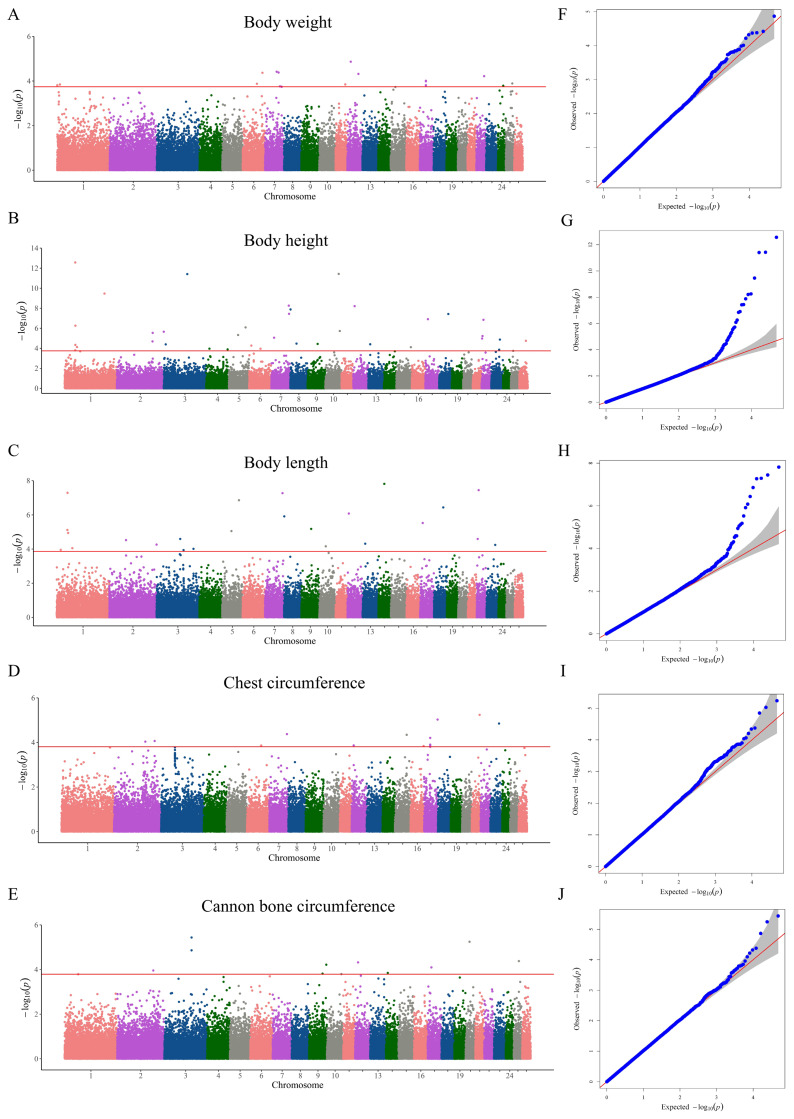
The GWAS of body size traits in Hu sheep. (A–E) Manhattan plots for genome-wide association study of body size traits. (F–J) Quantile-quantile (Q-Q) plots of genome-wide association study for body size traits.

After quality control, 48 493 SNPs were obtained. A total of 103 significant SNPs were identified in the body size traits of Hu sheep (Figure 1A–1E and Supplement 1). The Q-Q plots demonstrated the significant association of certain loci with these traits (Figure 1F–1J). The highest number of significant SNPs was identified in the BH trait, amounting to 37. The most significant SNP among all traits, SNP1_56344040 (p = 2.69E-13), was also located in the BH trait. SNP1_56344040 was annotated to the *ADGRL4* (distance = 872,910 bp), which can be considered as a candidate gene for the BH trait. In the BW and CC traits, there were three identical significant SNPs (SNP17_32588634, SNP17_32587075, and SNP17_32585911), and they were all annotated to the *FAT4*. Therefore, *FAT4* could be a candidate gene for the BW and CC traits. Significant SNP 23_46304583 was found in BH, BL and CC traits. SNP23_46304583 was annotated to the *ARK2N*, and it could be a candidate gene for the BH, BL, and CC traits. The QTL annotation was performed for all significant SNPs, revealing that most significant SNPs were enriched in meat and carcass QTL, followed by production QTL and milk QTL (Supplement 2). In the production QTL, the majority were BW QTL (Supplement 3). The KEGG enrichment analysis was performed for the *ADGRL4*, *FAT4*, and *ARK2N*. The *ADGRL4* was enriched in the G protein-coupled receptors pathway. The *FAT4* was enriched in the Hippo signaling pathway-multiple species and Cell adhesion molecules pathways (Supplement 4). The *ARK2N* was not enriched in KEGG pathway.

### SNP25_27304876 was a key locus affecting cannon bone circumference trait

**Figure 2:**
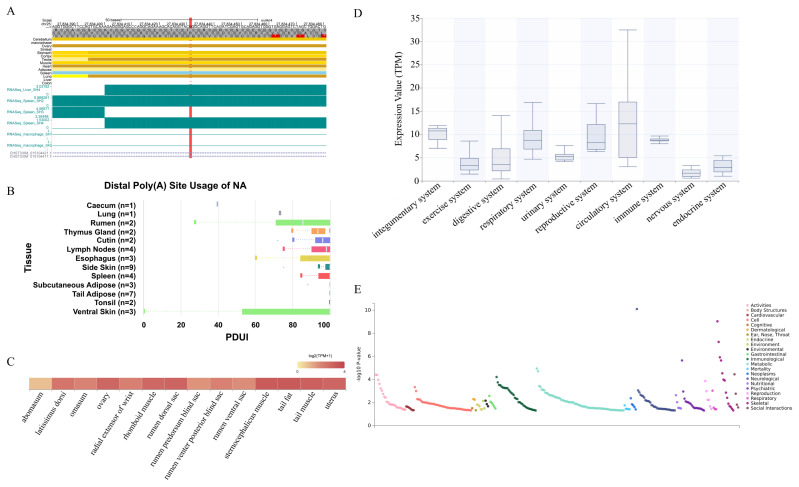
Functional annotation of key locus and expression level of key candidate gene in cannon bone circumference trait. (A) SNP25_27304876 (ARS-UI_Ramb_v2.0) converted to SNP25_27834432 (Oar_v4.0). Annotation map for sheep enhancer region of SNP25_27834432, the red line is the locus of SNP25_27834432. (B) Annotation map for alternative polyadenylation (APA) events of SNP25_27304876. Percentage of distal poly(A) site usage index (PDUI) are used for quantifying APA events. (C) The expression level of *CHST3* in multiple tissues of Hu sheep. The deeper the color, the higher the expression. (D) The expression level of *CHST3* in multiple systems of Hu sheep. (E) Manhattan plot of *CHST3* based on the results of the phenome-wide association study (PheWAS).

In the CBC trait, 11 significant SNPs were identified. Enhancer annotations were conducted for these significant SNPs. It was found that SNP25_27304876 is annotated in enhancer regions of various tissues, including ovary, stomach, testis, muscle, and lung (Figure 2A). Additionally, through the Animal-APA Database, it was discovered that SNP25_27304876 is annotated in APA sites of multiple tissues, including rumen, spleen, and esophagus (Figure 2B). SNP25_27304876 was annotated to the *CHST3*. The *CHST3* exhibited higher expression level in the tail fat (3.26), sternocephalicus muscle (3.23), and tail muscle (3.09) tissues, with moderate expression level in the rumen dorsal sac (2.99), uterus (2.92), ovary (2.87), rhomboid muscle (2.79), and radial extensor of wrist (2.09) tissues of Hu sheep (Figure 2C and Supplement 5). Furthermore, *CHST3* showed higher expression level in the circulatory and integumentary systems, and moderate expression level in the respiratory, immune, and reproductive systems of Hu sheep (Figure 2D and Supplement 5). The *CHST3* was enriched in the glycosyltransferases and glycosaminoglycan biosynthesis-chondroitin sulfate/dermatan sulfate pathways. Through the GWASATLAS database, it was found that *CHST3* is significantly associated with skeletal, immunological, metabolic and activities traits in humans, which affected immunity, metabolism and activity performances in humans (Figure 2E).

### The CHST3 was a key candidate gene affecting body size traits in Hu sheep

**Figure 3:**
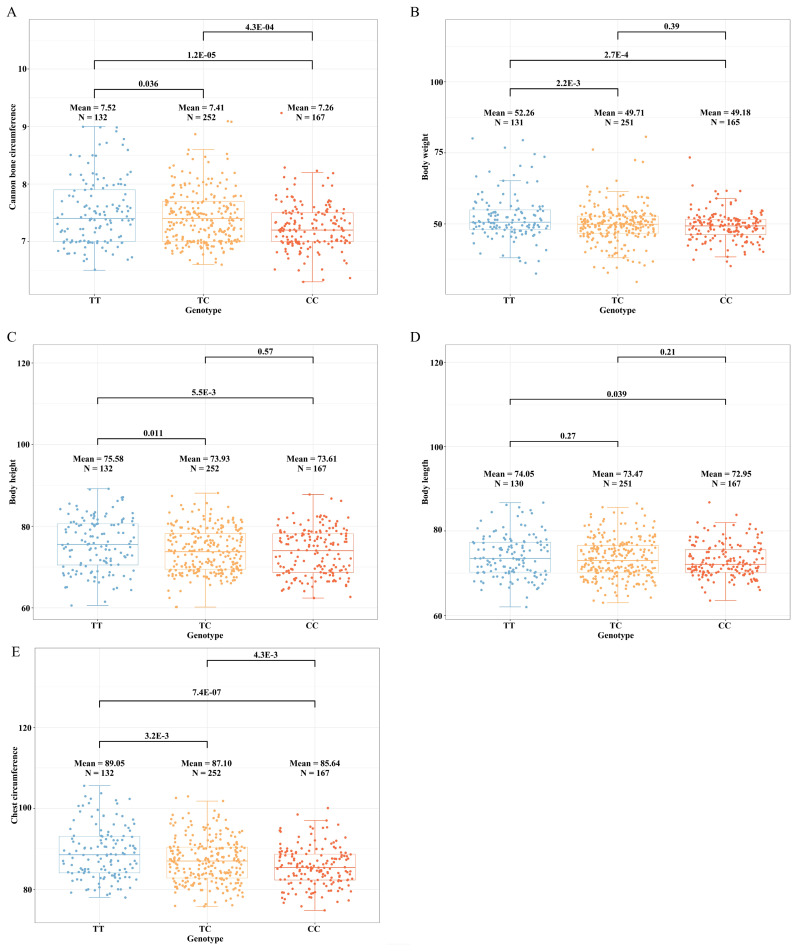
Box plots showing the phenotypic differences between genotypes at the SNP25_27304876 locus. (A) Cannon bone circumference. (B) Body weight. (C) Body height. (D) Body length. (E) Chest circumference.

In the CBC trait, there were three genotypes at the SNP25_ 27304876 locus: TT, TC, and CC. There was a significant difference in CBC between TT and TC genotypes, and also between TT and CC genotypes. In addition, the TT genotype had the largest CBC, while the CC genotype had the smallest (Figure 3A). Therefore, SNP25_27304876 could be a key locus, and *CHST3* could be a key candidate gene for CBC trait. At the SNP25_27304876 locus, the same situation existed in the BW (Figure 3B), BH (Figure 3C), BL (Figure 3D) and CC traits (Figure 3E). In the BL trait, there was no significant difference between TT and TC genotypes, but there was a significant difference between TT and CC genotypes. Furthermore, the TT genotype had the longest BL, and the CC genotype had the shortest (Figure 3D). So, SNP25_27304876 could be a key locus for body size traits, and *CHST3* could be a key candidate gene for body size traits.

### The SCMH1 was a key candidate gene affecting litter size trait in Hu sheep

**Figure 4:**
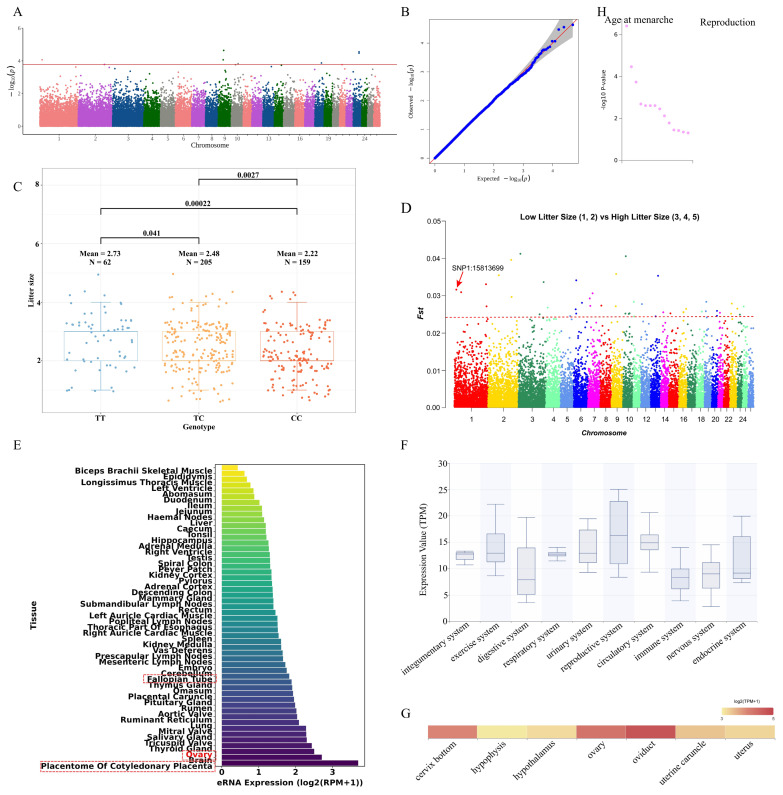
Functional annotation of key locus and expression level of key candidate gene in litter size trait. (A) Manhattan plot for genome-wide association study of litter size trait. (B) Quantile-quantile (Q-Q) plot of genome-wide association study for litter size trait. (C) Box plot showing the litter size difference between genotypes at the SNP 1_15813699 locus. (D) Manhattan plot for fixation indices (Fst) of litter size trait. (E) The expression level of eRNA in *SCMH1* in sheep tissues. (F) The expression level of *SCMH1* in multiple systems of Hu sheep. (G) The expression level of *SCMH1* in multiple tissues of Hu sheep. (H) The *SCMH1* was annotated in the human phenome-wide association study (PheWAS) database, where it is associated with reproductive traits, such as age at menarche.

The genetic correlation between LS and teat number traits was 0.17, and the phenotypic correlation was 0.12. A total of eight significant SNPs were identified in LS trait (Figure 4A, 4B and Supplement 1). There were three genotypes at the significant SNP 1_15813699 locus: TT, TC and CC. The TT genotype had the highest mean LS (2.73) and the CC genotype had the lowest mean LS (2.22) (Figure 4C). There was significant difference between every two genotypes. SNP 1_15813699 was also positively selected in the Fst analysis (Figure 4D). Therefore, SNP 1_15813699 could be a candidate locus for the LS trait. The SNP 1_15813699 was annotated in the *SCMH1*. The *SCMH1* was annotated in the Animal-eRNA database and found that the eRNA in *SCMH1* was highly expressed in the ovary, fallopian tube and placentome of cotyledonary placenta of sheep (Figure 4E). Gene expression level annotation of *SCMH1* in Herbivore Transcriptome Information Resource database revealed that *SCMH1* was expressed at the highest level in the reproductive system of Hu sheep, followed by circulatory system, exercise system and urinary system (Figure 4F and Supplement 3). In the reproductive system, *SCMH1* was expressed at high level in the ovary, oviduct and cervix bottom. In addition, it was expressed at moderate level in the uterus (Figure 4G and Supplement 3). Through the human GWASATLAS database, it was found that *SCMH1* was also associated with reproductive traits in humans (Figure 4H). Among them, the most significantly associated trait was age at menarche.

### The BAZ2B was a key candidate gene affecting teat number trait in Hu sheep

**Figure 5:**
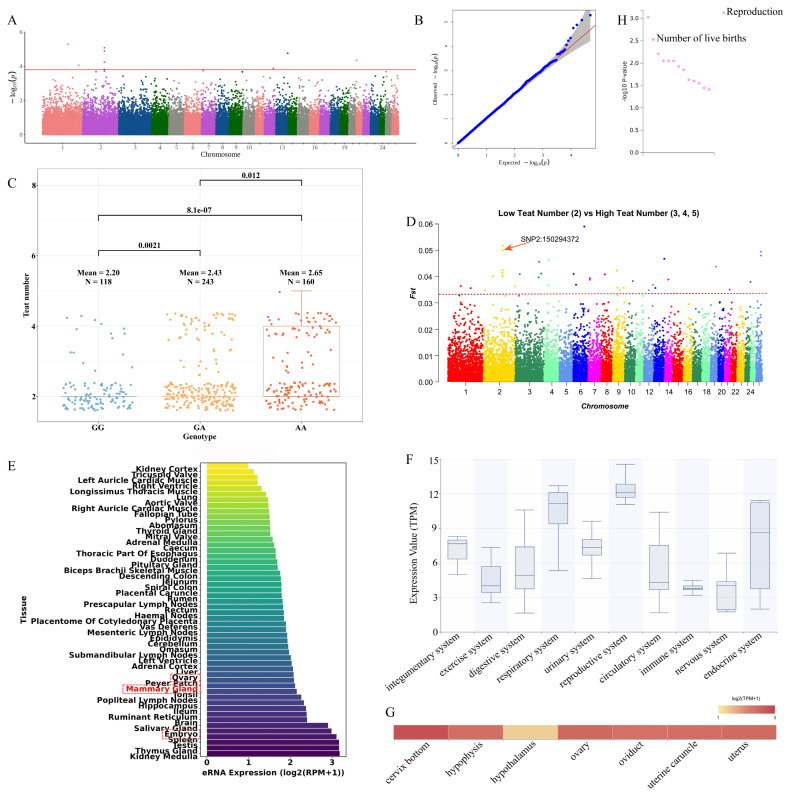
Functional annotation of key locus and expression level of key candidate gene in teat number trait. (A) Manhattan plot for genome-wide association study of teat number trait. (B) Quantile-quantile (Q-Q) plot of genome-wide association study for teat number trait. (C) Box plot showing the teat number difference between genotypes at the SNP 2_150294372 locus. (D) Manhattan plot for fixation indices (Fst) of teat number trait. (E) The expression level of eRNA in *BAZ2B* in sheep tissues. (F) The expression level of *BAZ2B* in multiple systems of Hu sheep. (G) The expression level of *BAZ2B* in multiple tissues of Hu sheep. (H) The *BAZ2B* was annotated in the human phenome-wide association study (PheWAS) database, where it is associated with reproductive traits, such as number of live births.

A total of nine significant SNPs were identified in the TN trait (Figure 5A, 5B and Supplement 1). There were three genotypes at the significant SNP 2_150294372 locus: GG, GA and AA. The AA genotype had the highest mean TN (2.65) and the GG genotype had the lowest mean TN (2.20) (Figure 5C). There was significant difference between every two genotypes. SNP 2_150294372 was also positively selected in the Fst analysis (Figure 5D). Therefore, SNP 2_150294372 could be used as a candidate locus for the TN trait. The SNP 2_150294372 was annotated in *BAZ2B*. The *BAZ2B* was annotated in the Animal-eRNA database and found that the eRNA in *BAZ2B* was highly expressed in mammary gland, ovary and embryo of sheep (Figure 5E). Gene expression level annotation of *BAZ2B* in Herbivore Transcriptome Information Resource database revealed that *BAZ2B* had the highest expression level in the reproductive system of Hu sheep, followed by respiratory system, endocrine system and integumentary system (Figure 5F and Supplement 3). The *BAZ2B* has high expression level in all tissues of the reproductive system except hypothalamus (Figure 5G and Supplement 3). Through the human GWASATLAS database, it was found that *BAZ2B* was also associated with reproductive traits in humans (Figure 5H). Among them, number of live births trait was of attention.

### Selection signature hotspots detection

**Figure 6:**
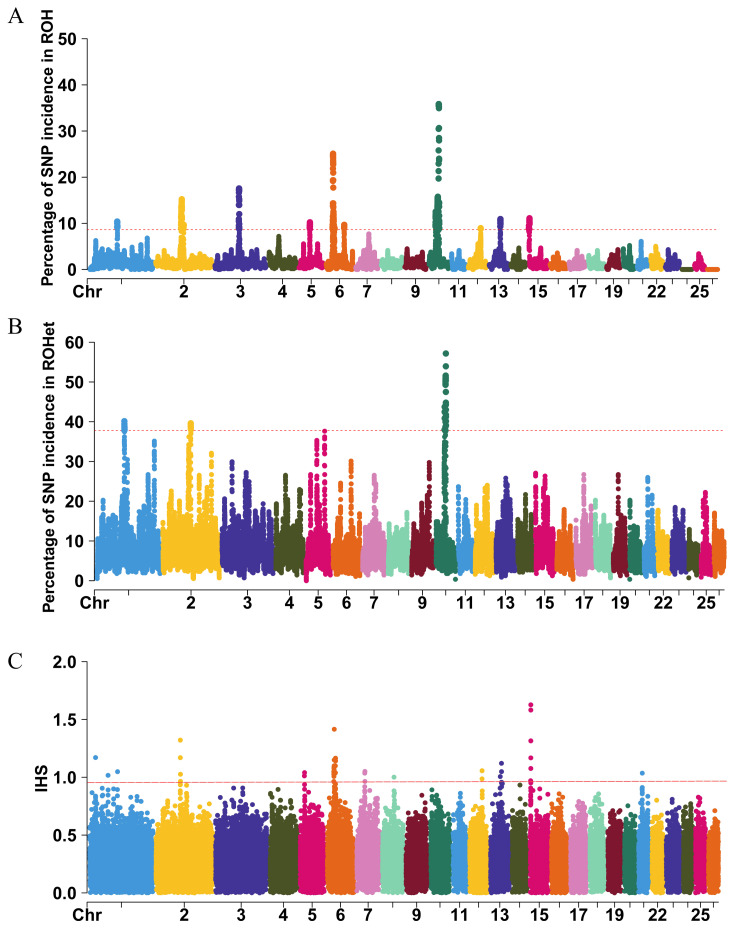
Manhattan plot of selection signatures. (A) Manhattan plot of the occurrence (%) of each SNP in the ROH. (B) Manhattan plot of the occurrence (%) of each SNP in the ROHet. (C) Manhattan plot of the IHS values of each SNP. IHS, integrated haplotype score.

The assessment of genome-wide ROH was conducted on autosomes in the Hu sheep population, resulting in the identification of 8,406 ROH (Figure 6A), and 35 genes overlapped with the ROH hotspots (Supplement 6). Additionally, 9 genes overlapped with the ROHet hotspots (Figure 6B), 19 genes overlapped with the IHS hotspots (Figure 6C, Supplement 6). Four genes were annotated in ROH and ROHet, they were *ATP12A*, *PCDH9*, *OLFML2B*, *TUBGCP5*. Four genes were annotated in ROH and IHS, they were *SLC23A2*, *TSPAN5*, *STPG2*, *GRID2*. No shared candidate genes were identified between the GWAS and selection signature (ROH, ROHet and IHS) hotspots. Through literatures reviewed, it was shown that *GRID2* gene is associated with growth and development of sheep. The *STPG2* gene was associated with reproductive performance of sheep.

## DISCUSSION

This study estimated the heritability of body size and reproductive traits. Moreover, the genetic and phenotypic correlations between body size traits revealed that CBC had an important effect on body size. In particular, three key candidate genes were identified using GWAS, QTL, PheWAS, and functional region annotation. We revealed, for the first time, *CHST3* is a key candidate gene affecting body size traits, *SCMH1* and *BAZ2B* are key candidate genes affecting reproductive traits in Hu sheep. The findings expand our understanding of the genetic basis of economic traits in Hu sheep.

Body size is an important economic trait in sheep production, which may be strongly influenced by genetics [23]. In our study, the heritability of all body size traits was greater than 0.15. This indicates that these traits have moderate to high heritability, and can be effectively improved in future breeding. The highest heritability was found in BH trait at 0.76. This suggests that selecting sheep with higher BH during breeding, their offspring will have a high probability of possessing a higher BH. Furthermore, this study calculated the heritability of CBC trait in Hu sheep for the first time, which was 0.25, lower than that found in pigs [2]. It may be due to species differences. Among the body size traits, CBC had high genetic and phenotypic correlations with BW, BH and CC. It is reasonable to assume that selection for CBC could also significantly alter the BW, BH, and CC. The genetic and phenotypic correlations between BH and BW were higher than those between BL and BW. It can be inferred that BH has a greater influence on BW than BL. Additionally, the genetic and phenotypic correlations between BH and BL were both negative, implying that only one of these traits should be selected for breeding in the Hu sheep population. During the growth and development of sheep, the body’s resources are limited. Investing more resources in BH trait would result in fewer resources being allocated to BL trait. This resource allocation conflict could lead to negative correlations between the two traits. In the body size traits, three candidate genes were identified: *ADGRL4*, *FAT4*, and *ARK2N*. The *ADGRL4* encodes a key regulator of angiogenesis, and the silence of *ADGRL4* affects the Notch pathway, subsequently leading to influences the development of muscle in sheep [24,25]. The *FAT4* could mediate mesenchymal cell clustering and villus formation in gut development [26]. Through the non-canonical Wnt signaling pathway, *FAT4* plays a key role in tissue homeostasis and angiogenesis [27]. Additionally, *FAT4* was enriched in the Cell adhesion molecules pathway, which mediates the development and regeneration of skeletal muscle [28]. It is reasonable to assume that *FAT4* may affect energy metabolism and muscle tissue development in sheep. The *ARK2N* was associated with skeletal muscle function, *ARK2N* deficiency would lead to skeletal abnormalities and reduced body weight in mice [29].

Enhancers are distal DNA elements that regulate gene expression [30]. APA can enhance the regulatory potential of genes, and facilitate tissue-specific genes expression [31]. The *CHST3* affects the proliferation of bone marrow cells, and mutations in this gene will lead to skeletal and cartilage dysplasia [32,33]. In this study, we revealed, for the first time, *CHST3* is a key candidate gene affecting the body size in Hu sheep. SNP 25_27304876 was annotated in enhancers (ovary, stomach, testis, muscle, and lung) and APA regions (rumen, spleen, and esophagus). And SNP25_27304876 was annotated to *CHST3*. Therefore, we speculated that *CHST3* may affect the reproductive, digestive, and immune performances of Hu sheep. Subsequently, *CHST3* was assessed at the gene expression level. We found that *CHST3* had high expression level in tissues such as rumen, ovary, and muscle of Hu sheep. Additionally, *CHST3* was also highly expressed in systems such as the circulatory system, immune system, and reproductive system of Hu sheep. The above results confirmed our speculation that *CHST3* can affect reproductive, digestive and immune performances of Hu sheep. In the human PheWAS data, *CHST3* was also significantly associated with similar traits that affect immune, metabolic, and activity performances in humans. Comparison of the data from the sheep GWAS and human PheWAS, revealed that *CHST3* shows tissue specificity and conserved functions among mammals. Furthermore, the effects of *CHST3* on body size traits in Hu sheep was further verified by combining the phenotypic data. At the SNP25_ 27304876 locus, there were three genotypes: TT, TC, and CC. Among the BW, BH, BL, CC, CBC traits, individuals with the TT genotype had the highest average phenotype values, while those with the CC genotype had the lowest. There were significant differences between TT and TC, TT and CC genotypes for all traits except BL. This situation may be caused by the low genetic and phenotypic correlations between CBC and BL. These results can demonstrate that *CHST3* is a key candidate gene affecting body size in Hu sheep.

Sheep LS is a low heritability trait, with an estimated heritability of approximately 0.1. In this study, we had estimated the heritability of LS in Hu sheep to be 2.22E-03. This might be due to the relatively small number of SNPs, and the limited sample size of the Hu sheep population. The LS of ewes was regulated by multiple genes, and *BMPR1B* had been demonstrated to affect it. However, there are many genes that influenced LS in sheep remain unknown. This study revealed *SCMH1* could be a candidate gene for the LS trait in Hu sheep, and provided a reference for subsequent researches on sheep LS trait. The genetic and phenotypic correlations between LS and TN traits in Hu sheep were close to moderate. It was reasonable to speculate that these two traits might be subject to a certain degree of co-selection. In future breeding programs, Hu sheep with a greater TN may exhibit higher reproductive performance.

LS and TN are economically important reproductive traits directly related to reproductive efficiency and profitability in livestock farming. Therefore, it is crucial to identify the key candidate genes that influence reproductive traits in Hu sheep. As the markers of active enhancers, eRNAs play important roles in gene regulation and are associated with various complex traits and characteristics [17]. The *SCMH1* may affect the development process of oocytes and embryos [34]. In addition, *SCMH1* can regulate bone formation [35]. In this study, we revealed, for the first time, *SCMH1* is a key candidate gene affecting LS in Hu sheep. The eRNA in *SCMH1* was highly expressed in ovary, fallopian tube and placentome of cotyledonary placenta, suggesting that *SCMH1* could affect the reproductive performance in sheep. Subsequently, the gene expression level of *SCMH1* was characterized. We found that *SCMH1* had the highest expression level in the reproductive system in Hu sheep. At the same time, *SCMH1* was also highly expressed in tissues such as ovary and oviduct. It was further demonstrated that *SCMH1* could affect the reproductive performance of Hu sheep. In the human PheWAS data, *SCMH1* was significantly associated with age at menarche in humans. Maternal age at menarche influenced offspring birth weight [36]. The above results have suggested our conclusion that *SCMH1* is a key candidate gene affecting LS in Hu sheep, and *SCMH1* may also be a tissue-specific gene in mammals. The *BAZ2B* has been described as a master regulator in the reprogramming of human haematopoietic progenitors and has been associated with neurodevelopment [37,38]. Currently, there is no study showing that *BAZ2B* is related to reproductive performance in sheep. In this study, eRNA in *BAZ2B* was highly expressed in mammary gland, ovary and embryo, suggesting that *BAZ2B* could affect mammary gland and reproductive performance in sheep. Subsequently, the gene expression level of *BAZ2B* was characterized. We found that *BAZ2B* was expressed at the highest level in the reproductive system in Hu sheep. At the same time, *BAZ2B* was also highly expressed in tissues such as ovary and uterus. In the human PheWAS data, *BAZ2B* was significantly associated with number of live births in humans. The above results have suggested that *BAZ2B* not only affects the TN trait, but may also affects the LS trait in Hu sheep.

Through selection signature hotspots detection, we found two genes were related to growth and reproductive performances in sheep, including *GRID2* and *STPG2*. The *GRID2* has been identified as a candidate gene for growth traits in Akkaraman sheep [39]. The *STPG2* plays a role in testicular development and spermatogenesis, and it is associated with sheep fertility [40]. These findings demonstrated that Hu sheep population were suffering selection on body size and reproductive performance. Significant differences were observed between the candidate genes identified through GWAS and selection signature hotspots detection. This could be due to the different approaches used by each analysis: GWAS detected trait-related loci, whereas selection signature hotspots detection identified positive selection loci.

## CONCLUSION

We revealed, for the first time, *CHST3* is a key candidate gene affecting body size traits, *SCMH1* is a key candidate gene influencing LS trait, and *BAZ2B* is a key candidate gene impacting TN trait in Hu sheep. The findings not only contribute to the understanding of biological mechanisms involved in these traits, but also provide potential markers for selective breeding in Hu sheep.

## Figures and Tables

**Table 1:** Descriptive statistics of body size and reproductive traits in Hu sheep, and estimation of their heritability

Phenotype	Sample size	Mean	SD	CV (%)	h^2^
BW (kg)	556	50.25	6.74	13.43	0.18
BH (cm)	554	73.48	5.73	7.71	0.76
BL (cm)	556	74.33	4.53	6.17	0.19
CC (cm)	556	87.14	5.66	6.50	0.36
CBC (cm)	551	7.39	0.48	6.52	0.25
LS	428	2.42	0.84	34.67	2.22E-03
TN	521	2.44	0.79	32.59	0.40

SD, standard deviation; CV, coefficient of variation; BW, body weight; BH, body height; BL, body length; CC, chest circumference; CBC, cannon bone circumference; LS, litter size; TN, teat number.

**Table 2:** Genetic and phenotypic correlations of body size traits in Hu sheep

Phenotype	BW	BH	BL	CC	CBC
BW		0.99	−0.38	0.90	0.98
BH	0.40		−0.53	0.99	0.79
BL	0.29	−0.38		−0.56	−0.05
CC	0.65	0.43	0.21		0.73
CBC	0.48	0.40	0.18	0.52	

The values in the upper right corner are genetic correlations, and the values in the lower left corner are phenotypic correlations.

BW, body weight; BH, body height; BL, body length; CC, chest circumference; CBC, cannon bone circumference.

## Data Availability

The raw data used in this study are available in the NGDC BioProject Database under accession number PRJCA028270 and PRJCA046490. The phenotypic data used in this study can be found in Supplement 7.

## SUPPLEMENTARY MATERIAL

**Supplement 1**. The significant SNPs and their annotated genes.

**Supplement 2**. The QTL enrichment of significant SNPs.

**Supplement 3**. The QTL enrichment analysis was performed for all significant SNPs and ROH hotspots.

**Supplement 4**. The KEGG pathways enriched by *ADGRL4* and *FAT4*.

**Supplement 5**. The expression levels of *CHST3*, *SCMH1* and *BAZ2B* in tissues and systems.

**Supplement 6**. The genes were overlapped with the ROH, ROHet and IHS hotspots.

**Supplement 7**. Phenotypic data.

## References

[1] Lin C Li F Zhang X Expression and polymorphisms of CD8B gene and its associations with body weight and size traits in sheep Anim Biotechnol 2023 34 1214 22 10.1080/10495398.2021.2016432 34928779

[2] Qiu Z Cai W Liu Q Unravelling novel and pleiotropic genes for cannon bone circumference and bone mineral density in Yorkshire pigs J Anim Sci 2024 102 skae036 10.1093/jas/skae036 38330300 PMC10914368

[3] Pasandideh M Gholizadeh M Rahimi-Mianji G Identification of two novel SNPs affecting lambing traits in sheep by using a 50K SNP-chip Small Rumin Res 2020 191 106193 10.1016/j.smallrumres.2020.106193

[4] Zhao Y Pu Y Liang B A study using single-locus and multi-locus genome-wide association study to identify genes associated with teat number in Hu sheep Anim Genet 2022 53 203 11 10.1111/age.13169 35040155 PMC9303709

[5] Pendergrass SA Brown-Gentry K Dudek S Phenome-wide association study (phewas) for detection of pleiotropy within the population architecture using genomics and epidemiology (page) network PLOS Genet 2013 9 e1003087 10.1371/journal.pgen.1003087 23382687 PMC3561060

[6] Huang JY Labrecque JA From GWAS to PheWAS: the search for causality in big data Lancet Digit Health 2019 1 E101 3 10.1016/S2589-7500(19)30059-7 33323255

[7] Purcell S Neale B Todd-Brown K Plink: a tool set for whole-genome association and population-based linkage analyses Am J Hum Genet 2007 81 559 75 10.1086/519795 17701901 PMC1950838

[8] Yin L Zhang H Tang Z HIBLUP: an integration of statistical models on the BLUP framework for efficient genetic evaluation using big genomic data Nucleic Acids Res 2023 51 3501 12 10.1093/nar/gkad074 36809800 PMC10164590

[9] Kassahun D Taye M Kebede D Phenotypic and genetic parameter estimates for early growth, growth rate and growth efficiency-related traits of Fogera cattle in Ethiopia Vet Med Sci 2022 8 387 97 10.1002/vms3.628 34480429 PMC8788963

[10] Yin L Zhang H Tang Z rMVP: a memory-efficient, visualization-enhanced, and parallel-accelerated tool for genome-wide association study Genom Proteom Bioinform 2021 19 619 28 10.1016/j.gpb.2020.10.007 PMC9040015 33662620

[11] Yu J Pressoir G Briggs WH A unified mixed-model method for association mapping that accounts for multiple levels of relatedness Nat Genet 2006 38 203 8 10.1038/ng1702 16380716

[12] Turner SD qqman: an R package for visualizing GWAS results using Q-Q and manhattan plots J Open Source Softw 2018 3 731 10.21105/joss.00731

[13] Wang K Li M Hakonarson H Annovar: functional annotation of genetic variants from high-throughput sequencing data Nucleic Acids Res 2010 38 e164 10.1093/nar/gkq603 20601685 PMC2938201

[14] Fonseca PAS Suárez-Vega A Marras G Cánovas Á Gallo: an R package for genomic annotation and integration of multiple data sources in livestock for positional candidate loci GigaScience 2020 9 giaa149 10.1093/gigascience/giaa149 33377911 PMC7772745

[15] Kuhn RM Haussler D Kent WJ The UCSC genome browser and associated tools Brief Bioinform 2013 14 144 61 10.1093/bib/bbs038 22908213 PMC3603215

[16] Jin W Zhu Q Yang Y Animal-APAdb: a comprehensive animal alternative polyadenylation database Nucleic Acids Res 2021 49 D47 54 10.1093/nar/gkaa778 32986825 PMC7779049

[17] Jin W Jiang G Yang Y Animal-eRNAdb: a comprehensive animal enhancer RNA database Nucleic Acids Res 2022 50 D46 53 10.1093/nar/gkab832 34551433 PMC8728245

[18] Wang Y Huang Y Zhen Y De novo transcriptome assembly database for 100 tissues from each of seven species of domestic herbivore Sci Data 2024 11 488 10.1038/s41597-024-03338-5 38734729 PMC11088706

[19] Watanabe K Stringer S Frei O A global overview of pleiotropy and genetic architecture in complex traits Nat Genet 2019 51 1339 48 10.1038/s41588-019-0481-0 31427789

[20] Danecek P Auton A Abecasis G The variant call format and VCFtools Bioinformatics 2011 27 2156 8 10.1093/bioinformatics/btr330 21653522 PMC3137218

[21] Biscarini F Cozzi P Gaspa G Marras G detectRUNS: detect runs of homozygosity and runs of heterozygosity in diploid genomes [Internet] The R Project for Statistical Computing 2019 [cited 2025 Aug 20]. Available from: https://cran.r-project.org/web//packages//detectRUNS/vignettes/detectRUNS.vignette.html

[22] Szpiech ZA Hernandez RD Selscan: an efficient multithreaded program to perform EHH-based scans for positive selection Mol Biol Evol 2014 31 2824 7 10.1093/molbev/msu211 25015648 PMC4166924

[23] Wang W Zhang Y Zhang X Heritability and recursive influence of host genetics on the rumen microbiota drive body weight variance in male Hu sheep lambs Microbiome 2023 11 197 10.1186/s40168-023-01642-7 37644504 PMC10463499

[24] Favara DM Zois CE Haider S ADGRL4/ELTD1 silencing in endothelial cells induces ACLY and SLC25A1 and alters the cellular metabolic profile Metabolites 2019 9 287 10.3390/metabo9120287 31775252 PMC6950702

[25] Miao X Luo Q Zhao H Qin X Comparative analysis of alternative splicing events in skeletal muscle of different sheep Heliyon 2023 9 e22118 10.1016/j.heliyon.2023.e22118 38034685 PMC10682031

[26] Rao-Bhatia A Zhu M Yin WC Hedgehog-activated Fat4 and PCP pathways mediate mesenchymal cell clustering and villus formation in gut development Dev Cell 2020 52 647 58.E6 10.1016/j.devcel.2020.02.003 32155439

[27] Zhang X Liu J Liang X History and progression of fat cadherins in health and disease Onco Targets Ther 2016 9 7337 43 10.2147/OTT.S111176 27942226 PMC5138043

[28] Taylor L Wankell M Saxena P McFarlane C Hebbard L Cell adhesion an important determinant of myogenesis and satellite cell activity Biochim Biophys Acta Mol Cell Res 2022 1869 119170 10.1016/j.bbamcr.2021.119170 34763027

[29] Luo Y Li J Li X The ARK2N–CK2 complex initiates transcription-coupled repair through enhancing the interaction of CSB with lesion-stalled RNAPII Proc Natl Acad Sci USA 2024 121 e2404383121 10.1073/pnas.2404383121 38843184 PMC11181095

[30] Du M Stitzinger SH Spille JH Direct observation of a condensate effect on super-enhancer controlled gene bursting Cell 2024 187 331 44.E17 10.1016/j.cell.2023.12.005 38194964

[31] Mitschka S Mayr C Context-specific regulation and function of mRNA alternative polyadenylation Nat Rev Mol Cell Biol 2022 23 779 96 10.1038/s41580-022-00507-5 35798852 PMC9261900

[32] Guan Y Sun C Zou F Carbohydrate sulfotransferase 3 (CHST3) overexpression promotes cartilage endplate-derived stem cells (CESCs) to regulate molecular mechanisms related to repair of intervertebral disc degeneration by rat nucleus pulposus J Cell Mol Med 2021 25 6006 17 10.1111/jcmm.16440 33993645 PMC8256370

[33] Begolli G Marković I Knežević J Debeljak Ž Carbohydrate sulfotransferases: a review of emerging diagnostic and prognostic applications Biochem Med 2023 33 030503 10.11613/BM.2023.030503 PMC10373059 37545696

[34] Nestorov P Hotz HR Liu Z Peters AHFM Dynamic expression of chromatin modifiers during developmental transitions in mouse preimplantation embryos Sci Rep 2015 5 14347 10.1038/srep14347 26403153 PMC4585904

[35] Pei YF Liu L Liu TL Joint association analysis identified 18 new loci for bone mineral density J Bone Miner Res 2019 34 1086 94 10.1002/jbmr.3681 30690781

[36] Reshetnikova Y Churnosova M Stepanov V Maternal age at menarche gene polymorphisms are associated with offspring birth weight Life 2023 13 1525 10.3390/life13071525 37511900 PMC10381708

[37] Arumugam K Shin W Schiavone V The master regulator protein BAZ2B can reprogram human hematopoietic lineage-committed progenitors into a multipotent state Cell Rep 2020 33 108474 10.1016/j.celrep.2020.108474 33296649 PMC8049840

[38] Arnold N Girke T Sureshchandra S Messaoudi I Acute simian varicella virus infection causes robust and sustained changes in gene expression in the sensory ganglia J Virol 2016 90 10823 43 10.1128/JVI.01272-16 27681124 PMC5110160

[39] Kizilaslan M Arzik Y White SN Piel LMW Cinar MU Genetic parameters and genomic regions underlying growth and linear type traits in Akkaraman sheep Genes 2022 13 1414 10.3390/genes13081414 36011330 PMC9407525

[40] Khalkhali-Evrigh R Hedayat N Seyedsharifi R Shakouri M Ponnampalam EN Genomic evidence of improved fertility and adaptation in Iranian domestic sheep attributed to introgression from Asiatic Mouflon and urial Sci Rep 2025 15 1185 10.1038/s41598-025-85756-y 39774243 PMC11707054

