# Supplementary material for: Genome-wide association study reveals candidate genes for body size and reproductive traits in Hu sheep
Source: Anim Biosci. 2025 Nov 10;39(5):250716. doi: 10.5713/ab.250716 (PMC13175056; doi:10.5713/ab.250716)
Supplement: Supplementary file 1 [file ab-250716-Supplement-1.pdf]

Supplement 1. The significant SNPs and their annotated genes.

Body weight

SNP	Chr	Position	P_value	Candidate gene	Location
12_18444340	12	18444340	0.000013532	gene-USH2A	intronic
7_62101896	7	62101896	3.79536E-05	gene-SEMA6D(dist=680572),gene-LOC101113241(dist=820745)	intergenic
7_72337476	7	72337476	4.15483E-05	gene-SYT16(dist=30885),gene-KCNH5(dist=689903)	intergenic
6_104980986	6	104980986	4.25426E-05	gene-MSX1(dist=25803),gene-TRNAY-GUA-14(dist=75820)	intergenic
12_59136085	12	59136085	4.77195E-05	gene-RASAL2	intronic
22_41380016	22	41380016	6.04729E-05	gene-NSMCE4A	intronic
17_32588634	17	32588634	9.75727E-05	gene-FAT4	intronic
17_32587075	17	32587075	0.000103115	gene-FAT4	intronic
25_34500809	25	34500809	0.000128496	gene-LOC105605755(dist=43616),gene-LOC101113398(dist=4714)	intergenic
6_75670580	6	75670580	0.000132408	NONE(dist=NONE),gene-TRNAC-ACA-99(dist=111062)	intergenic
11_51954794	11	51954794	0.000142085	gene-TBC1D16	intronic
1_14334383	1	14334383	0.000142792	gene-HPCAL4(dist=4934),gene-PPIE(dist=40995)	intergenic
17_32585911	17	32585911	0.0001539	gene-FAT4	intronic
1_315885	1	315885	0.000154412	gene-THAP4	intronic
17_32588404	17	32588404	0.000156396	gene-FAT4	intronic
24_29130422	24	29130422	0.000162202	gene-CALN1	intronic
7_79778028	7	79778028	0.000173524	gene-SMOC1	intronic
7_88781157	7	88781157	0.000180113	gene-NRXN3	intronic

Body height

SNP	Chr	Position	P_value	Candidate gene	Location
1_56344040	1	56344040	2.69E-13	gene-ADGRL4(dist=872910),gene-TRNAW-CCA-7(dist=576449)	intergenic
10_71516304	10	71516304	3.74E-12	gene-LOC105605916	intronic
3_125411220	3	125411220	3.87E-12	gene-TRNAG-CCC-53(dist=410492),gene-TRNAK-UUU-6(dist=239421)	intergenic
1_212395999	1	212395999	3.45E-10	gene-NAALADL2	intronic
7_93120304	7	93120304	5.54E-09	gene-LOC101113416(dist=712169),gene-TRNAK-UUU-13(dist=378228)	intergenic
12_7442165	12	7442165	6.15E-09	gene-LOC114117326(dist=21455),gene-TRNAK-UUU-19(dist=1526268)	intergenic
8_1232875	8	1232875	1.32E-08	gene-CD109(dist=704632),gene-COL12A1(dist=633171)	intergenic
7_94564447	7	94564447	3.61E-08	gene-TRNAW-CCA-109(dist=607940),gene-LOC114115898(dist=672238)	intergenic
18_49633052	18	49633052	3.78E-08	gene-LRFN5(dist=255686),gene-LOC114109129(dist=1109445)	intergenic

17_13982239	17	13982239	1.24E-07	gene-TRNAC-ACA-184(dist=329189),gene-LOC121816918(dist=119574)	intergenic
22_10076618	22	10076618	1.43E-07	gene-LOC101122743(dist=59061),gene-LOC114110286(dist=14451)	intergenic
1_57185605	1	57185605	5.54E-07	gene-TRNAW-CCA-7(dist=265045),gene-LOC114113047(dist=596022)	intergenic
5_89046410	5	89046410	8.21E-07	gene-TRNAH-GUG-15(dist=111763),gene-TRNAW-CCA-88(dist=664119)	intergenic
10_77250679	10	77250679	1.88E-06	gene-ITGBL1	intronic
2_250168038	2	250168038	2.24E-06	gene-LOC114112490(dist=31982),NONE(dist=NONE)	intergenic
2_191078176	2	191078176	2.90E-06	gene-CNTNAP5(dist=465289),gene-TRNAC-GCA-44(dist=354362)	intergenic
5_49146032	5	49146032	4.72E-06	gene-PFDN1	intronic
22_4937270	22	4937270	5.99E-06	gene-PCDH15	intronic
7_14792097	7	14792097	8.78E-06	gene-SKOR1(dist=148734),gene-PIAS1(dist=71964)	intergenic
22_2856864	22	2856864	1.06E-05	gene-ZWINT(dist=313556),gene-TRNAW-CCA-215(dist=158177)	intergenic
23_46304583	23	46304583	1.35E-05	gene-ARK2N	intronic
26_35256226	26	35256226	1.79E-05	gene-ZMAT4	intronic
2_190182972	2	190182972	2.04E-05	gene-CNTNAP5	intronic
8_33005695	8	33005695	3.38E-05	gene-LOC114116128(dist=206599),gene-TRNAM-CAU-6(dist=446329)	intergenic
9_54214558	9	54214558	3.68E-05	gene-PEX2(dist=1034836),gene-IL7(dist=903934)	intergenic
13_10312916	13	10312916	3.98E-05	gene-KIF16B(dist=106554),gene-SNRPB2(dist=75458)	intergenic
3_10412954	3	10412954	4.08E-05	gene-MAPKAP1	intronic
1_56910195	1	56910195	4.59E-05	gene-ADGRL4(dist=1439065),gene-TRNAW-CCA-7(dist=10294)	intergenic
6_11662397	6	11662397	5.39E-05	gene-UGT8(dist=255347),gene-LOC121819864(dist=7291)	intergenic
1_65587305	1	65587305	7.61E-05	gene-TRNAE-CUC(dist=616745),gene-PKN2(dist=346912)	intergenic
15_77266349	15	77266349	7.79E-05	gene-LOC101104526(dist=638)	downstream
4_17692256	4	17692256	0.000108586	gene-NXPH1(dist=26466),gene-TRNAH-GUG-14(dist=1368566)	intergenic
6_60692099	6	60692099	0.000110991	gene-APBB2	intronic
4_114701164	4	114701164	0.000128611	gene-LOC101105810	intronic
23_42363796	23	42363796	0.000137457	gene-RAB31	intronic
1_56213846	1	56213846	0.000159389	gene-ADGRL4(dist=742716),gene-TRNAW-CCA-7(dist=706643)	intergenic
25_12413788	25	12413788	0.000180995	gene-LOC101119014(dist=10083),gene-TRNAS-GGA-227(dist=4465)	intergenic

Body length

SNP	Chr	Position	P_value	Candidate gene	Location
14_31455302	14	31455302	1.52781E-08	gene-TRNAC-ACA-166(dist=659101),gene-CDH11(dist=823317)	intergenic
22_10076618	22	10076618	3.57227E-08	gene-LOC101122743(dist=59061),gene-LOC114110286(dist=14451)	intergenic
1_57185605	1	57185605	5.11687E-08	gene-TRNAW-CCA-7(dist=265045),gene-LOC114113047(dist=596022)	intergenic
7_93120304	7	93120304	5.36558E-08	gene-LOC101113416(dist=712169),gene-TRNAK-UUU-13(dist=378228)	intergenic
5_89046410	5	89046410	1.39098E-07	gene-TRNAH-GUG-15(dist=111763),gene-TRNAW-CCA-88(dist=664119)	intergenic
18_49633052	18	49633052	3.6543E-07	gene-LRFN5(dist=255686),gene-LOC114109129(dist=1109445)	intergenic

12_7442165	12	7442165	8.33948E-07	gene-LOC114117326(dist=21455),gene-TRNAK-UUU-19(dist=1526268)	intergenic
8_1232875	8	1232875	1.21865E-06	gene-CD109(dist=704632),gene-COL12A1(dist=633171)	intergenic
17_13982239	17	13982239	2.9898E-06	gene-TRNAC-ACA-184(dist=329189),gene-LOC121816918(dist=119574)	intergenic
9_52124523	9	52124523	6.60939E-06	gene-HNF4G(dist=298496),gene-ZFHX4(dist=700749)	intergenic
1_56344040	1	56344040	7.63749E-06	gene-ADGRL4(dist=872910),gene-TRNAW-CCA-7(dist=576449)	intergenic
5_49146032	5	49146032	8.85524E-06	gene-PFDN1	intronic
1_60394363	1	60394363	1.14551E-05	gene-ADGRL2(dist=1686211),gene-TTLL7(dist=457916)	intergenic
3_125411220	3	125411220	2.55825E-05	gene-TRNAG-CCC-53(dist=410492),gene-TRNAK-UUU-6(dist=239421)	intergenic
22_4937270	22	4937270	2.57087E-05	gene-PCDH15	intronic
2_88451621	2	88451621	2.99195E-05	gene-SLC24A2(dist=165523),gene-MLLT3(dist=304241)	intergenic
13_13918003	13	13918003	4.87501E-05	gene-TRNAG-UCC-66(dist=461664),gene-TRNAS-GGA-173(dist=181982)	intergenic
2_250168038	2	250168038	0.000055034	gene-LOC114112490(dist=31982),NONE(dist=NONE)	intergenic
23_46304583	23	46304583	5.72353E-05	gene-ARK2N	intronic
10_34162425	10	34162425	6.94424E-05	gene-NUP58	exonic
1_82584350	1	82584350	0.00008888	gene-TRNAS-GGA-8(dist=957666),gene-PRMT6(dist=1804422)	intergenic
3_195558466	3	195558466	9.70958E-05	gene-PDE3A(dist=463948),gene-AEBP2(dist=486266)	intergenic
1_21222623	1	21222623	0.000111205	gene-TEX38(dist=832)	upstream
3_142952162	3	142952162	0.000115163	gene-TWF1	intronic

Chest circumference

SNP	Chr	Position	P value	Candidate gene	Location
21_41951984	21	41951984	5.79E-06	gene-ALDH3B1	intronic
17_71853482	17	71853482	9.39E-06	gene-RAB36	intronic
23_46304583	23	46304583	1.42E-05	gene-ARK2N	intronic
7_93889841	7	93889841	4.21E-05	gene-TRNAK-UUU-13(dist=391237),gene-TRNAW-CCA-109(dist=66594)	intergenic
15_62261277	15	62261277	4.54E-05	gene-CCDC73	intronic
17_32588634	17	32588634	6.24E-05	gene-FAT4	intronic
2_215801506	2	215801506	8.62E-05	gene-SPAG16(dist=421888),gene-VWC2L(dist=591029)	intergenic
2_166373392	2	166373392	9.19E-05	gene-ARHGAP15	intronic
17_32585911	17	32585911	0.000124815	gene-FAT4	intronic
17_32587075	17	32587075	0.000136018	gene-FAT4	intronic
12_11400970	12	11400970	0.0001363	gene-UCHL5	intronic
6_75670580	6	75670580	0.000137193	NONE(dist=NONE),gene-TRNAC-ACA-99(dist=111062)	intergenic
16_69459341	16	69459341	0.000145053	gene-IRX1(dist=56206),gene-LOC121816827(dist=657428)	intergenic

Cannon bone circumference

SNP	Chr	Position	P_value	Candidate gene	Location

3_144238976	3	144238976	3.66E-06	gene-TRNAW-CCA-56(dist=133520),gene-TRNAC-GCA-70(dist=26597)	intergenic
20_20701313	20	20701313	5.69E-06	gene-ADGRF4	intronic
3_144217550	3	144217550	1.37E-05	gene-TRNAW-CCA-56(dist=112094),gene-TRNAC-GCA-70(dist=48023)	intergenic
25_27304876	25	27304876	4.18E-05	gene-CHST3	intronic
12_16133713	12	16133713	4.77E-05	gene-TRNAS-GGA-167(dist=809018),gene-LOC114117288(dist=178355)	intergenic
9_91700710	9	91700710	6.05E-05	gene-RALYL(dist=246486),gene-TRNAC-GCA-143(dist=1132261)	intergenic
17_20227379	17	20227379	8.01E-05	NONE(dist=NONE),gene-PCDH18(dist=130115)	intergenic
2_191458341	2	191458341	0.000110333	gene-TRNAC-GCA-44(dist=25732),gene-TRNAW-CCA-39(dist=18212)	intergenic
14_9762105	14	9762105	0.000141354	gene-HSBP1(dist=58381),gene-MLYCD(dist=14308)	intergenic
9_71795369	9	71795369	0.000152942	gene-ZFPM2	intronic
10_76629682	10	76629682	0.000159504	gene-TMTC4	intronic

Litter size

SNP	Chr	Position	P value	Candidate gene	Location
9_39666608	9	39666608	2.31E-05	gene-CHD7(dist=205004),gene-CLVS1(dist=130574)	intergenic
23_42530139	23	42530139	2.80E-05	gene-VAPA(dist=85640),gene-APCDD1(dist=111162)	intronic
23_42577279	23	42577279	3.37E-05	gene-VAPA(dist=132780),gene-APCDD1(dist=64022)	intronic
1_15813699	1	15813699	8.57E-05	gene-SCMH1(dist=52083),gene-TRNAG-CCC-3(dist=10221)	intergenic
9_36194232	9	36194232	8.59E-05	gene-LYN	intronic
18_48113701	18	48113701	0.000134473	gene-TRNAR-GCG-13(dist=420174),gene-TRNAW-CCA-197(dist=743596)	intergenic
10_49118417	10	49118417	0.000146821	gene-KLF12	intergenic
2_189839408	2	189839408	0.000166304	gene-CNTNAP5	intergenic

Teat number

SNP	Chr	Position	P value	Candidate gene	Location
1_176795571	1	176795571	5.23E-06	gene-TRNAS-GGA-24(dist=3088),gene-NECTIN3(dist=84401)	intergenic
2_150294372	2	150294372	8.35E-06	gene-BAZ2B	intronic
2_150205548	2	150205548	1.32E-05	gene-BAZ2B	intronic
13_81757521	13	81757521	1.77E-05	gene-PFDN4(dist=71023),gene-DOK5(dist=189658)	intergenic
21_2710850	21	2710850	4.59E-05	gene-TRNAW-CCA-210(dist=423518),gene-LOC106991818(dist=1958208)	intergenic
2_150246594	2	150246594	5.87E-05	gene-BAZ2B	intronic
1_250885969	1	250885969	8.82E-05	gene-FOXL2(dist=47897),gene-TRNAC-ACA-35(dist=9149)	intergenic
12_61859458	12	61859458	0.000135839	gene-LOC114117249(dist=58354),gene-LOC101102503(dist=169155)	intergenic
2_149990257	2	149990257	0.000155292	gene-MARCHF7	exonic
