# Supplementary material for: Genome-wide association study reveals candidate genes for body size and reproductive traits in Hu sheep
Source: Anim Biosci. 2025 Nov 10;39(5):250716. doi: 10.5713/ab.250716 (PMC13175056; doi:10.5713/ab.250716)
Supplement: Supplementary file 2 [file ab-250716-Supplement-2.pdf]

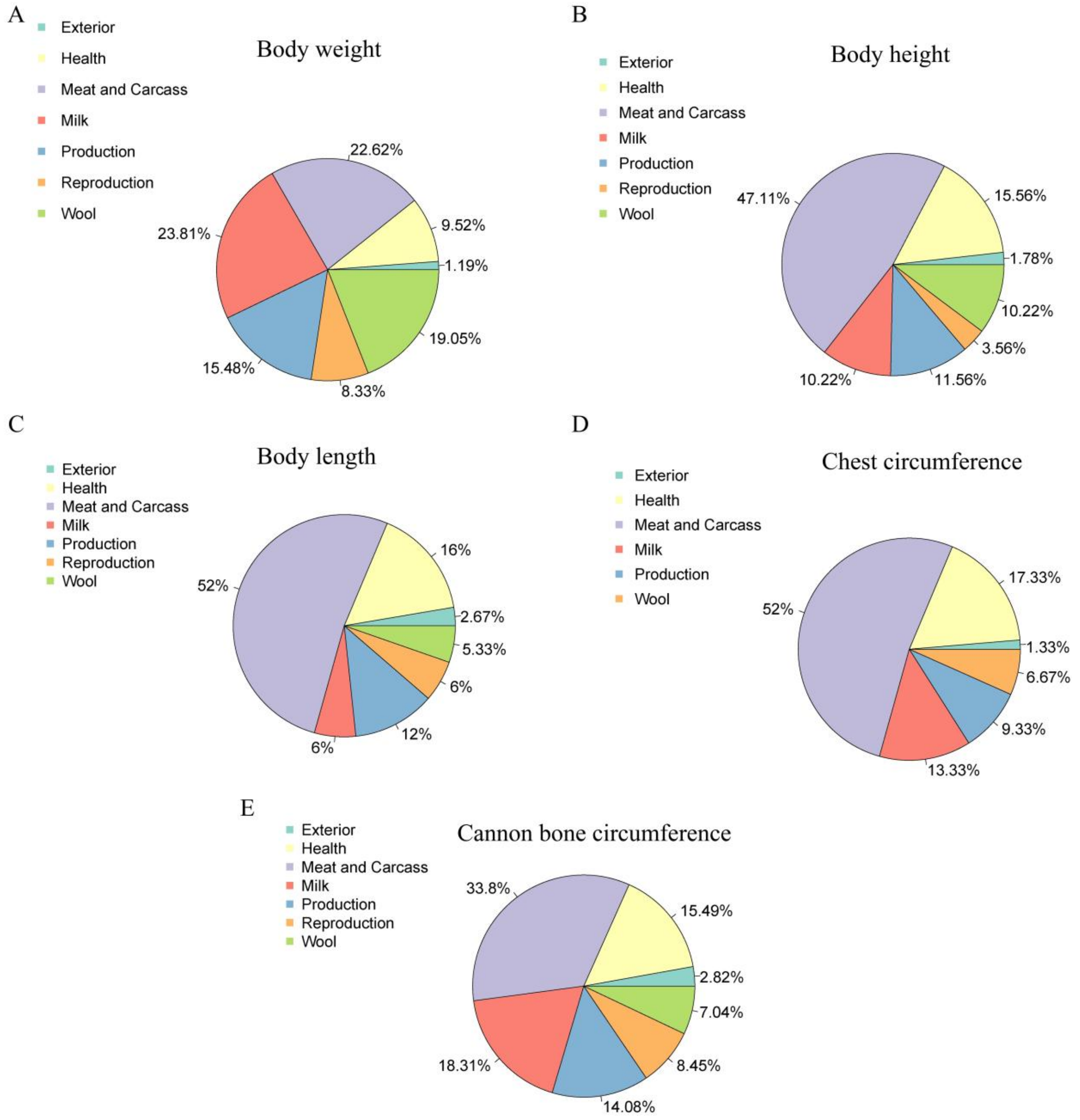

Supplement 2. The QTL enrichment of significant SNPs. A: BW: body weight. B: Body height. C: Body length. D: Chest circumference. E: Cannon bone circumference.
