# Supplementary material for: Genome-wide association study reveals candidate genes for body size and reproductive traits in Hu sheep
Source: Anim Biosci. 2025 Nov 10;39(5):250716. doi: 10.5713/ab.250716 (PMC13175056; doi:10.5713/ab.250716)
Supplement: Supplementary file 3 [file ab-250716-Supplement-3.pdf]

Supplement 3. **The QTL enrichment analysis was performed for all significant SNPs and ROH hotspots**.

Body weight

	CHR	BP	SNPid	QTL_type	start_pos	end_pos	Name
1	1	315885	1_315885	Meat_and_Carcass	0	184545242	muscle weight in carcass
2	1	14334383	1_14334383	Meat_and_Carcass	0	184545242	muscle weight in carcass
3	1	14334383	1_14334383	Meat_and_Carcass	6425670	194826314	Lean meat yield percentage
4	1	14334383	1_14334383	Meat_and_Carcass	12851340	264137874	Carcass fat percentage
5	6	75670580	6_75670580	Wool	0	118469638	Mean fiber diameter
6	6	104980986	6_104980986	Wool	0	118469638	Mean fiber diameter
7	6	75670580	6_75670580	Meat_and_Carcass	0	107665086	fat weight in carcass
8	6	104980986	6_104980986	Meat_and_Carcass	0	107665086	fat weight in carcass
9	6	75670580	6_75670580	Meat_and_Carcass	0	107665086	Carcass fat percentage
10	6	104980986	6_104980986	Meat_and_Carcass	0	107665086	Carcass fat percentage
11	6	75670580	6_75670580	Meat_and_Carcass	0	99827980	Lean meat yield percentage
12	6	75670580	6_75670580	Meat_and_Carcass	0	107665086	fat weight in carcass
13	6	104980986	6_104980986	Meat_and_Carcass	0	107665086	fat weight in carcass
14	6	75670580	6_75670580	Meat_and_Carcass	0	82099383	muscle weight in carcass
15	6	75670580	6_75670580	Production	2663094	91838698	Body weight
16	6	75670580	6_75670580	Health	67033880	76544930	Fecal egg count
17	6	75670580	6_75670580	Milk	74947074	104241108	Useful yield content
18	6	75670580	6_75670580	Exterior	75532502	75532506	Teat number
19	6	75670580	6_75670580	Health	75608588	75608592	Fecal egg count
20	6	75670580	6_75670580	Production	75608588	75608592	Average daily gain
21	6	75670580	6_75670580	Health	75608588	75608592	Hematocrit
22	6	104980986	6_104980986	Health	90316930	111089064	Trichostrongylus colubriformis FEC
23	6	104980986	6_104980986	Health	90316930	111089064	Trichostrongylus colubriformis FEC
24	6	104980986	6_104980986	Milk	105315939	105315943	Milk Yield
25	6	104980986	6_104980986	Milk	105315939	105315943	Milk protein yield
26	6	104980986	6_104980986	Milk	105333966	105333970	Milk Yield
27	6	104980986	6_104980986	Milk	105333966	105333970	Milk protein yield
28	7	62101896	7_62101896	Wool	0	101274392	Staple length
29	7	72337476	7_72337476	Wool	0	101274392	Staple length
30	7	79778028	7_79778028	Wool	0	101274392	Staple length

31	7	88781157	7_88781157	Wool	0	101274392	Staple length
32	7	62101896	7_62101896	Wool	0	101274392	Primary fiber diameter coefficient of variance
33	7	72337476	7_72337476	Wool	0	101274392	Primary fiber diameter coefficient of variance
34	7	79778028	7_79778028	Wool	0	101274392	Primary fiber diameter coefficient of variance
35	7	88781157	7_88781157	Wool	0	101274392	Primary fiber diameter coefficient of variance
36	7	62101896	7_62101896	Reproduction	61619872	61619876	Reproductive seasonality
37	7	62101896	7_62101896	Reproduction	61630024	61630028	Reproductive seasonality
38	7	62101896	7_62101896	Reproduction	61633432	61633436	Reproductive seasonality
39	7	62101896	7_62101896	Reproduction	61633502	61633506	Reproductive seasonality
40	11	51954794	11_51954794	Health	342412	55128460	Trichostrongylus adult and larva count
41	11	51954794	11_51954794	Meat_and_Carcass	27963712	52389158	Jaw length
42	11	51954794	11_51954794	Meat_and_Carcass	38007820	55128460	Hot carcass weight
43	11	51954794	11_51954794	Production	38007820	55128460	Body weight
44	11	51954794	11_51954794	Production	48451411	55128460	Body weight
45	11	51954794	11_51954794	Health	49079168	55356736	Salmonella abortusovis susceptibility
46	11	51954794	11_51954794	Milk	51396161	51510298	milk lauric acid content
47	12	18444340	12_18444340	Meat_and_Carcass	0	59547032	Carcass fat percentage
48	12	59136085	12_59136085	Meat_and_Carcass	0	59547032	Carcass fat percentage
49	12	18444340	12_18444340	Meat_and_Carcass	0	56902176	Lean meat yield percentage
50	12	18444340	12_18444340	Milk	17909450	40957476	Milk Yield
51	12	18444340	12_18444340	Production	18042016	18042020	Body weight
52	12	18444340	12_18444340	Reproduction	18736375	18736379	Total lambs born
53	22	41380016	22_41380016	Health	35853683	51512484	scrapie susceptibility
54	22	41380016	22_41380016	Meat_and_Carcass	41030730	41030734	Tail fat deposition
55	24	29130422	24_29130422	Meat_and_Carcass	313457	34898257	Bone weight in carcass
56	24	29130422	24_29130422	Production	20009030	42630206	bone density
57	24	29130422	24_29130422	Production	20009030	42630206	bone density
58	24	29130422	24_29130422	Reproduction	20009030	32599569	Reproductive seasonality
59	24	29130422	24_29130422	Milk	20108292	42577963	Milk cis-10 heptadecenoic acid content
60	24	29130422	24_29130422	Milk	20108292	42577963	Milk cis-10 heptadecenoic acid content
61	24	29130422	24_29130422	Milk	20108292	42577963	milk conjugated linoleic acid content
62	24	29130422	24_29130422	Milk	20108292	42577963	Milk fat percentage
63	24	29130422	24_29130422	Milk	20108292	42577963	Milk fat percentage
64	24	29130422	24_29130422	Milk	20108292	42577963	milk capric acid content
65	24	29130422	24_29130422	Milk	20108292	42577963	milk myristic acid content
66	24	29130422	24_29130422	Milk	20108292	42577963	Milk pentadecylic acid content
67	24	29130422	24_29130422	Milk	20108292	42577963	Milk pentadecylic acid content

68	24	29130422	24_29130422	Milk	20108292	42577963	milk palmitic acid content
69	24	29130422	24_29130422	Milk	20108292	42577963	milk palmitic acid content
70	24	29130422	24_29130422	Production	24548938	30927796	Body weight
71	24	29130422	24_29130422	Production	24548938	30927796	Body weight
72	24	29130422	24_29130422	Production	24548938	30927796	Body weight
73	24	29130422	24_29130422	Production	24548938	30927796	Average daily gain
74	24	29130422	24_29130422	Production	24548938	30927796	Average daily gain
75	24	29130422	24_29130422	Production	24548938	30927796	Average daily gain
76	24	29130422	24_29130422	Wool	29590024	29590028	Staple length
77	25	34500809	25_34500809	Reproduction	0	49921588	Testes weight
78	25	34500809	25_34500809	Wool	0	44863742	Staple length
79	25	34500809	25_34500809	Wool	0	44863742	Mean fiber diameter
80	25	34500809	25_34500809	Wool	0	44863742	Primary fiber diameter coefficient of variance
81	25	34500809	25_34500809	Wool	34531287	34662660	Primary fiber diameter
82	25	34500809	25_34500809	Wool	34531287	34662660	Staple strength
83	25	34500809	25_34500809	Milk	34778469	34778473	Cheese yield
84	25	34500809	25_34500809	Milk	34778469	34778473	Dried curd yield

Body height

	CHR	BP	SNPid	QTL_type	start_pos	end_pos	Name
1	1	56213846	1_56213846	Meat_and_Carcass	0	184545242	muscle weight in carcass
2	1	56344040	1_56344040	Meat_and_Carcass	0	184545242	muscle weight in carcass
3	1	56910195	1_56910195	Meat_and_Carcass	0	184545242	muscle weight in carcass
4	1	57185605	1_57185605	Meat_and_Carcass	0	184545242	muscle weight in carcass
5	1	65587305	1_65587305	Meat_and_Carcass	0	184545242	muscle weight in carcass
6	1	56213846	1_56213846	Meat_and_Carcass	6425670	194826314	Lean meat yield percentage
7	1	56344040	1_56344040	Meat_and_Carcass	6425670	194826314	Lean meat yield percentage
8	1	56910195	1_56910195	Meat_and_Carcass	6425670	194826314	Lean meat yield percentage
9	1	57185605	1_57185605	Meat_and_Carcass	6425670	194826314	Lean meat yield percentage
10	1	65587305	1_65587305	Meat_and_Carcass	6425670	194826314	Lean meat yield percentage
11	1	56213846	1_56213846	Meat_and_Carcass	12851340	264137874	Carcass fat percentage
12	1	56344040	1_56344040	Meat_and_Carcass	12851340	264137874	Carcass fat percentage
13	1	56910195	1_56910195	Meat_and_Carcass	12851340	264137874	Carcass fat percentage
14	1	57185605	1_57185605	Meat_and_Carcass	12851340	264137874	Carcass fat percentage
15	1	65587305	1_65587305	Meat_and_Carcass	12851340	264137874	Carcass fat percentage
16	1	212395999	1_212395999	Meat_and_Carcass	12851340	264137874	Carcass fat percentage
17	1	56213846	1_56213846	Meat_and_Carcass	38211317	236807358	Bone weight in carcass

18	1	56344040	1_56344040	Meat_and_Carcass	38211317	236807358	Bone weight in carcass
19	1	56910195	1_56910195	Meat_and_Carcass	38211317	236807358	Bone weight in carcass
20	1	57185605	1_57185605	Meat_and_Carcass	38211317	236807358	Bone weight in carcass
21	1	65587305	1_65587305	Meat_and_Carcass	38211317	236807358	Bone weight in carcass
22	1	212395999	1_212395999	Meat_and_Carcass	38211317	236807358	Bone weight in carcass
23	1	56213846	1_56213846	Meat_and_Carcass	42837800	239891680	Meat eicosapentaenoic acid content
24	1	56344040	1_56344040	Meat_and_Carcass	42837800	239891680	Meat eicosapentaenoic acid content
25	1	56910195	1_56910195	Meat_and_Carcass	42837800	239891680	Meat eicosapentaenoic acid content
26	1	57185605	1_57185605	Meat_and_Carcass	42837800	239891680	Meat eicosapentaenoic acid content
27	1	65587305	1_65587305	Meat_and_Carcass	42837800	239891680	Meat eicosapentaenoic acid content
28	1	212395999	1_212395999	Meat_and_Carcass	42837800	239891680	Meat eicosapentaenoic acid content
29	1	56213846	1_56213846	Meat_and_Carcass	42837800	245032216	Meat docosapentaenoic acid content
30	1	56344040	1_56344040	Meat_and_Carcass	42837800	245032216	Meat docosapentaenoic acid content
31	1	56910195	1_56910195	Meat_and_Carcass	42837800	245032216	Meat docosapentaenoic acid content
32	1	57185605	1_57185605	Meat_and_Carcass	42837800	245032216	Meat docosapentaenoic acid content
33	1	65587305	1_65587305	Meat_and_Carcass	42837800	245032216	Meat docosapentaenoic acid content
34	1	212395999	1_212395999	Meat_and_Carcass	42837800	245032216	Meat docosapentaenoic acid content
35	1	56213846	1_56213846	Meat_and_Carcass	44379960	69225884	Carcass bone percentage
36	1	56344040	1_56344040	Meat_and_Carcass	44379960	69225884	Carcass bone percentage
37	1	56910195	1_56910195	Meat_and_Carcass	44379960	69225884	Carcass bone percentage
38	1	57185605	1_57185605	Meat_and_Carcass	44379960	69225884	Carcass bone percentage
39	1	65587305	1_65587305	Meat_and_Carcass	44379960	69225884	Carcass bone percentage
40	1	56213846	1_56213846	Meat_and_Carcass	47978336	245032216	Meat polyunsaturated fatty acid content
41	1	56344040	1_56344040	Meat_and_Carcass	47978336	245032216	Meat polyunsaturated fatty acid content
42	1	56910195	1_56910195	Meat_and_Carcass	47978336	245032216	Meat polyunsaturated fatty acid content
43	1	57185605	1_57185605	Meat_and_Carcass	47978336	245032216	Meat polyunsaturated fatty acid content
44	1	65587305	1_65587305	Meat_and_Carcass	47978336	245032216	Meat polyunsaturated fatty acid content
45	1	212395999	1_212395999	Meat_and_Carcass	47978336	245032216	Meat polyunsaturated fatty acid content
46	1	56910195	1_56910195	Reproduction	57074940	57074944	Total lambs born
47	1	57185605	1_57185605	Reproduction	57074940	57074944	Total lambs born
48	1	56213846	1_56213846	Production	45013960	88588570	Body weight
49	1	56344040	1_56344040	Production	45013960	88588570	Body weight
50	1	56910195	1_56910195	Production	45013960	88588570	Body weight
51	1	57185605	1_57185605	Production	45013960	88588570	Body weight
52	1	65587305	1_65587305	Production	45013960	88588570	Body weight
53	1	212395999	1_212395999	Wool	150874731	227554393	Fleece yield
54	1	212395999	1_212395999	Health	173064712	220186292	Salmonella abortusovis susceptibility

55	1	212395999	1_212395999	Meat_and_Carcass	175806331	218472780	Lean meat yield percentage
56	1	212395999	1_212395999	Meat_and_Carcass	194483612	213160892	Muscle depth at third lumbar
57	1	212395999	1_212395999	Reproduction	195340368	271420300	Reproductive seasonality
58	1	212395999	1_212395999	Health	200994957	220100616	Fecal egg count
59	1	212395999	1_212395999	Wool	203907928	227725744	Fleece yield
60	1	212395999	1_212395999	Production	212464966	212464970	Body height
61	2	190182972	2_190182972	Meat_and_Carcass	0	198447000	Meat arachidonic acid content
62	2	191078176	2_191078176	Meat_and_Carcass	0	198447000	Meat arachidonic acid content
63	2	190182972	2_190182972	Meat_and_Carcass	4762728	233373672	Meat linolenic acid content
64	2	191078176	2_191078176	Meat_and_Carcass	4762728	233373672	Meat linolenic acid content
65	2	190182972	2_190182972	Meat_and_Carcass	4762728	233373672	Meat eicosapentaenoic acid content
66	2	191078176	2_191078176	Meat_and_Carcass	4762728	233373672	Meat eicosapentaenoic acid content
67	2	190182972	2_190182972	Meat_and_Carcass	4762728	230198520	Meat docosapentaenoic acid content
68	2	191078176	2_191078176	Meat_and_Carcass	4762728	230198520	Meat docosapentaenoic acid content
69	2	190182972	2_190182972	Milk	11430547	250201977	Milk fat percentage
70	2	191078176	2_191078176	Milk	11430547	250201977	Milk fat percentage
71	2	250168038	2_250168038	Milk	11430547	250201977	Milk fat percentage
72	2	190182972	2_190182972	Production	31910277	247423719	Body weight
73	2	191078176	2_191078176	Production	31910277	247423719	Body weight
74	2	190182972	2_190182972	Meat_and_Carcass	54136341	248376265	Hot carcass weight
75	2	191078176	2_191078176	Meat_and_Carcass	54136341	248376265	Hot carcass weight
76	2	190182972	2_190182972	Milk	119306336	195748120	milk lactose yield
77	2	191078176	2_191078176	Milk	119306336	195748120	milk lactose yield
78	2	190182972	2_190182972	Milk	136293399	196621287	Milk Yield
79	2	191078176	2_191078176	Milk	136293399	196621287	Milk Yield
80	2	190182972	2_190182972	Meat_and_Carcass	179157951	208289971	Hot carcass weight
81	2	191078176	2_191078176	Meat_and_Carcass	179157951	208289971	Hot carcass weight
82	2	190182972	2_190182972	Production	179157951	208289971	Body weight
83	2	191078176	2_191078176	Production	179157951	208289971	Body weight
84	2	190182972	2_190182972	Production	190457085	190457089	Body weight
85	2	250168038	2_250168038	Production	211068229	250201977	bone density
86	3	10412954	3_10412954	Health	0	37394800	Haemonchus contortus FEC
87	3	10412954	3_10412954	Wool	0	226088960	Staple length
88	3	125411220	3_125411220	Wool	0	226088960	Staple length
89	3	125411220	3_125411220	Meat_and_Carcass	59457732	177101772	internal fat amount
90	3	125411220	3_125411220	Meat_and_Carcass	62075368	153318680	Meat conjugated linoleic acid content
91	3	125411220	3_125411220	Health	111885241	134172542	Trichostrongylus colubriformis FEC

92	3	125411220	3_125411220	Health	125784727	125784731	Fecal egg count
93	4	17692256	4_17692256	Wool	0	121578063	Primary fiber diameter coefficient of variance
94	4	114701164	4_114701164	Wool	0	121578063	Primary fiber diameter coefficient of variance
95	4	17692256	4_17692256	Production	9377469	43103454	Body weight
96	4	17692256	4_17692256	Health	9377469	31175971	Haemonchus contortus FEC
97	4	17692256	4_17692256	Milk	17204467	17204471	Milk fat percentage
98	5	49146032	5_49146032	Production	0	87175936	Body weight
99	5	49146032	5_49146032	Meat_and_Carcass	0	94667618	Meat palmitoleic acid content
100	5	89046410	5_89046410	Meat_and_Carcass	0	94667618	Meat palmitoleic acid content
101	5	49146032	5_49146032	Exterior	43792286	59456712	Foot angle
102	5	49146032	5_49146032	Wool	45426835	49172676	Mean fiber diameter
103	5	49146032	5_49146032	Reproduction	49550985	49550989	Total lambs born
104	5	89046410	5_89046410	Production	86767298	108220751	Body weight
105	5	89046410	5_89046410	Production	86767298	97187547	Age at maximum daily gain
106	5	89046410	5_89046410	Production	89175084	89175088	Average daily gain
107	5	89046410	5_89046410	Production	89175084	89175088	Kleiber ratio
108	6	11662397	6_11662397	Wool	0	118469638	Mean fiber diameter
109	6	60692099	6_60692099	Wool	0	118469638	Mean fiber diameter
110	6	11662397	6_11662397	Meat_and_Carcass	0	107665086	fat weight in carcass
111	6	60692099	6_60692099	Meat_and_Carcass	0	107665086	fat weight in carcass
112	6	11662397	6_11662397	Meat_and_Carcass	0	107665086	Carcass fat percentage
113	6	60692099	6_60692099	Meat_and_Carcass	0	107665086	Carcass fat percentage
114	6	11662397	6_11662397	Meat_and_Carcass	0	99827980	Lean meat yield percentage
115	6	60692099	6_60692099	Meat_and_Carcass	0	99827980	Lean meat yield percentage
116	6	11662397	6_11662397	Meat_and_Carcass	0	107665086	fat weight in carcass
117	6	60692099	6_60692099	Meat_and_Carcass	0	107665086	fat weight in carcass
118	6	11662397	6_11662397	Meat_and_Carcass	0	82099383	muscle weight in carcass
119	6	60692099	6_60692099	Meat_and_Carcass	0	82099383	muscle weight in carcass
120	6	11662397	6_11662397	Meat_and_Carcass	1978298	13848088	internal fat amount
121	6	11662397	6_11662397	Meat_and_Carcass	1978298	13848088	Carcass fat percentage
122	6	11662397	6_11662397	Meat_and_Carcass	1978298	34239780	Lean meat yield percentage
123	6	11662397	6_11662397	Meat_and_Carcass	1978298	34239780	fat weight in carcass
124	6	11662397	6_11662397	Meat_and_Carcass	1978298	34239780	fat weight in carcass
125	6	11662397	6_11662397	Production	2663094	91838698	Body weight
126	6	60692099	6_60692099	Production	2663094	91838698	Body weight
127	6	11662397	6_11662397	Production	9815403	13848088	Body weight
128	6	11662397	6_11662397	Reproduction	12017023	12017027	Total lambs born

129	6	60692099	6_60692099	Meat_and_Carcass	15217680	61022896	Hot carcass weight
130	6	60692099	6_60692099	Health	22598254	67033880	facial eczema susceptibility
131	6	60692099	6_60692099	Health	34239780	61555515	Fecal egg count
132	6	60692099	6_60692099	Health	60548685	60548689	Fecal egg count
133	6	60692099	6_60692099	Health	60774421	60774425	Fecal egg count
134	6	60692099	6_60692099	Health	60892852	60892856	Fecal egg count
135	7	14792097	7_14792097	Exterior	0	19995550	Teat placement
136	7	14792097	7_14792097	Wool	0	101274392	Staple length
137	7	93120304	7_93120304	Wool	0	101274392	Staple length
138	7	94564447	7_94564447	Wool	0	101274392	Staple length
139	7	14792097	7_14792097	Meat_and_Carcass	0	39649880	Longissimus muscle area
140	7	14792097	7_14792097	Wool	0	101274392	Primary fiber diameter coefficient of variance
141	7	93120304	7_93120304	Wool	0	101274392	Primary fiber diameter coefficient of variance
142	7	94564447	7_94564447	Wool	0	101274392	Primary fiber diameter coefficient of variance
143	7	14792097	7_14792097	Meat_and_Carcass	11328537	52411545	Longissimus muscle area
144	7	14792097	7_14792097	Milk	14481345	14481349	Milk Yield
145	7	14792097	7_14792097	Milk	14762344	14762348	Milk Yield
146	7	14792097	7_14792097	Production	14837040	14837044	Body weight
147	7	14792097	7_14792097	Production	15184960	15184964	Body weight
148	7	14792097	7_14792097	Production	15184960	15184964	Body weight
149	7	94564447	7_94564447	Milk	94140835	94140839	Milk fat yield
150	7	94564447	7_94564447	Milk	94948683	94948687	Milk fat yield
151	7	94564447	7_94564447	Milk	94948683	94948687	Milk Yield
152	8	1232875	8_1232875	Health	0	91792861	Trichostrongylus adult and larva count
153	8	33005695	8_33005695	Health	0	91792861	Trichostrongylus adult and larva count
154	8	1232875	8_1232875	Health	0	91792861	Trichostrongylus adult and larva count
155	8	33005695	8_33005695	Health	0	91792861	Trichostrongylus adult and larva count
156	8	1232875	8_1232875	Meat_and_Carcass	0	81234527	internal fat amount
157	8	33005695	8_33005695	Meat_and_Carcass	0	81234527	internal fat amount
158	8	33005695	8_33005695	Milk	21978572	61554367	Milk fat percentage
159	9	54214558	9_54214558	Meat_and_Carcass	2925140	88129230	muscle weight in carcass
160	9	54214558	9_54214558	Meat_and_Carcass	2925140	88129230	Longissimus muscle area
161	9	54214558	9_54214558	Meat_and_Carcass	12375594	88129230	Hot carcass weight
162	9	54214558	9_54214558	Meat_and_Carcass	45002160	75378618	muscle weight in carcass
163	9	54214558	9_54214558	Meat_and_Carcass	45002160	67203225	Longissimus muscle area
164	10	71516304	10_71516304	Health	17688794	80591871	Fecal egg count
165	10	77250679	10_77250679	Health	17688794	80591871	Fecal egg count

166	10	71516304	10_71516304	Meat_and_Carcass	71790617	76191244	fat weight in carcass
167	10	71516304	10_71516304	Meat_and_Carcass	71790617	76191244	Carcass fat percentage
168	10	71516304	10_71516304	Meat_and_Carcass	71790617	76191244	Lean meat yield percentage
169	12	7442165	12_7442165	Meat_and_Carcass	0	59547032	Carcass fat percentage
170	12	7442165	12_7442165	Meat_and_Carcass	0	56902176	Lean meat yield percentage
171	12	7442165	12_7442165	Health	7232623	7232627	Fecal egg count
172	13	10312916	13_10312916	Meat_and_Carcass	325455	63463725	muscle weight in carcass
173	13	10312916	13_10312916	Wool	10119203	10119207	Fiber diameter coefficient of variance
174	13	10312916	13_10312916	Wool	10119203	10119207	Fiber diameter standard deviation
175	13	10312916	13_10312916	Health	10808404	10808408	Haemonchus contortus resistance
176	13	10312916	13_10312916	Health	10808404	10808408	Haemonchus contortus resistance
177	13	10312916	13_10312916	Health	10808404	10808408	Haemonchus contortus resistance
178	13	10312916	13_10312916	Health	10808404	10808408	Haemonchus contortus resistance
179	13	10312916	13_10312916	Health	10808404	10808408	Haemonchus contortus resistance
180	13	10312916	13_10312916	Health	10808404	10808408	Haemonchus contortus resistance
181	13	10312916	13_10312916	Health	10808437	10808441	Haemonchus contortus resistance
182	13	10312916	13_10312916	Health	10808437	10808441	Haemonchus contortus resistance
183	13	10312916	13_10312916	Health	10808437	10808441	Haemonchus contortus resistance
184	13	10312916	13_10312916	Health	10808437	10808441	Haemonchus contortus resistance
185	13	10312916	13_10312916	Health	10808437	10808441	Haemonchus contortus resistance
186	15	77266349	15_77266349	Wool	35335577	92672551	Staple length
187	15	77266349	15_77266349	Meat_and_Carcass	69937774	82205219	Hind leg length
188	17	13982239	17_13982239	Health	3770920	27803505	Fecal egg count
189	18	49633052	18_49633052	Reproduction	0	57496608	Testes weight
190	18	49633052	18_49633052	Reproduction	0	57496608	Testes weight
191	18	49633052	18_49633052	Wool	11233133	109190317	Staple length
192	18	49633052	18_49633052	Meat_and_Carcass	12244648	55899480	Meat gadoleic acid content
193	18	49633052	18_49633052	Meat_and_Carcass	13309400	56964232	Meat docosahexaenoic acid content
194	18	49633052	18_49633052	Health	40992952	49191542	Haemonchus contortus FEC
195	18	49633052	18_49633052	Meat_and_Carcass	47221751	49191542	Carcass fat percentage
196	22	4937270	22_4937270	Health	4784633	26408692	Somatic cell score
197	22	10076618	22_10076618	Health	4784633	26408692	Somatic cell score
198	22	10076618	22_10076618	Health	9650046	9774323	Worm count
199	22	10076618	22_10076618	Health	10513766	10638042	Haemonchus contortus FEC
200	23	42363796	23_42363796	Meat_and_Carcass	0	50681790	Hot carcass weight
201	23	46304583	23_46304583	Meat_and_Carcass	0	50681790	Hot carcass weight
202	23	42363796	23_42363796	Production	0	50681790	Body weight

203	23	46304583	23_46304583	Production	0	50681790	Body weight
204	23	42363796	23_42363796	Meat_and_Carcass	0	50681790	Carcass fat percentage
205	23	46304583	23_46304583	Meat_and_Carcass	0	50681790	Carcass fat percentage
206	23	42363796	23_42363796	Meat_and_Carcass	0	50681790	Lean meat yield percentage
207	23	46304583	23_46304583	Meat_and_Carcass	0	50681790	Lean meat yield percentage
208	23	42363796	23_42363796	Meat_and_Carcass	0	50681790	fat weight in carcass
209	23	46304583	23_46304583	Meat_and_Carcass	0	50681790	fat weight in carcass
210	23	42363796	23_42363796	Milk	39295132	62440561	Milk Yield
211	23	46304583	23_46304583	Milk	39295132	62440561	Milk Yield
212	23	42363796	23_42363796	Milk	39295132	62440561	Milk fat yield
213	23	46304583	23_46304583	Milk	39295132	62440561	Milk fat yield
214	23	46304583	23_46304583	Milk	46088949	46088953	Milk fat yield
215	23	46304583	23_46304583	Milk	46519403	46519407	Milk Yield
216	25	12413788	25_12413788	Wool	0	15370594	Greasy fleece weight
217	25	12413788	25_12413788	Reproduction	0	49921588	Testes weight
218	25	12413788	25_12413788	Wool	0	44863742	Staple length
219	25	12413788	25_12413788	Wool	0	44863742	Mean fiber diameter
220	25	12413788	25_12413788	Wool	0	44863742	Primary fiber diameter coefficient of variance
221	25	12413788	25_12413788	Milk	3152942	16355888	Milk fat percentage
222	25	12413788	25_12413788	Milk	3678432	18457850	Useful yield content
223	25	12413788	25_12413788	Milk	12476953	12476957	Milk fat yield
224	26	35256226	26_35256226	Exterior	25472609	45052301	udder attachment
225	26	35256226	26_35256226	Exterior	25472609	45052301	Stature

Body length

	CHR	BP	SNPid	QTL_type	start_pos	end_pos	Name
1	1	21222623	1_21222623	Meat_and_Carcass	0	184545242	muscle weight in carcass
2	1	56344040	1_56344040	Meat_and_Carcass	0	184545242	muscle weight in carcass
3	1	57185605	1_57185605	Meat_and_Carcass	0	184545242	muscle weight in carcass
4	1	60394363	1_60394363	Meat_and_Carcass	0	184545242	muscle weight in carcass
5	1	82584350	1_82584350	Meat_and_Carcass	0	184545242	muscle weight in carcass
6	1	21222623	1_21222623	Meat_and_Carcass	6425670	194826314	Lean meat yield percentage
7	1	56344040	1_56344040	Meat_and_Carcass	6425670	194826314	Lean meat yield percentage
8	1	57185605	1_57185605	Meat_and_Carcass	6425670	194826314	Lean meat yield percentage
9	1	60394363	1_60394363	Meat_and_Carcass	6425670	194826314	Lean meat yield percentage
10	1	82584350	1_82584350	Meat_and_Carcass	6425670	194826314	Lean meat yield percentage
11	1	21222623	1_21222623	Meat_and_Carcass	12851340	264137874	Carcass fat percentage

12	1	56344040	1_56344040	Meat_and_Carcass	12851340	264137874	Carcass fat percentage
13	1	57185605	1_57185605	Meat_and_Carcass	12851340	264137874	Carcass fat percentage
14	1	60394363	1_60394363	Meat_and_Carcass	12851340	264137874	Carcass fat percentage
15	1	82584350	1_82584350	Meat_and_Carcass	12851340	264137874	Carcass fat percentage
16	1	56344040	1_56344040	Meat_and_Carcass	38211317	236807358	Bone weight in carcass
17	1	57185605	1_57185605	Meat_and_Carcass	38211317	236807358	Bone weight in carcass
18	1	60394363	1_60394363	Meat_and_Carcass	38211317	236807358	Bone weight in carcass
19	1	82584350	1_82584350	Meat_and_Carcass	38211317	236807358	Bone weight in carcass
20	1	56344040	1_56344040	Meat_and_Carcass	42837800	239891680	Meat eicosapentaenoic acid content
21	1	57185605	1_57185605	Meat_and_Carcass	42837800	239891680	Meat eicosapentaenoic acid content
22	1	60394363	1_60394363	Meat_and_Carcass	42837800	239891680	Meat eicosapentaenoic acid content
23	1	82584350	1_82584350	Meat_and_Carcass	42837800	239891680	Meat eicosapentaenoic acid content
24	1	56344040	1_56344040	Meat_and_Carcass	42837800	245032216	Meat docosapentaenoic acid content
25	1	57185605	1_57185605	Meat_and_Carcass	42837800	245032216	Meat docosapentaenoic acid content
26	1	60394363	1_60394363	Meat_and_Carcass	42837800	245032216	Meat docosapentaenoic acid content
27	1	82584350	1_82584350	Meat_and_Carcass	42837800	245032216	Meat docosapentaenoic acid content
28	1	56344040	1_56344040	Meat_and_Carcass	44379960	69225884	Carcass bone percentage
29	1	57185605	1_57185605	Meat_and_Carcass	44379960	69225884	Carcass bone percentage
30	1	60394363	1_60394363	Meat_and_Carcass	44379960	69225884	Carcass bone percentage
31	1	56344040	1_56344040	Meat_and_Carcass	47978336	245032216	Meat polyunsaturated fatty acid content
32	1	57185605	1_57185605	Meat_and_Carcass	47978336	245032216	Meat polyunsaturated fatty acid content
33	1	60394363	1_60394363	Meat_and_Carcass	47978336	245032216	Meat polyunsaturated fatty acid content
34	1	82584350	1_82584350	Meat_and_Carcass	47978336	245032216	Meat polyunsaturated fatty acid content
35	1	57185605	1_57185605	Reproduction	57074940	57074944	Total lambs born
36	1	82584350	1_82584350	Milk	82162900	87303436	Milk Yield
37	1	82584350	1_82584350	Meat_and_Carcass	82162900	144963115	Muscle depth at third lumbar
38	1	82584350	1_82584350	Meat_and_Carcass	82162900	144963115	Backfat at third lumbar
39	1	82584350	1_82584350	Meat_and_Carcass	82162900	144963115	Muscle depth at third lumbar
40	1	82584350	1_82584350	Health	68591885	84878816	Immunoglobulin A level
41	1	56344040	1_56344040	Production	45013960	88588570	Body weight
42	1	57185605	1_57185605	Production	45013960	88588570	Body weight
43	1	60394363	1_60394363	Production	45013960	88588570	Body weight
44	1	82584350	1_82584350	Production	45013960	88588570	Body weight
45	2	88451621	2_88451621	Meat_and_Carcass	0	198447000	Meat arachidonic acid content
46	2	88451621	2_88451621	Meat_and_Carcass	4762728	233373672	Meat linolenic acid content
47	2	88451621	2_88451621	Meat_and_Carcass	4762728	233373672	Meat eicosapentaenoic acid content
48	2	88451621	2_88451621	Meat_and_Carcass	4762728	230198520	Meat docosapentaenoic acid content

49	2	88451621	2_88451621	Milk	11430547	250201977	Milk fat percentage
50	2	250168038	2_250168038	Milk	11430547	250201977	Milk fat percentage
51	2	88451621	2_88451621	Production	31910277	247423719	Body weight
52	2	88451621	2_88451621	Meat_and_Carcass	54136341	248376265	Hot carcass weight
53	2	88451621	2_88451621	Health	58343418	130339989	Trichostrongylus adult and larva count
54	2	88451621	2_88451621	Meat_and_Carcass	76997436	172251996	Ultimate pH
55	2	88451621	2_88451621	Meat_and_Carcass	76997436	172251996	Meat color a*
56	2	88451621	2_88451621	Meat_and_Carcass	82553952	157963812	Meat color L*
57	2	88451621	2_88451621	Meat_and_Carcass	87316680	172251996	Meat color b*
58	2	88451621	2_88451621	Meat_and_Carcass	87316680	172251996	Meat color a*
59	2	88451621	2_88451621	Meat_and_Carcass	87316680	172251996	Meat color b*
60	2	250168038	2_250168038	Production	211068229	250201977	bone density
61	3	125411220	3_125411220	Wool	0	226088960	Staple length
62	3	142952162	3_142952162	Wool	0	226088960	Staple length
63	3	195558466	3_195558466	Wool	0	226088960	Staple length
64	3	125411220	3_125411220	Meat_and_Carcass	59457732	177101772	internal fat amount
65	3	142952162	3_142952162	Meat_and_Carcass	59457732	177101772	internal fat amount
66	3	125411220	3_125411220	Meat_and_Carcass	62075368	153318680	Meat conjugated linoleic acid content
67	3	142952162	3_142952162	Meat_and_Carcass	62075368	153318680	Meat conjugated linoleic acid content
68	3	125411220	3_125411220	Health	111885241	134172542	Trichostrongylus colubriformis FEC
69	3	125411220	3_125411220	Health	125784727	125784731	Fecal egg count
70	3	142952162	3_142952162	Health	127665847	154814472	Change in eosinophil number
71	3	142952162	3_142952162	Production	132975908	146213668	Body weight
72	3	142952162	3_142952162	Production	134172542	161096798	Body weight
73	3	142952162	3_142952162	Health	140754027	147335512	Immunoglobulin A level
74	3	142952162	3_142952162	Milk	140754027	152645573	Milk protein percentage
75	3	195558466	3_195558466	Reproduction	178672354	198641177	Reproductive seasonality
76	3	195558466	3_195558466	Reproduction	178672354	198641177	Reproductive seasonality
77	3	195558466	3_195558466	Health	180242936	198641177	Nematodirus FEC
78	3	195558466	3_195558466	Health	193555484	209036932	Fecal oocyst count
79	3	195558466	3_195558466	Health	193555484	208438615	Fecal egg count
80	3	195558466	3_195558466	Milk	195212876	195212880	Milk fat yield
81	3	195558466	3_195558466	Reproduction	195212876	195212880	Total lambs born
82	3	195558466	3_195558466	Reproduction	195929465	195929469	Total lambs born
83	5	49146032	5_49146032	Production	0	87175936	Body weight
84	5	49146032	5_49146032	Meat_and_Carcass	0	94667618	Meat palmitoleic acid content
85	5	89046410	5_89046410	Meat_and_Carcass	0	94667618	Meat palmitoleic acid content

86	5	49146032	5_49146032	Exterior	43792286	59456712	Foot angle
87	5	49146032	5_49146032	Wool	45426835	49172676	Mean fiber diameter
88	5	49146032	5_49146032	Reproduction	49550985	49550989	Total lambs born
89	5	89046410	5_89046410	Production	86767298	108220751	Body weight
90	5	89046410	5_89046410	Production	86767298	97187547	Age at maximum daily gain
91	5	89046410	5_89046410	Production	89175084	89175088	Average daily gain
92	5	89046410	5_89046410	Production	89175084	89175088	Kleiber ratio
93	7	93120304	7_93120304	Wool	0	101274392	Staple length
94	7	93120304	7_93120304	Wool	0	101274392	Primary fiber diameter coefficient of variance
95	8	1232875	8_1232875	Health	0	91792861	Trichostrongylus adult and larva count
96	8	1232875	8_1232875	Health	0	91792861	Trichostrongylus adult and larva count
97	8	1232875	8_1232875	Meat_and_Carcass	0	81234527	internal fat amount
98	9	52124523	9_52124523	Meat_and_Carcass	2925140	88129230	muscle weight in carcass
99	9	52124523	9_52124523	Meat_and_Carcass	2925140	88129230	Longissimus muscle area
100	9	52124523	9_52124523	Meat_and_Carcass	12375594	88129230	Hot carcass weight
101	9	52124523	9_52124523	Meat_and_Carcass	45002160	75378618	muscle weight in carcass
102	9	52124523	9_52124523	Meat_and_Carcass	45002160	67203225	Longissimus muscle area
103	10	34162425	10_34162425	Exterior	4573200	35377588	Horns
104	10	34162425	10_34162425	Meat_and_Carcass	5867502	66009402	fat weight in carcass
105	10	34162425	10_34162425	Meat_and_Carcass	5867502	66009402	Carcass bone percentage
106	10	34162425	10_34162425	Meat_and_Carcass	5867502	66009402	Carcass fat percentage
107	10	34162425	10_34162425	Meat_and_Carcass	5867502	66009402	Lean meat yield percentage
108	10	34162425	10_34162425	Health	17688794	80591871	Fecal egg count
109	10	34162425	10_34162425	Health	22348281	35377588	Somatic cell score
110	10	34162425	10_34162425	Reproduction	24160304	62126496	Testes weight
111	10	34162425	10_34162425	Exterior	33617337	33789910	Horn length
112	10	34162425	10_34162425	Exterior	33617337	33789910	Horn circumference
113	10	34162425	10_34162425	Production	33721156	33721160	Body weight
114	10	34162425	10_34162425	Wool	34096751	34096755	Mean fiber diameter
115	12	7442165	12_7442165	Meat_and_Carcass	0	59547032	Carcass fat percentage
116	12	7442165	12_7442165	Meat_and_Carcass	0	56902176	Lean meat yield percentage
117	12	7442165	12_7442165	Health	7232623	7232627	Fecal egg count
118	13	13918003	13_13918003	Meat_and_Carcass	325455	63463725	muscle weight in carcass
119	13	13918003	13_13918003	Health	13864383	13994565	Change in hematocrit
120	14	31455302	14_31455302	Health	0	67691393	Nematodirus FEC
121	14	31455302	14_31455302	Meat_and_Carcass	0	53090191	Bone weight in carcass
122	14	31455302	14_31455302	Production	1734242	47216145	Total bone

123	14	31455302	14_31455302	Meat_and_Carcass	2069902	31216361	Dressing percentage
124	14	31455302	14_31455302	Production	6601309	31216361	Total bone
125	14	31455302	14_31455302	Meat_and_Carcass	6601309	31216361	fat weight in carcass
126	14	31455302	14_31455302	Meat_and_Carcass	6601309	31216361	Dressing percentage
127	14	31455302	14_31455302	Health	18349402	31719851	Fecal egg count
128	14	31455302	14_31455302	Health	19580155	52027269	Nematodirus FEC
129	14	31455302	14_31455302	Production	31128591	31128595	Body weight
130	17	13982239	17_13982239	Health	3770920	27803505	Fecal egg count
131	18	49633052	18_49633052	Reproduction	0	57496608	Testes weight
132	18	49633052	18_49633052	Reproduction	0	57496608	Testes weight
133	18	49633052	18_49633052	Wool	11233133	109190317	Staple length
134	18	49633052	18_49633052	Meat_and_Carcass	12244648	55899480	Meat gadoleic acid content
135	18	49633052	18_49633052	Meat_and_Carcass	13309400	56964232	Meat docosahexaenoic acid content
136	18	49633052	18_49633052	Health	40992952	49191542	Haemonchus contortus FEC
137	18	49633052	18_49633052	Meat_and_Carcass	47221751	49191542	Carcass fat percentage
138	22	4937270	22_4937270	Health	4784633	26408692	Somatic cell score
139	22	10076618	22_10076618	Health	4784633	26408692	Somatic cell score
140	22	10076618	22_10076618	Health	9650046	9774323	Worm count
141	22	10076618	22_10076618	Health	10513766	10638042	Haemonchus contortus FEC
142	23	46304583	23_46304583	Meat_and_Carcass	0	50681790	Hot carcass weight
143	23	46304583	23_46304583	Production	0	50681790	Body weight
144	23	46304583	23_46304583	Meat_and_Carcass	0	50681790	Carcass fat percentage
145	23	46304583	23_46304583	Meat_and_Carcass	0	50681790	Lean meat yield percentage
146	23	46304583	23_46304583	Meat_and_Carcass	0	50681790	fat weight in carcass
147	23	46304583	23_46304583	Milk	39295132	62440561	Milk Yield
148	23	46304583	23_46304583	Milk	39295132	62440561	Milk fat yield
149	23	46304583	23_46304583	Milk	46088949	46088953	Milk fat yield
150	23	46304583	23_46304583	Milk	46519403	46519407	Milk Yield

Chest circumference

	CHR	BP	SNPid	QTL_type	start_pos	end_pos	Name
1	2	166373392	2_166373392	Meat_and_Carcass	0	198447000	Meat arachidonic acid content
2	2	166373392	2_166373392	Meat_and_Carcass	4762728	233373672	Meat linolenic acid content
3	2	215801506	2_215801506	Meat_and_Carcass	4762728	233373672	Meat linolenic acid content
4	2	166373392	2_166373392	Meat_and_Carcass	4762728	233373672	Meat eicosapentaenoic acid content
5	2	215801506	2_215801506	Meat_and_Carcass	4762728	233373672	Meat eicosapentaenoic acid content
6	2	166373392	2_166373392	Meat_and_Carcass	4762728	230198520	Meat docosapentaenoic acid content

7	2	215801506	2_215801506	Meat_and_Carcass	4762728	230198520	Meat docosapentaenoic acid content
8	2	166373392	2_166373392	Milk	11430547	250201977	Milk fat percentage
9	2	215801506	2_215801506	Milk	11430547	250201977	Milk fat percentage
10	2	166373392	2_166373392	Production	31910277	247423719	Body weight
11	2	215801506	2_215801506	Production	31910277	247423719	Body weight
12	2	166373392	2_166373392	Meat_and_Carcass	54136341	248376265	Hot carcass weight
13	2	215801506	2_215801506	Meat_and_Carcass	54136341	248376265	Hot carcass weight
14	2	166373392	2_166373392	Meat_and_Carcass	76997436	172251996	Ultimate pH
15	2	166373392	2_166373392	Meat_and_Carcass	76997436	172251996	Meat color a*
16	2	166373392	2_166373392	Meat_and_Carcass	87316680	172251996	Meat color b*
17	2	166373392	2_166373392	Meat_and_Carcass	87316680	172251996	Meat color a*
18	2	166373392	2_166373392	Meat_and_Carcass	87316680	172251996	Meat color b*
19	2	166373392	2_166373392	Meat_and_Carcass	97635924	172251996	Shear force
20	2	166373392	2_166373392	Meat_and_Carcass	98429712	172251996	Meat color L*
21	2	166373392	2_166373392	Milk	119306336	195748120	milk lactose yield
22	2	166373392	2_166373392	Milk	136293399	196621287	Milk Yield
23	2	166373392	2_166373392	Health	166483975	166483979	Fecal egg count
24	2	166373392	2_166373392	Wool	166616907	166616911	Mean fiber diameter
25	2	166373392	2_166373392	Health	166659637	166659641	Fecal egg count
26	2	215801506	2_215801506	Health	209480653	235437520	Haemonchus contortus FEC
27	2	215801506	2_215801506	Health	209480653	235437520	Change in hematocrit
28	2	215801506	2_215801506	Health	209480653	235437520	Worm count
29	2	215801506	2_215801506	Health	209480653	235437520	Haemonchus contortus FEC
30	2	215801506	2_215801506	Health	209480653	235437520	Change in hematocrit
31	2	215801506	2_215801506	Production	211068229	250201977	bone density
32	2	215801506	2_215801506	Production	211068229	235437520	Body weight
33	6	75670580	6_75670580	Wool	0	118469638	Mean fiber diameter
34	6	75670580	6_75670580	Meat_and_Carcass	0	107665086	fat weight in carcass
35	6	75670580	6_75670580	Meat_and_Carcass	0	107665086	Carcass fat percentage
36	6	75670580	6_75670580	Meat_and_Carcass	0	99827980	Lean meat yield percentage
37	6	75670580	6_75670580	Meat_and_Carcass	0	107665086	fat weight in carcass
38	6	75670580	6_75670580	Meat_and_Carcass	0	82099383	muscle weight in carcass
39	6	75670580	6_75670580	Production	2663094	91838698	Body weight
40	6	75670580	6_75670580	Health	67033880	76544930	Fecal egg count
41	6	75670580	6_75670580	Milk	74947074	104241108	Useful yield content
42	6	75670580	6_75670580	Exterior	75532502	75532506	Teat number
43	6	75670580	6_75670580	Health	75608588	75608592	Fecal egg count

44	6	75670580	6_75670580	Production	75608588	75608592	Average daily gain
45	6	75670580	6_75670580	Health	75608588	75608592	Hematocrit
46	7	93889841	7_93889841	Wool	0	101274392	Staple length
47	7	93889841	7_93889841	Wool	0	101274392	Primary fiber diameter coefficient of variance
48	7	93889841	7_93889841	Milk	94140835	94140839	Milk fat yield
49	12	11400970	12_11400970	Meat_and_Carcass	0	59547032	Carcass fat percentage
50	12	11400970	12_11400970	Meat_and_Carcass	0	56902176	Lean meat yield percentage
51	15	62261277	15_62261277	Wool	35335577	92672551	Staple length
52	16	69459341	16_69459341	Meat_and_Carcass	1697694	75717152	Dressing percentage
53	16	69459341	16_69459341	Meat_and_Carcass	56957633	72915957	Lean meat yield percentage
54	16	69459341	16_69459341	Health	69144494	69144498	Monocyte number
55	17	71853482	17_71853482	Health	45701308	73279684	Somatic cell score
56	21	41951984	21_41951984	Meat_and_Carcass	0	56639430	Meat oleic acid content
57	21	41951984	21_41951984	Meat_and_Carcass	0	53492795	Meat cis-vaccenic acid content
58	21	41951984	21_41951984	Meat_and_Carcass	0	66708662	Meat gadoleic acid content
59	21	41951984	21_41951984	Meat_and_Carcass	0	53492795	Meat linoleic acid content
60	21	41951984	21_41951984	Meat_and_Carcass	0	55380776	Meat linolenic acid content
61	21	41951984	21_41951984	Meat_and_Carcass	0	45311544	Meat arachidonic acid content
62	21	41951984	21_41951984	Meat_and_Carcass	0	67337989	Meat docosapentaenoic acid content
63	21	41951984	21_41951984	Meat_and_Carcass	0	62932700	Meat palmitic acid content
64	21	41951984	21_41951984	Meat_and_Carcass	0	53492795	Meat stearic acid content
65	21	41951984	21_41951984	Meat_and_Carcass	3775962	62932700	Meat myristic acid content
66	21	41951984	21_41951984	Health	33228465	41787312	Change in eosinophil number
67	23	46304583	23_46304583	Meat_and_Carcass	0	50681790	Hot carcass weight
68	23	46304583	23_46304583	Production	0	50681790	Body weight
69	23	46304583	23_46304583	Meat_and_Carcass	0	50681790	Carcass fat percentage
70	23	46304583	23_46304583	Meat_and_Carcass	0	50681790	Lean meat yield percentage
71	23	46304583	23_46304583	Meat_and_Carcass	0	50681790	fat weight in carcass
72	23	46304583	23_46304583	Milk	39295132	62440561	Milk Yield
73	23	46304583	23_46304583	Milk	39295132	62440561	Milk fat yield
74	23	46304583	23_46304583	Milk	46088949	46088953	Milk fat yield
75	23	46304583	23_46304583	Milk	46519403	46519407	Milk Yield

Cannon bone circumference

	CHR	BP	SNPid	QTL_type	start_pos	end_pos	Name
1	2	191458341	2_191458341	Meat_and_Carcass	0	198447000	Meat arachidonic acid content
2	2	191458341	2_191458341	Meat_and_Carcass	4762728	233373672	Meat linolenic acid content

3	2	191458341	2_191458341	Meat_and_Carcass	4762728	233373672	Meat eicosapentaenoic acid content
4	2	191458341	2_191458341	Meat_and_Carcass	4762728	230198520	Meat docosapentaenoic acid content
5	2	191458341	2_191458341	Milk	11430547	250201977	Milk fat percentage
6	2	191458341	2_191458341	Production	31910277	247423719	Body weight
7	2	191458341	2_191458341	Meat_and_Carcass	54136341	248376265	Hot carcass weight
8	2	191458341	2_191458341	Milk	119306336	195748120	milk lactose yield
9	2	191458341	2_191458341	Milk	136293399	196621287	Milk Yield
10	2	191458341	2_191458341	Meat_and_Carcass	179157951	208289971	Hot carcass weight
11	2	191458341	2_191458341	Production	179157951	208289971	Body weight
12	3	144217550	3_144217550	Wool	0	226088960	Staple length
13	3	144238976	3_144238976	Wool	0	226088960	Staple length
14	3	144217550	3_144217550	Meat_and_Carcass	59457732	177101772	internal fat amount
15	3	144238976	3_144238976	Meat_and_Carcass	59457732	177101772	internal fat amount
16	3	144217550	3_144217550	Meat_and_Carcass	62075368	153318680	Meat conjugated linoleic acid content
17	3	144238976	3_144238976	Meat_and_Carcass	62075368	153318680	Meat conjugated linoleic acid content
18	3	144217550	3_144217550	Health	127665847	154814472	Change in eosinophil number
19	3	144238976	3_144238976	Health	127665847	154814472	Change in eosinophil number
20	3	144217550	3_144217550	Production	132975908	146213668	Body weight
21	3	144238976	3_144238976	Production	132975908	146213668	Body weight
22	3	144217550	3_144217550	Production	134172542	161096798	Body weight
23	3	144238976	3_144238976	Production	134172542	161096798	Body weight
24	3	144217550	3_144217550	Health	140754027	147335512	Immunoglobulin A level
25	3	144238976	3_144238976	Health	140754027	147335512	Immunoglobulin A level
26	3	144217550	3_144217550	Milk	140754027	152645573	Milk protein percentage
27	3	144238976	3_144238976	Milk	140754027	152645573	Milk protein percentage
28	9	71795369	9_71795369	Meat_and_Carcass	2925140	88129230	muscle weight in carcass
29	9	71795369	9_71795369	Meat_and_Carcass	2925140	88129230	Longissimus muscle area
30	9	71795369	9_71795369	Meat_and_Carcass	12375594	88129230	Hot carcass weight
31	9	71795369	9_71795369	Meat_and_Carcass	45002160	75378618	muscle weight in carcass
32	9	91700710	9_91700710	Milk	75378618	95179568	Milk fat yield
33	9	91700710	9_91700710	Production	92079758	92079762	Body weight
34	10	76629682	10_76629682	Health	17688794	80591871	Fecal egg count
35	10	76629682	10_76629682	Meat_and_Carcass	71790617	76191244	fat weight in carcass
36	10	76629682	10_76629682	Meat_and_Carcass	71790617	76191244	Carcass fat percentage
37	10	76629682	10_76629682	Meat_and_Carcass	71790617	76191244	Lean meat yield percentage
38	10	76629682	10_76629682	Milk	76198733	76198737	Cheese yield
39	10	76629682	10_76629682	Exterior	76672738	76672742	Teat number

40	12	16133713	12_16133713	Meat_and_Carcass	0	59547032	Carcass fat percentage
41	12	16133713	12_16133713	Meat_and_Carcass	0	56902176	Lean meat yield percentage
42	14	9762105	14_9762105	Health	0	67691393	Nematodirus FEC
43	14	9762105	14_9762105	Meat_and_Carcass	0	53090191	Bone weight in carcass
44	14	9762105	14_9762105	Production	1734242	47216145	Total bone
45	14	9762105	14_9762105	Meat_and_Carcass	2069902	31216361	Dressing percentage
46	14	9762105	14_9762105	Health	6601309	14265541	Fecal egg count
47	14	9762105	14_9762105	Production	6601309	31216361	Total bone
48	14	9762105	14_9762105	Meat_and_Carcass	6601309	31216361	fat weight in carcass
49	14	9762105	14_9762105	Meat_and_Carcass	6601309	31216361	Dressing percentage
50	14	9762105	14_9762105	Milk	9746109	9746113	Milk fat yield
51	14	9762105	14_9762105	Exterior	0	9846020	Extension
52	17	20227379	17_20227379	Health	3770920	27803505	Fecal egg count
53	17	20227379	17_20227379	Reproduction	16828437	27803505	Reproductive seasonality
54	17	20227379	17_20227379	Reproduction	16828437	27803505	Reproductive seasonality
55	17	20227379	17_20227379	Reproduction	16828437	27803505	Reproductive seasonality
56	17	20227379	17_20227379	Reproduction	16828437	27803505	Reproductive seasonality
57	20	20701313	20_20701313	Reproduction	0	26781664	Testes weight
58	20	20701313	20_20701313	Milk	0	35794724	Milk Yield
59	20	20701313	20_20701313	Milk	10918678	27760224	Milk Yield
60	20	20701313	20_20701313	Milk	11279200	25339574	milk lactose yield
61	20	20701313	20_20701313	Milk	11279200	26163625	Milk protein yield
62	20	20701313	20_20701313	Milk	12103252	25751600	Milk fat yield
63	20	20701313	20_20701313	Health	14420896	33992112	Salmonella abortusovis susceptibility
64	20	20701313	20_20701313	Health	15811482	28687282	Immunoglobulin A level
65	20	20701313	20_20701313	Meat_and_Carcass	15811482	29202314	Metacarpal length
66	20	20701313	20_20701313	Health	21144131	21144135	Fecal egg count
67	25	27304876	25_27304876	Reproduction	0	49921588	Testes weight
68	25	27304876	25_27304876	Wool	0	44863742	Staple length
69	25	27304876	25_27304876	Wool	0	44863742	Mean fiber diameter
70	25	27304876	25_27304876	Wool	0	44863742	Primary fiber diameter coefficient of variance
71	25	27304876	25_27304876	Production	27230830	27230834	Kleiber ratio

ROH hotspots

	CHR	BP	SNPid	QTL_type	start_pos	end_pos	Name
1	2	113761631	2_113761631	Exterior	113990787	113990791	Horn type
2	2	113761631	2_113761631	Exterior	114107782	114107786	Horn type

3	2	114969797	2_114969797	Exterior	114762328	114762332	Horn type
4	2	114969797	2_114969797	Exterior	114795342	114795346	Horn type
5	2	114969797	2_114969797	Exterior	114906776	114906780	Horn type
6	2	115650665	2_115650665	Exterior	115888204	115888208	Horn type
7	2	116006409	2_116006409	Exterior	115888204	115888208	Horn type
8	2	115650665	2_115650665	Exterior	116006407	116006411	Horn type
9	2	116006409	2_116006409	Exterior	116006407	116006411	Horn type
10	2	115650665	2_115650665	Exterior	116061619	116061623	Horn type
11	2	116006409	2_116006409	Exterior	116061619	116061623	Horn type
12	2	115650665	2_115650665	Exterior	116133033	116133037	Horn type
13	2	116006409	2_116006409	Exterior	116133033	116133037	Horn type
14	2	123810374	2_123810374	Wool	123930842	123930846	Staple length
15	2	124504982	2_124504982	Wool	124785191	124785195	Staple length
16	6	28663697	6_28663697	Milk	28183985	28183989	Milk fat yield
17	6	28663697	6_28663697	Milk	28786774	28786778	Milk fat yield
18	6	28663697	6_28663697	Production	29021921	29021925	Body height
19	6	29975188	6_29975188	Reproduction	30030916	30030920	Total lambs born
20	6	29975188	6_29975188	Reproduction	30037910	30037914	Total lambs born
21	6	29975188	6_29975188	Reproduction	30096782	30096786	Total lambs born
22	6	32311927	6_32311927	Milk	31878666	31878670	Milk fat yield
23	6	32311927	6_32311927	Milk	31929002	31929006	Milk fat yield
24	6	33282642	6_33282642	Milk	33025138	33025142	Milk yield persistency
25	6	36866991	6_36866991	Meat_and_Carcass	36786232	36786236	Bone area
26	6	37060235	6_37060235	Meat_and_Carcass	36786232	36786236	Bone area
27	6	36866991	6_36866991	Production	36786232	36786236	Total bone
28	6	37060235	6_37060235	Production	36786232	36786236	Total bone
29	6	36866991	6_36866991	Meat_and_Carcass	36786232	36786236	fat weight in carcass
30	6	37060235	6_37060235	Meat_and_Carcass	36786232	36786236	fat weight in carcass
31	6	36866991	6_36866991	Meat_and_Carcass	36786232	36786236	Total fat area
32	6	37060235	6_37060235	Meat_and_Carcass	36786232	36786236	Total fat area
33	6	36866991	6_36866991	Meat_and_Carcass	36816865	36816869	Bone area
34	6	37060235	6_37060235	Meat_and_Carcass	36816865	36816869	Bone area
35	6	36866991	6_36866991	Production	36816865	36816869	Total bone
36	6	37060235	6_37060235	Production	36816865	36816869	Total bone
37	6	36866991	6_36866991	Meat_and_Carcass	36816865	36816869	fat weight in carcass
38	6	37060235	6_37060235	Meat_and_Carcass	36816865	36816869	fat weight in carcass
39	6	36866991	6_36866991	Meat_and_Carcass	36816865	36816869	Total fat area

40	6	37060235	6_37060235	Meat_and_Carcass	36816865	36816869	Total fat area
41	6	36866991	6_36866991	Production	36866989	36866993	Body weight
42	6	37060235	6_37060235	Production	36866989	36866993	Body weight
43	6	36866991	6_36866991	Production	36903740	36903744	Body weight
44	6	37060235	6_37060235	Production	36903740	36903744	Body weight
45	6	36866991	6_36866991	Production	36946224	36946228	Body weight
46	6	37060235	6_37060235	Production	36946224	36946228	Body weight
47	6	36866991	6_36866991	Health	36992404	36992408	Fecal egg count
48	6	37060235	6_37060235	Health	36992404	36992408	Fecal egg count
49	6	36866991	6_36866991	Health	36992404	36992408	Fecal egg count
50	6	37060235	6_37060235	Health	36992404	36992408	Fecal egg count
51	6	36866991	6_36866991	Production	37239965	37239969	Body weight
52	6	37060235	6_37060235	Production	37239965	37239969	Body weight
53	6	36866991	6_36866991	Meat_and_Carcass	37290507	37290511	Tail fat deposition
54	6	37060235	6_37060235	Meat_and_Carcass	37290507	37290511	Tail fat deposition
55	6	37060235	6_37060235	Health	37370149	37370153	Somatic cell score
56	6	37060235	6_37060235	Production	37373370	37373374	Body weight
57	6	37060235	6_37060235	Production	37373370	37373374	Body weight
58	6	37060235	6_37060235	Production	37373370	37373374	Body weight
59	6	37060235	6_37060235	Production	37373370	37373374	Body weight
60	6	37060235	6_37060235	Production	37373370	37373374	Body weight
61	6	37060235	6_37060235	Meat_and_Carcass	37373370	37373374	Bone area
62	6	37060235	6_37060235	Production	37373370	37373374	Total bone
63	6	37060235	6_37060235	Meat_and_Carcass	37373370	37373374	fat weight in carcass
64	6	37060235	6_37060235	Milk	37376442	37376446	Curd firming time
65	6	37060235	6_37060235	Meat_and_Carcass	37465728	37465732	Bone area
66	6	37060235	6_37060235	Meat_and_Carcass	37465728	37465732	Total fat area
67	6	37060235	6_37060235	Meat_and_Carcass	37465728	37465732	fat weight in carcass
68	6	37060235	6_37060235	Production	37533593	37533597	Body weight
69	6	37060235	6_37060235	Meat_and_Carcass	37554217	37554221	Bone area
70	6	37060235	6_37060235	Meat_and_Carcass	37554217	37554221	Total fat area
71	6	37060235	6_37060235	Meat_and_Carcass	37554217	37554221	fat weight in carcass
72	6	37060235	6_37060235	Meat_and_Carcass	37554217	37554221	Dressing percentage
73	10	28952950	10_28952950	Exterior	29041333	29041337	Horn type
74	10	29041335	10_29041335	Exterior	29041333	29041337	Horn type
75	10	28952950	10_28952950	Exterior	29071491	29071495	Horn type
76	10	29041335	10_29041335	Exterior	29071491	29071495	Horn type

77	10	28952950	10_28952950	Exterior	29169262	29169266	Horn type
78	10	29041335	10_29041335	Exterior	29169262	29169266	Horn type
79	10	28952950	10_28952950	Exterior	29169262	29169266	Horn type
80	10	29041335	10_29041335	Exterior	29169262	29169266	Horn type
81	10	28952950	10_28952950	Exterior	29230971	29230975	Horn type
82	10	29041335	10_29041335	Exterior	29230971	29230975	Horn type
83	10	28952950	10_28952950	Exterior	29230971	29230975	Horn type
84	10	29041335	10_29041335	Exterior	29230971	29230975	Horn type
85	10	28952950	10_28952950	Exterior	29319349	29319353	Horn type
86	10	29041335	10_29041335	Exterior	29319349	29319353	Horn type
87	10	28952950	10_28952950	Exterior	29319349	29319353	Horn type
88	10	29041335	10_29041335	Exterior	29319349	29319353	Horn type
89	10	28952950	10_28952950	Exterior	29347136	29347140	Horn type
90	10	29041335	10_29041335	Exterior	29347136	29347140	Horn type
91	10	28952950	10_28952950	Exterior	29387527	29387531	Horn type
92	10	29041335	10_29041335	Exterior	29387527	29387531	Horn type
93	10	28952950	10_28952950	Exterior	29387527	29387531	Horn type
94	10	29041335	10_29041335	Exterior	29387527	29387531	Horn type
95	10	28952950	10_28952950	Exterior	29396316	29396320	Horn type
96	10	29041335	10_29041335	Exterior	29396316	29396320	Horn type
97	10	28952950	10_28952950	Exterior	29396316	29396320	Horn type
98	10	29041335	10_29041335	Exterior	29396316	29396320	Horn type
99	10	29041335	10_29041335	Exterior	29458415	29458419	Horn type
100	10	29041335	10_29041335	Exterior	29458415	29458419	Horn type
101	10	29041335	10_29041335	Exterior	29458415	29458419	Horn length
102	10	29041335	10_29041335	Exterior	29458415	29458419	Horn circumference
103	10	29041335	10_29041335	Meat_and_Carcass	29479763	29479767	Tail fat deposition
104	10	29041335	10_29041335	Exterior	29479763	29479767	Horn type
105	10	29041335	10_29041335	Exterior	29479763	29479767	Horn type
106	10	29041335	10_29041335	Exterior	29479763	29479767	Horn circumference
107	10	29041335	10_29041335	Meat_and_Carcass	29519929	29519933	Tail fat deposition
108	10	29041335	10_29041335	Exterior	29519929	29519933	Horn type
109	10	29041335	10_29041335	Exterior	29519929	29519933	Horn type
110	10	29041335	10_29041335	Exterior	29519929	29519933	Horn length
111	10	29041335	10_29041335	Exterior	29519929	29519933	Horn circumference
112	13	48968947	13_48968947	Meat_and_Carcass	49385798	49385802	Tail fat deposition
113	13	49472664	13_49472664	Meat_and_Carcass	49385798	49385802	Tail fat deposition

114	15	2997774	15_2997774	Exterior	2788989	2788993	Coat color
115	15	3655664	15_3655664	Meat_and_Carcass	3615914	3615918	Tail fat deposition
116	15	3655664	15_3655664	Meat_and_Carcass	3817272	3817276	Tail fat deposition
117	15	3655664	15_3655664	Reproduction	3988513	3988517	Total lambs born
