# Supplementary figures and images for: Genome-wide association study reveals candidate genes for body size and reproductive traits in Hu sheep
Source: Anim Biosci. 2025 Nov 10;39(5):250716. doi: 10.5713/ab.250716 (PMC13175056; doi:10.5713/ab.250716)

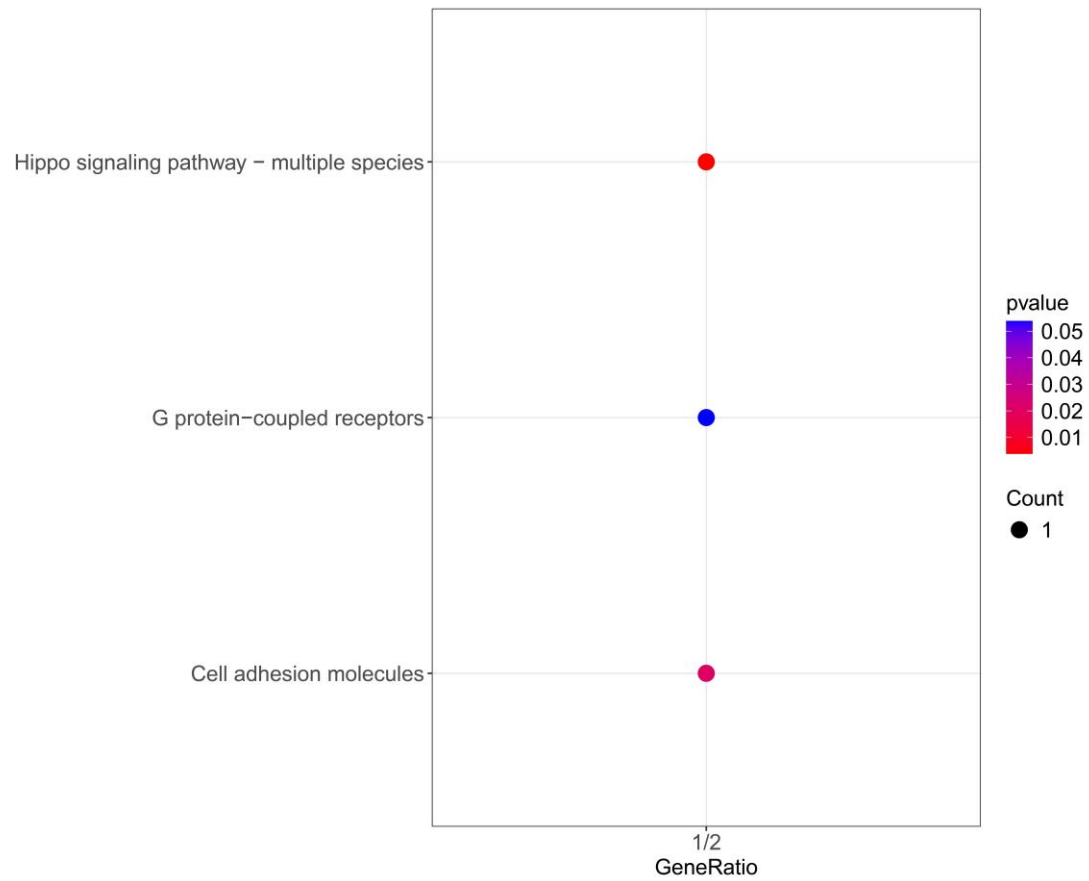

**Supplement 4.** The KEGG pathways enriched by *ADGRL4* and *FAT4*.

Supplement: Supplementary file 4 [file ab-250716-Supplement-4.pdf]
