# Supplementary material for: Genome-wide association study reveals candidate genes for body size and reproductive traits in Hu sheep
Source: Anim Biosci. 2025 Nov 10;39(5):250716. doi: 10.5713/ab.250716 (PMC13175056; doi:10.5713/ab.250716)
Supplement: Supplementary file 5 [file ab-250716-Supplement-5.pdf]

**Supplement 5. The expression levels of *CHST3* in tissues and systems.**

**tissues**

| Tissue | log2(TPM+1) |
|---|---|
| abomasum | 0.87 |
| latissimus dorsi | 2.43 |
| omasum | 1.99 |
| ovary | 2.87 |
| radial extensor of wrist | 2.09 |
| rhomboid muscle | 2.79 |
| rumen dorsal sac | 2.99 |
| rumen predorsum blind sac | 1.67 |
| rumen venter posterior blind sac | 2.04 |
| rumen ventral sac | 1.82 |
| sternocephalicus muscle | 3.23 |
| tail fat | 3.26 |
| tail muscle | 3.09 |
| uterus | 2.92 |

**Systems**

| System | TPM(median) |
|---|---|
| integumentary system | 10.76 |
| exercise system | 3.27 |
| digestive system | 3.49 |
| respiratory system | 8.73 |
| urinary system | 5.23 |
| reproductive system | 8.21 |
| circulatory system | 12.27 |
| immune system | 8.66 |
| nervous system | 1.66 |
| endocrine system | 2.89 |

**The expression levels of *SCMH1* in tissues and systems.**

**tissues**

| Tissue | log2(TPM+1) |
|---|---|
| cervix bottom | 3.98 |
| hypophysis | 3.06 |
| hypothalamus | 3.19 |
| ovary | 4.24 |
| oviduct | 4.70 |
| uterine caruncle | 3.41 |
| uterus | 3.23 |

**Systems**

| System | TPM(median) |
|---|---|
| integumentary system | 12.77 |
| exercise system | 12.95 |
| digestive system | 7.90 |
| respiratory system | 12.76 |
| urinary system | 12.94 |
| reproductive system | 16.31 |
| circulatory system | 14.89 |
| immune system | 8.31 |
| nervous system | 9.02 |

| | |
|---|---|
| endocrine system | 9.16 |

**The expression levels of *BAZ2B* in tissues and systems.**

**tissues**

| Tissue | log2(TPM+1) |
|---|---|
| cervix bottom | 4.59 |
| hypophysis | 3.61 |
| hypothalamus | 1.58 |
| ovary | 3.80 |
| oviduct | 3.59 |
| uterine caruncle | 3.66 |
| uterus | 3.76 |

**Systems**

| System | TPM(median) |
|---|---|
| integumentary system | 7.65 |
| exercise system | 4.00 |
| digestive system | 4.91 |
| respiratory system | 11.15 |
| urinary system | 7.34 |
| reproductive system | 12.12 |
| circulatory system | 4.31 |
| immune system | 3.78 |
| nervous system | 4.02 |
| endocrine system | 8.62 |
