# Supplementary material for: Genome-wide association study reveals candidate genes for body size and reproductive traits in Hu sheep
Source: Anim Biosci. 2025 Nov 10;39(5):250716. doi: 10.5713/ab.250716 (PMC13175056; doi:10.5713/ab.250716)
Supplement: Supplementary file 6 [file ab-250716-Supplement-6.pdf]

Supplement 6. The 35 genes were overlapped with the ROH hotspots.

	Chr	Pos	SNP	Strand	Gene_id	Gene_name
1	10	28952950	chr10_28952950	-	ENSOARG00020006253	NBEA
2	10	29041335	chr10_29041335	-	ENSOARG00020006253	NBEA
3	10	35990995	chr10_35990995	+	ENSOARG00020003659	AMER2
4	10	36817366	chr10_36817366	-	ENSOARG00020004193	SGCG
5	10	37584851	chr10_37584851	-	ENSOARG00020004477	SAP18
6	10	38752454	chr10_38752454	-	ENSOARG00020006165	ATP12A
7	10	39006711	chr10_39006711	-	ENSOARG00020006165	ATP12A
8	10	41977965	chr10_41977965	-	ENSOARG00020006377	PCDH9
9	10	42383810	chr10_42383810	-	ENSOARG00020006377	PCDH9
10	10	43046449	chr10_43046449	-	ENSOARG00020006377	PCDH9
11	12	54790540	chr12_54790540	-	ENSOARG00020009259	MMP23B
12	12	55137829	chr12_55137829	+	ENSOARG00020012032	C1orf159
13	13	48968947	chr13_48968947	-	ENSOARG00020011864	SLC23A2
14	13	49472664	chr13_49472664	-	ENSOARG00020012291	GPCPD1
15	15	1370476	chr15_1370476	+	ENSOARG00020012182	KBTBD3
16	15	2997774	chr15_2997774	-	ENSOARG00020012341	GRIA4
17	15	981917	chr15_981917	-	ENSOARG00020011841	GPR83
18	1	120590422	chr1_120590422	-	ENSOARG00020001009	OLFML2B
19	1	121062872	chr1_121062872	-	ENSOARG00020001174	SH2D1B
20	2	112836648	chr2_112836648	+	ENSOARG00020007527	C8orf74
21	2	113761631	chr2_113761631	+	ENSOARG00020007935	GATA4
22	2	114969797	chr2_114969797	-	ENSOARG00020009221	CEP44
23	2	115650665	chr2_115650665	+	ENSOARG00020009532	HAND2
24	2	116006409	chr2_116006409	-	ENSOARG00020009661	GALNT7
25	2	122926425	chr2_122926425	-	ENSOARG00020015487	OCA2
26	2	123810374	chr2_123810374	-	ENSOARG00020020859	TUBGCP5
27	2	124504982	chr2_124504982	-	ENSOARG00020021435	FAM168B
28	3	105989642	chr3_105989642	-	ENSOARG00020002664	IL1RL2
29	3	106858277	chr3_106858277	-	ENSOARG00020012320	RPL31
30	3	107139063	chr3_107139063	-	ENSOARG00020012376	NPAS2
31	5	47857002	chr5_47857002	+	ENSOARG00020021919	TCF7
32	5	48531657	chr5_48531657	-	ENSOARG00020009307	PITX1

33	6	28663697	chr6_28663697	+	ENSOARG00020014005	C4orf54
34	6	29975188	chr6_29975188	+	ENSOARG00020016727	TSPAN5
35	6	30720330	chr6_30720330	+	ENSOARG00020019402	STPG2
36	6	31015090	chr6_31015090	+	ENSOARG00020019402	STPG2
37	6	33282642	chr6_33282642	-	ENSOARG00020019674	PDHA2
38	6	34941975	chr6_34941975	-	ENSOARG00020020813	SMARCAD1
39	6	35045392	chr6_35045392	-	ENSOARG00020020813	SMARCAD1
40	6	36866991	chr6_36866991	-	ENSOARG00020021077	GRID2
41	6	37060235	chr6_37060235	-	ENSOARG00020021077	GRID2
42	6	79902281	chr6_79902281	-	ENSOARG00020020541	IGFBP7
43	6	80050455	chr6_80050455	-	ENSOARG00020020541	IGFBP7

The 9 genes were overlapped with the ROHet hotspots.

	Chr	Pos	SNP	Strand	Gene_id	Gene_name
1	10	38096414	10:38096414	-	ENSOARG00020005084	ZMYM2
2	10	38483187	10:38483187	-	ENSOARG00020005393	MPHOSPH8
3	10	38534888	10:38534888	-	ENSOARG00020006165	ATP12A
4	10	38692444	10:38692444	-	ENSOARG00020006165	ATP12A
5	10	42101629	10:42101629	-	ENSOARG00020006377	PCDH9
6	10	42195498	10:42195498	-	ENSOARG00020006377	PCDH9
7	10	42322459	10:42322459	-	ENSOARG00020006377	PCDH9
8	10	42383810	10:42383810	-	ENSOARG00020006377	PCDH9
9	10	42455743	10:42455743	-	ENSOARG00020006377	PCDH9
10	10	42461551	10:42461551	-	ENSOARG00020006377	PCDH9
11	10	42533379	10:42533379	-	ENSOARG00020006377	PCDH9
12	10	42573440	10:42573440	-	ENSOARG00020006377	PCDH9
13	10	42620184	10:42620184	-	ENSOARG00020006377	PCDH9
14	10	42653696	10:42653696	-	ENSOARG00020006377	PCDH9
15	10	42709029	10:42709029	-	ENSOARG00020006377	PCDH9
16	10	42833276	10:42833276	-	ENSOARG00020006377	PCDH9
17	10	42885724	10:42885724	-	ENSOARG00020006377	PCDH9
18	10	42955634	10:42955634	-	ENSOARG00020006377	PCDH9
19	10	43046449	10:43046449	-	ENSOARG00020006377	PCDH9
20	10	43117764	10:43117764	-	ENSOARG00020006377	PCDH9
21	10	43222291	10:43222291	-	ENSOARG00020006377	PCDH9

22	10	43315876	10:43315876	-	ENSOARG00020006377	PCDH9
23	10	43424975	10:43424975	-	ENSOARG00020006377	PCDH9
24	10	43459914	10:43459914	-	ENSOARG00020006377	PCDH9
25	10	43563550	10:43563550	-	ENSOARG00020006377	PCDH9
26	1	120590422	1:120590422	-	ENSOARG00020001009	OLFML2B
27	1	120649056	1:120649056	-	ENSOARG00020001009	OLFML2B
28	1	120692847	1:120692847	-	ENSOARG00020001009	OLFML2B
29	1	120804604	1:120804604	-	ENSOARG00020001009	OLFML2B
30	1	120821874	1:120821874	-	ENSOARG00020001009	OLFML2B
31	1	121198389	1:121198389	+	ENSOARG00020001187	UHMK1
32	1	121299657	1:121299657	+	ENSOARG00020001261	UAP1
33	2	123810374	2:123810374	-	ENSOARG00020020859	TUBGCP5
34	2	123900201	2:123900201	+	ENSOARG00020021252	IMP4
35	2	124045244	2:124045244	+	ENSOARG00020021252	IMP4
36	2	124132634	2:124132634	+	ENSOARG00020021252	IMP4
37	2	124195617	2:124195617	+	ENSOARG00020021252	IMP4

The 19 genes were overlapped with the IHS hotspots.

	Chr	Pos	SNP	Strand	Gene_id	Gene_name
1	15	3982222	chr15_3982222	+	ENSOARG00020014159	PDGFD
2	15	3982558	chr15_3982558	+	ENSOARG00020014159	PDGFD
3	15	3976400	chr15_3976400	+	ENSOARG00020014159	PDGFD
4	15	3976401	chr15_3976401	+	ENSOARG00020014159	PDGFD
5	15	3976420	chr15_3976420	+	ENSOARG00020014159	PDGFD
6	15	3967409	chr15_3967409	+	ENSOARG00020014159	PDGFD
7	6	36812093	chr6_36812093	-	ENSOARG00020021077	GRID2
8	6	31133129	chr6_31133129	+	ENSOARG00020019402	STPG2
9	6	36689107	chr6_36689107	-	ENSOARG00020021077	GRID2
10	6	36876520	chr6_36876520	-	ENSOARG00020021077	GRID2
11	13	48721849	chr13_48721849	+	ENSOARG00020011760	PRND
12	6	30868861	chr6_30868861	+	ENSOARG00020019402	STPG2
13	6	36145258	chr6_36145258	-	ENSOARG00020021077	GRID2
14	6	30720330	chr6_30720330	+	ENSOARG00020019402	STPG2
15	6	30186670	chr6_30186670	+	ENSOARG00020016727	TSPAN5
16	6	30109511	chr6_30109511	+	ENSOARG00020016727	TSPAN5

17	15	3977539	chr15_3977539	+	ENSOARG00020014159	PDGFD
18	6	30754891	chr6_30754891	+	ENSOARG00020019402	STPG2
19	12	55113896	chr12_55113896	-	ENSOARG00020011661	TTLL10
20	7	36377168	chr7_36377168	+	ENSOARG00020013321	SPINT1
21	13	48909330	chr13_48909330	-	ENSOARG00020011864	SLC23A2
22	1	121965000	chr1_121965000	+	ENSOARG00020001430	RGS4
23	6	29021716	chr6_29021716	+	ENSOARG00020014340	ADH7
24	5	19998772	chr5_19998772	+	ENSOARG00020023396	KIF3A
25	6	36370597	chr6_36370597	-	ENSOARG00020021077	GRID2
26	6	35981115	chr6_35981115	-	ENSOARG00020021077	GRID2
27	7	35627636	chr7_35627636	+	ENSOARG00020008995	EIF2AK4
28	6	29298070	chr6_29298070	+	ENSOARG00020015568	ADH4
29	6	29766047	chr6_29766047	-	ENSOARG00020016389	METAP1
30	6	30223398	chr6_30223398	+	ENSOARG00020016727	TSPAN5
31	1	80513972	chr1_80513972	+	ENSOARG00020001545	SNX7
32	5	19213882	chr5_19213882	+	ENSOARG00020020198	TLE2
33	6	30000899	chr6_30000899	+	ENSOARG00020016727	TSPAN5
34	13	43427874	chr13_43427874	+	ENSOARG00020004744	NXT1
35	12	54926382	chr12_54926382	+	ENSOARG00020009714	AURKAIP1
36	6	29409839	chr6_29409839	+	ENSOARG00020015568	ADH4
37	7	36309281	chr7_36309281	+	ENSOARG00020012944	GCHFR
38	6	29975188	chr6_29975188	+	ENSOARG00020016727	TSPAN5
