# Supplementary material for: Genome-wide association study reveals candidate genes for body size and reproductive traits in Hu sheep
Source: Anim Biosci. 2025 Nov 10;39(5):250716. doi: 10.5713/ab.250716 (PMC13175056; doi:10.5713/ab.250716)
Supplement: Supplementary file 7 [file ab-250716-Supplement-7.pdf]

Supplement 7. Phenotypic data

ID	sex	birth date	age at months	body weight	body height	body length	chest circumference	cannon bone circumference	age at lambing (in months)	lambing date	litter size	teat number
A179079	ewe	2017/2/26	87	56.3	73	74	91	8	NA	NA	NA	2
A178150	ewe	2017/5/9	85	56.1	69	75.5	96	7.8	68	2023/1/18	2	2
B189228	ewe	2018/9/8	69	53.1	71.2	79	91.4	7.3	52	2023/1/20	3	2
B189221	ewe	2018/9/5	69	54.6	75.8	86.2	100.1	8	52	2023/1/10	2	2
B189284	ewe	2018/9/23	69	53	70	71	90	8.2	51	2023/1/8	3	2
B179211	ewe	2017/9/2	81	65	72	80.5	98	7.5	64	2023/1/6	1	2
A198220	ewe	2019/5/27	60	53.7	70.3	83.1	96	7.2	43	2023/1/12	4	2
B219002	ewe	2021/1/2	41	56.1	73.2	82.7	90.5	7.3	24	2023/1/24	2	2
B189055	ewe	2018/1/20	77	53.1	71.2	78.5	92.1	7.7	60	2023/1/20	2	2
A167136	ewe	2016/9/2	93	66.7	75	85	102	9	NA	NA	NA	4
A209061	ewe	2020/2/11	52	61.6	77.8	82.5	96	8	35	2023/1/13	2	2
G198161	ewe	2019/4/18	62	61.4	73.5	88.1	103	9.1	46	2023/2/22	3	2
G178060	ewe	2017/2/1	88	61.5	73	78.5	89	NA	NA	NA	NA	2
G177092	ewe	2017/6/11	84	56.1	71.5	79	87.6	NA	68	2023/3/10	3	4
A198238	ewe	2018/9/12	69	53.8	71.6	79.5	92.4	NA	55	2023/5/8	3	2
A189260	ewe	2020/4/10	50	55.2	72	81.9	91.7	NA	37	2023/5/31	2	2
M186240	ewe	2017/5/26	84	54.5	75.5	82.1	93.6	7.9	71	2023/5/7	3	2
M209098	ewe	2018/1/20	77	53	70.6	78.9	91.7	7.5	63	2023/5/16	3	4
A178153	ewe	2020/5/12	49	53.7	69.8	78.3	87.5	7.8	36	2023/6/6	2	4
A219042	ewe	2020/3/1	51	52.8	73	79	88.3	7.5	38	2023/5/4	3	3
A189053	ewe	2021/2/2	40	53.2	71.6	81.6	90.1	7.6	27	2023/5/6	3	3
A207067	ewe	2021/9/10	33	53.1	73	81.7	90.1	7.6	19	2023/5/8	3	2
M209070	ewe	2017/1/29	88	57.8	73.8	82.1	93	7.6	76	2023/6/15	2	2
A219032	ewe	2021/10/10	32	54.7	72	83	86.7	7.2	19	2023/6/1	3	2
A219337	ewe	2019/9/13	57	45.9	70.2	71.3	85.6	7	44	2023/6/11	1	2
G178025	ewe	2022/1/11	29	59.3	75.1	77.2	82.3	7.5	11	2023/1/10	1	2
G218465	ewe	2022/1/21	29	50.6	70.3	75.4	85.2	7.2	19	2023/9/16	3	2
G196346	ewe	2018/12/21	66	46.3	73.3	79.4	87.5	7	53	2023/6/12	3	4
G199402	ewe	2017/1/22	89	54.3	75.2	85.7	95.4	7.7	76	2023/6/10	3	2
G199565	ewe	2020/3/29	50	52.5	76.5	78.9	88.7	7.4	38	2023/6/9	3	4
M228046	ewe	2020/3/2	51	44.7	70.2	76.4	79.3	7	39	2023/6/11	3	2
M228089	ewe	2021/2/7	40	48.8	69.6	74.4	84.5	7	28	2023/6/9	3	2
M186279	ewe	2019/1/16	65	52.2	74	79.4	89.1	7.6	52	2023/6/7	2	2
A189403	ewe	2020/9/12	45	53.3	75	79.5	91.2	7.6	32	2023/5/14	2	2
B179007	ewe	2017/4/1	86	59.3	73.5	80.4	95.4	7.4	74	2023/6/7	4	2
A209082	ewe	2017/5/31	84	53.4	73.2	78.9	90.1	7.2	NA	NA	NA	2
M209073	ewe	2019/1/7	65	49.5	69.8	75.1	80.4	7.2	52	2023/5/21	3	2
B217071	ewe	2021/10/23	32	56.8	71.2	76.1	90.2	7.5	18	2023/5/15	2	2
M198012	ewe	2021/2/8	40	47	68.1	71.7	80.8	7.2	27	2023/5/16	2	2
G208225	ewe	2021/1/26	40	51	71	76.5	87.6	7.3	27	2023/5/17	3	3

A178128	ewe	2021/9/19	33	60.3	71.5	81.4	97.6	7.5	19	2023/5/15	2	2
A178155	ewe	2021/9/24	33	55.2	71.2	80.3	90.2	7.9	NA	NA	NA	2
A219397	ewe	2021/9/28	32	49.6	68.6	77.4	85.3	7	19	2023/5/11	1	2
B196009	ewe	2021/1/25	41	50.4	71.8	79.5	82.7	7.3	27	2023/5/15	3	2
B217437	ewe	2021/9/10	33	50.1	71.1	75.2	90.1	7	20	2023/5/11	1	2
B217076	ewe	2019/8/31	57	45.5	70.5	77.6	85	7.2	44	2023/5/8	1	2
B219009	ewe	2017/1/21	89	52.5	68.4	81	84	7	79	2023/9/15	2	2
A219372	ewe	2021/9/30	32	47.5	67.6	76.3	83.7	7.1	19	2023/5/13	3	2
A219392	ewe	2018/9/1	69	51.5	67.8	77.2	86.3	7.2	56	2023/5/10	2	2
M218402	ewe	2019/9/18	57	44.2	68.2	76.4	84	7	43	2023/5/11	3	2
M218434	ewe	2019/8/29	57	52.7	69.8	77.4	86.2	7.3	44	2023/5/14	4	2
B217033	ewe	2018/9/24	69	53.6	70.6	79	84.5	7.5	NA	NA	NA	2
M186069	ewe	2021/9/9	33	53.1	72.5	81.2	88.4	7.3	20	2023/5/10	1	2
A219339	ewe	2021/10/23	32	47.1	71.2	77.4	85.3	7	18	2023/5/14	2	2
A198287	ewe	2021/1/28	40	51.6	72.6	82.4	93.7	7.2	27	2023/5/8	2	2
A179037	ewe	2020/8/27	45	46.7	66.2	74.2	88.3	7.5	NA	NA	NA	2
M218442	ewe	2021/9/28	32	51.6	70	78.2	84.3	7.4	16	2023/2/24	3	2
A189248	ewe	2016/9/7	93	51.3	71	79.5	88.6	7.4	77	2023/2/10	3	2
M198331	ewe	2019/1/11	65	47.6	72.8	75.9	88.6	7	48	2023/1/23	2	2
A198281	ewe	2018/1/21	77	48.7	71.9	80.6	93.4	7	59	2023/1/6	3	4
M186355	ewe	2019/5/9	61	49.9	72	75.6	89.4	7.3	44	2023/1/16	4	4
A219333	ewe	2017/5/2	85	48.1	67.4	75.6	83.8	7	68	2023/1/20	2	2
B217435	ewe	2021/11/15	31	46.2	66.5	74.2	82.3	7	22	2023/9/25	3	2
G218012	ewe	2021/6/25	36	64.6	73.2	80.6	93	8	26	2023/9/20	2	2
G208152	ewe	2021/6/20	36	53.4	72.5	80.8	90	7.6	27	2023/9/22	3	2
M218437	ewe	2020/9/7	45	49.2	73	81	83	7.2	28	2023/1/10	2	2
M169188	ewe	2019/4/23	62	43	70	73	88	7.5	44	2023/1/16	3	2
M198006	ewe	2019/12/13	54	51.8	70.6	79.5	94.1	7.5	45	2023/9/25	2	2
A189057	ewe	2020/9/10	45	58.2	72.6	83	103	8	28	2023/1/20	2	4
A198210	ewe	2020/10/4	44	57.1	75.4	84.2	99.4	8	27	2023/1/8	2	2
A177065	ewe	2021/5/1	37	47.6	71.5	72	87	8.2	NA	NA	NA	2
B217456	ewe	2021/5/19	37	55.2	74	87.4	84	8.1	NA	NA	NA	2
M218348	ewe	2021/5/10	37	50.1	71	65	85.3	7	NA	NA	NA	2
M218341	ewe	2021/4/21	38	51.3	82	72	87.1	7.3	NA	NA	NA	2
E208202	ewe	2021/5/12	37	50.7	69.1	76.5	83.4	7.4	NA	NA	NA	2
G198175	ewe	2021/4/30	37	56.2	75.6	84.2	95.8	7.8	NA	NA	NA	2
G199597	ewe	2021/5/13	37	53	73.9	82.1	89.5	7.4	NA	NA	NA	2
E208215	ewe	2021/5/27	36	54.2	71.5	82	94.2	8.1	NA	NA	NA	2
E208290	ewe	2021/5/18	37	42.9	67.8	77.1	81	6.5	24	2023/5/21	2	4
B217175	ewe	2021/7/7	35	47.7	70.1	76.5	81.6	6.9	25	2023/8/22	3	2
C2110261	ewe	2021/5/21	37	43.6	66.3	72	84	7	NA	NA	NA	4
B218224	ewe	2021/5/9	37	49.8	67.3	78	84.2	7.1	NA	NA	NA	2
C2110149	ewe	2021/4/14	38	48.6	70.3	76.1	84.2	6.8	NA	NA	NA	2

B217266	ewe	2021/12/27	29	48.4	67.7	75.5	87.2	6.8	NA	NA	NA	2
B217169	ewe	2021/5/18	37	49.2	69	75.5	84.2	7	24	2023/5/25	3	2
B217281	ewe	2021/5/3	37	46.6	67.5	74	80.2	6.9	NA	NA	NA	2
A219208	ewe	2022/3/4	27	47.3	72	75	90	7	12	2023/3/17	1	4
K2110257	ewe	2022/3/14	27	50.5	73	78.2	87.1	7.2	12	2023/4/1	2	2
A219285	ewe	2022/2/6	28	47.2	73.1	75.6	78.9	6.9	13	2023/3/23	2	2
K2110277	ewe	2022/2/6	28	48.5	72	76.2	83.8	7	13	2023/3/22	1	2
K2110195	ewe	2022/2/2	28	49.2	68.5	78.1	85	7	NA	NA	NA	2
F218097	ewe	2021/5/7	37	45.5	67.1	73	79.8	6.8	24	2023/5/17	2	2
F217477	ewe	2021/5/22	37	52	70	79	92.8	7.5	23	2023/5/5	3	2
E219178	ewe	2021/7/9	35	45.8	64.8	75.4	80.6	6.8	NA	NA	NA	2
E217195	ewe	2021/5/18	37	44.1	64.6	72.5	75.8	6.8	23	2023/5/5	3	2
A229119	ewe	2021/2/19	40	48.2	67.1	76	80.3	7.2	NA	NA	NA	2
A229138	ewe	2021/5/7	37	47	70.2	74.1	79.6	7.6	24	2023/6/3	2	2
A229095	ewe	2021/5/25	37	56.3	69	76.6	83.6	7	NA	NA	NA	2
F227074	ewe	2021/6/6	36	50.2	69.2	76.2	86.2	7.2	NA	NA	NA	2
M228149	ewe	2021/5/16	37	50	69.5	80.4	80.8	7.5	20	2023/2/1	2	2
A217230	ewe	2021/6/10	36	50.2	70	78.5	86.1	7.6	27	2023/9/23	2	2
M218255	ewe	2018/9/5	69	46.7	67.8	74	82.7	6.9	52	2023/1/18	3	2
M218197	ewe	2020/9/26	44	49.1	69.5	76.2	83.8	6.8	29	2023/3/21	2	2
I219299	ewe	2016/9/22	93	47	67.9	74.5	84.1	6.9	75	2023/1/16	2	3
M218247	ewe	2018/9/4	69	50.1	69.9	77.1	85.2	7.2	52	2023/1/13	2	2
D2110123	ewe	2020/1/8	53	51	69.5	75.7	97	7.2	36	2023/1/12	3	4
M218182	ewe	2021/1/9	41	50.2	69.6	77.4	85.4	7.5	29	2023/6/16	3	2
F217221	ewe	2019/4/27	61	46.8	67.5	73.9	81.4	6.9	45	2023/1/29	4	2
M218266	ewe	2021/2/10	40	49.3	70.7	76.7	84.5	7	26	2023/5/5	2	2
M218299	ewe	2019/1/23	65	48.1	67.8	75.9	84.1	7	47	2023/1/18	2	4
C2110244	ewe	2021/2/3	40	49.3	69.2	77	86.1	7.1	27	2023/5/5	2	2
K2110400	ewe	2021/2/16	40	46.2	67.5	74.6	80.9	7	26	2023/5/10	3	4
K186261	ewe	2019/5/2	61	49.6	70.1	75.6	91.3	7.5	44	2023/1/12	3	2
K208276	ewe	2019/4/27	61	48.7	68.6	73.4	87	7	45	2023/1/27	3	2
K169200	ewe	2018/3/2	75	45.5	71.5	75	91	7.1	NA	NA	NA	2
K186252	ewe	2019/1/17	65	50.5	71.2	78.1	87.5	7	47	2023/1/5	3	2
D209024	ewe	2020/10/2	44	57.4	79	81.6	95.1	8.5	35	2023/9/24	3	2
F217021	ewe	2021/1/10	41	50.5	70.7	80.2	88	7.4	24	2023/1/11	3	2
D196153	ewe	2020/9/1	45	60.5	78.4	89.2	98.1	8.5	28	2023/1/13	1	2
F217080	ewe	2021/1/10	41	49.1	69.8	82.3	87.6	7.8	23	2023/1/3	1	4
D199022	ewe	2019/5/1	61	49.1	68	76.3	82.8	7	44	2023/1/15	3	2
F217061	ewe	2019/7/4	59	52	68.8	74.6	81.4	7	42	2023/1/8	3	2
F217102	ewe	2019/9/10	57	51.3	70.5	75.9	86.2	7.2	40	2023/2/4	2	2
D196181	ewe	2021/1/4	41	53.2	70.4	81	88.7	7.4	24	2023/1/12	1	2
F198186	ewe	2020/10/5	44	54.1	69.6	81.9	93.2	7.5	29	2023/3/14	3	2
D188169	ewe	2020/10/1	44	53.3	72	81.4	90.2	7.8	35	2023/9/24	2	4

F198017	ewe	2019/11/27	54	47.8	67.8	76.1	77.9	7	46	2023/9/27	2	3
D2010420	ewe	2019/12/12	54	55.2	68.2	76.5	91.6	7.5	45	2023/9/24	3	2
D2110010	ewe	2020/2/25	52	50.1	68	80.2	87.6	7.5	36	2023/3/13	1	2
F209235	ewe	2020/9/15	45	51.7	68.5	76	84.1	7	29	2023/2/27	2	2
D2110012	ewe	2020/9/10	45	52.5	71.9	78.5	89.5	7.5	30	2023/3/17	2	2
F198198	ewe	2018/2/15	76	54.1	71.2	79.7	92	7.5	NA	NA	NA	4
D199344	ewe	2018/9/17	69	53	72.4	80.9	91.8	8	55	2023/5/10	2	2
D199394	ewe	2021/10/2	32	49.4	70.1	82	92.5	7.3	20	2023/6/13	2	2
D2110002	ewe	2016/9/3	93	51.5	70.8	74.9	93.2	7.5	80	2023/5/16	2	2
D2010433	ewe	2016/9/7	93	57	72	78.9	91.3	7.3	80	2023/5/9	2	2
D2010405	ewe	2020/3/29	50	56.5	73.6	76.4	90.1	7.6	38	2023/5/31	2	2
D199559	ewe	2018/11/1	67	50.6	70.8	79.6	82.8	7.5	58	2023/9/19	2	4
F198485	ewe	2019/10/10	56	50.8	70.3	79.2	80.6	8	43	2023/6/7	3	2
K2011023	ewe	2022/1/13	29	49.8	71.5	78	82.6	7.1	11	2023/1/5	2	4
F209291	ewe	2022/1/5	29	50.9	72	75.9	87.1	7.5	20	2023/9/29	1	2
K208218	ewe	2020/4/7	50	53.8	73	81.9	92.1	8.1	37	2023/5/25	4	2
F188133	ewe	2021/9/13	33	51.6	71	80.2	88.7	7.5	20	2023/6/9	2	2
F186335	ewe	2020/3/31	50	51.6	71	76.9	85.7	7.3	39	2023/7/11	3	3
F217386	ewe	2017/2/27	87	47.6	66.7	74.2	84.6	7	74	2023/5/5	4	3
F168141	ewe	2019/9/1	57	52.5	68.7	80.3	92.3	8.5	44	2023/5/23	3	4
F167091	ewe	2021/1/27	40	53.4	68.5	77.2	85.3	7.1	27	2023/5/8	2	2
D2010108	ewe	2020/5/20	49	49.5	70.6	80.1	82.4	8.2	35	2023/5/8	3	4
D188374	ewe	2019/11/7	55	52	69.5	78.6	89	7.3	42	2023/5/7	2	2
F199422	ewe	2018/12/27	65	50.7	75.9	81.1	89.1	7.2	52	2023/4/30	4	2
F227047	ewe	2020/3/26	50	52	70.3	75.4	88.2	7.2	37	2023/5/20	2	2
D2210020	ewe	2020/3/26	50	46.5	66.5	75	85.8	7	37	2023/5/17	1	2
F2010120	ewe	2019/11/8	55	48.9	70.1	75.2	79.8	7.2	42	2023/5/10	2	4
D2110466	ewe	2020/4/23	50	48.7	66	87.8	78	7.1	37	2023/6/18	1	2
F198388	ewe	2021/9/20	33	53.8	77.4	84	96.5	7.8	19	2023/5/13	3	2
D199009	ewe	2017/4/25	86	53	72.8	79.5	90.2	7.5	72	2023/5/20	1	4
D2010113	ewe	2017/9/20	81	51	69.2	76.5	87.4	7.1	68	2023/6/14	4	2
D178077	ewe	2020/4/14	50	46.3	68.5	78.2	83.1	7.1	36	2023/5/7	2	4
D199375	ewe	2020/4/29	49	60.5	78.1	89	99.6	7.9	36	2023/5/22	2	4
D2110020	ewe	2019/7/20	59	44.8	68.4	72.5	79.6	6.9	45	2023/4/29	4	2
D207073	ewe	2020/5/12	49	58.6	75.6	83.2	91.4	7.9	35	2023/5/4	3	4
D199456	ewe	2019/11/11	55	45.6	70.8	75.5	81.4	7.6	NA	NA	NA	2
D2110004	ewe	2018/2/21	76	45.4	69.5	74.1	79.2	6.8	NA	NA	NA	4
D188490	ewe	2021/9/10	33	50.2	72.1	79	84.4	7.2	20	2023/6/5	4	4
F207001	ewe	2020/4/8	50	48.2	69.6	74.5	83	7.6	37	2023/5/11	3	2
F217350	ewe	2017/2/11	88	46.5	76.2	71	87	7	74	2023/5/3	2	2
F198360	ewe	2019/1/18	65	53.6	75.3	85.2	95.8	7.4	51	2023/5/1	4	2
F207052	ewe	2018/9/18	69	52.7	70.2	76.8	90	7.8	55	2023/5/10	2	2
F188081	ewe	2019/10/29	55	48.9	71	77.6	82.7	7.6	46	2023/9/14	3	2

F217354	ewe	2021/7/1	35	55	85.6	72	91.3	7.5	18	2023/1/28	2	2
F178132	ewe	2020/1/14	53	52.4	72.1	77.3	95.2	7.5	35	2023/1/8	3	2
F179196	ewe	2020/9/20	45	53.2	70.5	78.1	90.3	7.4	35	2023/9/10	2	2
F209108	ewe	2019/9/12	57	50.7	69.7	78.8	84.5	7.2	40	2023/1/17	3	3
K2010146	ewe	2020/1/12	53	49.5	69.5	78.6	81.4	7.4	36	2023/1/14	3	2
F198269	ewe	2021/3/27	38	52.4	74.6	84.7	96.2	7.6	21	2023/1/9	1	2
F207064	ewe	2020/4/1	50	51.5	76	77.9	85.8	8.3	34	2023/2/16	4	2
K199467	ewe	2020/9/14	45	48.7	70.1	79.8	89.7	7.2	36	2023/9/17	3	NA
F188150	ewe	2019/8/22	58	49.7	70.4	78.5	86.8	7	42	2023/2/27	2	2
K2110453	ewe	2017/3/9	87	48.1	64.7	74.2	83.6	6.9	72	2023/4/1	2	4
K2010122	ewe	2019/11/7	55	53.2	71.5	81	90.2	7.6	37	2023/1/6	2	2
D178054	ewe	2019/8/29	57	58.3	71.5	80.1	86.4	7.1	40	2023/1/20	3	2
D199012	ewe	2019/6/13	60	50.9	70.6	77.7	87.4	8	42	2023/1/5	2	2
F188281	ewe	2020/2/20	52	58.2	71.5	81.5	90.6	8	42	2023/9/13	2	2
C198357	ewe	2021/6/23	36	55.5	76.3	82.4	98.1	7.8	18	2023/1/6	2	4
C2110421	ewe	2020/4/9	50	47.4	68.3	72.5	87	6.9	35	2023/3/19	3	2
C2010046	ewe	2021/6/27	35	65.2	74.2	81.4	102.3	9	18	2023/1/4	3	2
C2010379	ewe	2021/6/21	36	52.7	70.5	79.5	90.5	7.6	18	2023/1/20	1	2
I196290	ewe	2017/2/25	88	55.2	75.2	83.6	97.6	7.6	72	2023/3/10	3	2
C209035	ewe	2021/7/1	35	49	71	76.9	90.5	7.2	18	2023/1/6	2	2
C2110148	ewe	2019/11/18	55	53	80	70	93	7.3	42	2023/6/7	3	3
E207014	ewe	2019/9/7	57	55.4	77.1	83	98.2	9	40	2023/1/8	1	2
C2010361	ewe	2018/11/5	67	49.8	67.9	75.7	88.4	8	55	2023/6/9	3	4
E199351	ewe	2019/8/25	58	51.3	70.9	81.3	95	7.3	40	2023/1/2	4	4
E179119	ewe	2021/10/7	32	47.5	70	73	84	7.2	20	2023/6/7	3	4
I196336	ewe	2020/4/22	50	53.9	76	83.4	96.3	7.7	37	2023/6/10	4	2
I196255	ewe	2018/10/31	67	57.8	75.6	87.1	100.6	7.8	NA	NA	NA	2
C199315	ewe	2020/9/27	44	49.4	65.8	76.2	87.4	7	29	2023/3/16	3	2
C2010089	ewe	2021/1/31	40	47.2	69	75.2	81.4	7	25	2023/3/12	3	2
I219254	ewe	2019/11/22	55	45.3	67.3	69.4	81.6	6.9	39	2023/3/15	1	2
C2010125	ewe	2021/9/24	33	53.5	71.5	78.8	83.4	7.3	19	2023/5/7	4	2
C2110413	ewe	2017/9/1	81	40.8	62	67.6	82	6.8	66	2023/3/15	3	2
I219242	ewe	2021/9/6	33	45.7	67.1	71.2	82.5	7	20	2023/5/19	3	2
I178083	ewe	2019/8/28	57	53.8	71.2	77.5	89.5	8.1	45	2023/6/23	3	3
C2110415	ewe	2017/5/4	85	41.4	63	76	82	6.8	72	2023/5/14	4	2
E196353	ewe	2020/4/20	50	48.5	71.4	80.7	93.6	7.2	37	2023/5/23	1	2
I196279	ewe	2018/1/25	77	56.4	76	85.4	98.4	7.8	59	2023/1/22	1	2
E188388	ewe	2018/2/12	76	53.1	71.2	81.1	90.6	7.6	63	2023/6/4	4	2
E196230	ewe	2021/1/31	40	56.2	72.3	78.1	93.4	7.2	27	2023/5/7	2	2
E217403	ewe	2021/2/4	40	45.8	65.4	73.2	81.6	7	27	2023/5/12	3	2
E207047	ewe	2018/1/12	77	49	66.8	75.2	81.6	6.9	63	2023/5/11	2	4
C186388	ewe	2018/9/21	69	51.2	69.3	76.6	90.2	7.2	55	2023/5/14	3	4
I2010397	ewe	2018/10/31	67	49	68.7	73.5	87.9	7.1	55	2023/6/9	2	4

C2110039	ewe	2022/1/9	29	46.2	68.4	75	85.1	6.9	12	2023/1/16	1	2
I199524	ewe	2021/2/2	40	48.3	72.1	81.5	91.2	7.1	27	2023/5/20	2	2
C2110491	ewe	2016/10/24	92	47.1	69	72.6	85	7	79	2023/6/4	2	2
C178223	ewe	2016/9/3	93	42.4	72.1	74.2	86.4	7.3	NA	NA	NA	2
C2110446	ewe	2021/6/26	35	51.2	73	79.3	88	7.5	23	2023/6/15	1	2
I196245	ewe	2019/11/4	55	49.7	70.2	82.3	95.5	7.4	42	2023/5/16	3	2
P69-210	ewe	2021/1/23	41	50.6	71.2	77.5	92.1	7.6	27	2023/5/16	3	2
C2010136	ewe	2022/1/22	29	51.4	70	79.2	88.7	7.4	19	2023/9/18	2	2
I188074	ewe	2021/10/13	32	53.4	70.5	80.6	88.8	7.5	19	2023/6/1	3	4
C189110	ewe	2021/10/9	32	50.6	71	77.4	90.5	7.7	19	2023/5/23	2	2
I219025	ewe	2021/2/15	40	47	70.5	75.1	81.4	6.9	27	2023/6/10	3	4
C2110070	ewe	2019/8/28	57	50.3	71.5	74.2	84.2	7.2	45	2023/6/2	2	2
I188034	ewe	2019/11/4	55	51.7	71.6	81	85.5	7.8	42	2023/5/16	1	2
C189280	ewe	2019/6/17	60	51.8	71.5	78.6	88.4	7.7	46	2023/5/14	4	4
C188369	ewe	2021/9/16	33	52	75	78.6	86.4	7.4	20	2023/5/18	3	4
I229018	ewe	2020/4/29	49	38.9	65.4	71.8	77.2	6.9	36	2023/5/20	3	2
C2110056	ewe	2020/4/8	50	46.2	70.6	73.1	81.7	6.9	37	2023/5/18	3	2
C196235	ewe	2020/8/26	45	50.7	77.3	80.1	87.2	7.4	31	2023/4/25	3	2
C168145	ewe	2018/1/3	77	49.3	67.5	76.2	89.1	7.3	65	2023/6/27	2	2
C168196	ewe	2020/4/7	50	48.5	72.1	73.5	87.3	7.7	37	2023/5/8	3	NA
I219271	ewe	2019/12/3	54	49.3	69.1	76.6	84.2	7	41	2023/5/12	3	2
C199439	ewe	2018/10/27	67	51.2	75.8	83.2	94.2	7.5	54	2023/5/4	3	2
C2110016	ewe	2020/8/24	46	45.8	67.5	74.2	80	6.9	32	2023/5/6	3	2
I229052	ewe	2020/4/9	50	51.3	68	73.5	87.6	7.2	37	2023/5/18	2	2
I219400	ewe	2022/1/23	29	47.6	66.2	74.9	84.3	6.8	11	2023/1/5	3	2
C2110535	ewe	2022/1/10	29	45.6	66.6	74.2	85	6.8	11	2023/1/4	1	2
I219066	ewe	2021/9/18	33	46.6	66	72.8	82.1	6.9	19	2023/5/11	2	3
E199364	ewe	2021/9/16	33	54.6	73.5	87	91.8	7.6	20	2023/5/16	2	2
E196329	ewe	2021/6/25	36	47.3	69.1	78.4	90.8	7.1	18	2023/1/4	2	2
E199324	ewe	2022/1/20	29	51	73.5	80.6	84.8	7.7	14	2023/3/27	2	2
E217336	ewe	2019/9/16	57	47.9	67.4	75	83.1	6.9	48	2023/9/30	3	2
E207058	ewe	2020/7/15	47	50.9	73.8	78.7	89.2	7.4	34	2023/6/1	3	2
E207031	ewe	2020/6/19	48	51	73.5	80.6	84.8	7.7	35	2023/5/20	3	2
E208144	ewe	2017/9/8	81	57.5	74.2	83.6	89	8	68	2023/5/15	3	2
E188006	ewe	2020/8/28	45	52.7	70.8	81.1	89.5	7.5	32	2023/5/16	2	2
E2010117	ewe	2020/8/28	45	50.4	71	77.7	83.9	7.2	32	2023/5/19	1	2
E196404	ewe	2019/6/18	60	53.8	71.5	81.7	92.1	7.8	NA	NA	NA	4
E188365	ewe	2017/10/1	80	53.1	69.5	81.9	86.7	7.5	67	2023/5/11	3	2
E217016	ewe	2017/2/6	88	47	68.1	74.9	82.4	7	NA	NA	NA	2
E208142	ewe	2021/4/21	38	49	69.1	75.5	82.4	6.8	24	2023/5/8	3	4
E207034	ewe	2017/2/9	88	50	71	77.5	79.8	6.6	75	2023/5/12	2	2
D2210149	ewe	2018/1/27	76	49.5	68.2	74	87.4	6.9	63	2023/5/16	2	2
G228029	ewe	2020/9/6	45	42.5	68.7	73.5	82.5	6.9	32	2023/5/17	3	2

E217342	ewe	2021/2/18	40	47.3	70.9	80.3	85	7.5	26	2023/5/10	3	2
E217339	ewe	2019/1/28	64	46.2	69.5	79.2	86.7	7.4	51	2023/5/11	3	4
C2110410	ewe	2020/6/26	47	45.5	68	80	85	6.7	34	2023/5/12	2	2
C2210117	ewe	2018/6/10	72	53.2	69.5	81.3	82.9	7.5	59	2023/5/20	1	2
E199411	ewe	2017/2/1	88	54	73.5	86.2	94.9	8.9	75	2023/5/4	3	2
E207171	ewe	2017/1/21	89	49.7	69.4	76.9	84.3	7.2	75	2023/5/4	2	3
K2010198	ewe	2021/2/6	40	49.3	71.1	74	86.7	7.7	27	2023/5/15	3	2
D179194	ewe	2020/10/3	44	45.3	70.1	78.5	84.2	7.1	NA	NA	NA	2
D2010276	ram	2021/6/18	36	49.1	69.2	75.5	79.8	7.5	NA	NA	NA	2
D2010274	ram	2021/6/7	36	50.2	67.4	76.7	87.2	7.3	NA	NA	NA	2
D199327	ram	2021/10/23	32	48.1	69.2	75.5	81.7	6.9	NA	NA	NA	2
E178229	ram	2021/6/21	36	57.4	72.4	78.2	88.5	7.5	NA	NA	NA	2
G178040	ram	2021/6/25	36	49.6	70.2	78.4	86	7	NA	NA	NA	2
E218130	ram	2021/10/1	32	48.5	71	79.1	85.1	8.2	NA	NA	NA	4
K177009	ram	2021/1/2	41	67.2	69.3	82.1	92.1	8.1	NA	NA	NA	2
E188086	ram	2021/6/25	36	50.2	70.4	73.2	84.3	7.1	NA	NA	NA	2
D2010311	ram	2021/5/1	37	51	69.7	79.6	88.2	7.4	NA	NA	NA	2
D2110116	ram	2022/1/21	29	48.5	72.5	76.7	82.6	7.2	NA	NA	NA	2
D199032	ram	2022/1/24	29	50.1	67.5	78.2	85.7	7.4	NA	NA	NA	2
E2010213	ram	2021/6/17	36	51.5	71.6	79.8	90.5	7.4	NA	NA	NA	4
A189142	ram	2022/1/6	29	54.5	71.5	82.6	88.1	7.5	NA	NA	NA	2
D178045	ram	2022/1/3	29	61.2	71.5	81.2	90.5	7.6	NA	NA	NA	4
A179030	ram	2022/1/9	29	50.8	70.6	76.6	86.1	7.3	NA	NA	NA	4
D2110096	ram	2021/10/17	32	48.8	68.5	77.1	85.2	7	NA	NA	NA	2
M209318	ram	2021/1/4	41	56.6	70.5	77.6	87.5	7.6	NA	NA	NA	2
M218338	ram	2022/1/7	29	61.7	74	79.5	89.5	7.7	NA	NA	NA	2
G218307	ram	2022/1/6	29	59.5	74.6	79.7	90.4	8.1	NA	NA	NA	NA
F217439	ram	2022/1/11	29	63.5	79.2	85.5	89.1	8.4	NA	NA	NA	NA
A219240	ewe	2021/2/2	40	59.4	74.6	77.5	89.3	8.2	28	2023/6/17	2	NA
I219265	ewe	2021/10/13	32	63.2	75.3	82.5	94.1	7.6	19	2023/5/17	2	NA
E217384	ewe	2021/1/6	41	50.6	75.2	81.7	81.3	7.8	28	2023/5/16	2	NA
B219001	ewe	2018/1/21	77	68.4	78.5	86.5	97.1	8.2	63	2023/5/14	1	NA
C2110409	ewe	2021/10/4	32	48.6	73.2	77.3	87	7.8	19	2023/5/17	2	NA
K2110157	ewe	2021/10/17	32	60.4	75.8	80.3	92.2	8	19	2023/6/8	2	NA
K2210133	ewe	2021/10/1	32	62.5	71.2	80	94.4	7.1	20	2023/6/6	2	NA
A229058	ewe	2018/3/25	75	61.5	76.5	80.3	95.3	8.1	61	2023/5/15	3	NA
I219228	ewe	2021/10/1	32	65.2	72	81.6	94.5	8.3	19	2023/5/21	3	NA
E227023	ewe	2022/1/6	29	56.4	77.4	80.5	93.2	8	11	2023/1/4	2	NA
C2210009	ewe	2021/1/28	40	52.4	74.6	84.7	92.8	8	28	2023/6/16	1	NA
M228027	ram	2020/1/14	53	55.6	72.7	81.6	92.6	8	NA	NA	NA	NA
B217416	ewe	2021/10/11	32	52.3	73.5	80.2	84.2	8.5	NA	NA	NA	NA
D2110001	ewe	2022/01/09	29	73.4	86.8	86.8	98.5	8.2	14	2023/3/30	2	NA
D2210023	ewe	2022/01/14	29	54.6	75.5	80.3	93.1	7.4	14	2023/3/21	2	NA

F227031	ewe	2022/01/02	29	56.1	73.8	78.2	90.4	7	15	2023/4/5	3	NA
G228035	ewe	2022/01/17	29	70.4	76.6	84.1	93.2	8.1	12	2023/2/11	2	NA
C2110051	ram	2021/9/5	33	47.3	75	66.5	85.4	6.4	NA	NA	NA	2
I219402	ewe	2019/01/15	65	36.8	73.5	66.5	79.2	6.3	NA	NA	NA	2
C189061	ewe	2020/2/15	52	53.6	81.4	72.6	90.5	7.7	37	2023/3/15	2	2
C2110524	ewe	2020/03/26	50	38.1	78.7	70.5	83.4	7.6	38	2023/5/26	1	2
I219407	ewe	2022/01/18	29	41.2	67.5	67.8	89.3	7.5	11	2023/1/7	2	2
E217385	ewe	2022/01/15	29	38.8	70.2	67.8	87.7	7.5	11	2023/1/4	2	2
I186108	ewe	2020/10/04	44	48.9	74.8	70	86.5	7	27	2023/1/8	4	2
C2110508	ram	2019/3/30	62	36.9	72.4	68.7	87.8	7.4	NA	NA	NA	4
E227024	ewe	2018/12/25	66	36.7	73.6	69.2	93.5	7.3	52	2023/5/6	4	4
I219015	ewe	2019/9/14	57	48	73.5	69.1	76.9	6.3	43	2023/5/2	3	2
F2010042	ewe	2020/04/07	50	NA	NA	79.2	101.3	8.7	37	2023/5/25	4	NA
D2110544	ewe	2018/12/6	66	48.8	75.6	71.3	95.2	7.1	52	2023/5/4	3	4
D2210033	ewe	2018/12/25	66	43.5	70.1	64.5	83.4	6.6	52	2023/5/15	2	2
D2210073	ewe	2018/9/11	69	44.9	70.5	62.4	85.1	7.3	56	2023/5/12	4	3
D2210006	ram	2018/2/7	76	46.5	71.5	68.6	83.8	6.9	NA	NA	NA	4
D2210102	ewe	2021/1/6	41	42.2	70.7	68.6	86.3	7.1	27	2023/5/5	4	2
A219323	ewe	2018/10/21	68	71.8	80.8	74.3	98.8	8.4	55	2023/6/14	1	NA
D199006	ewe	2022/9/21	21	50.4	79.6	66.8	95	7	NA	NA	NA	2
K2011018	ewe	2022/9/20	21	49.6	76.1	71.4	83.4	7.4	NA	NA	NA	2
F209078	ewe	2022/10/14	20	52.8	80.2	71.5	89.6	7.5	NA	NA	NA	4
D2210105	ewe	2022/9/25	21	43.7	71.5	65.9	85.4	7.2	NA	NA	NA	2
K2210092	ewe	2022/9/26	20	46.8	72.6	67.1	88.3	7.1	NA	NA	NA	2
D2010430	ewe	2022/9/30	20	57.6	80.2	72.1	90.4	7.6	NA	NA	NA	2
I199093	ewe	2022/9/7	21	79.5	86.7	81.5	102.4	8.7	NA	NA	NA	NA
D188476	ewe	2022/9/13	21	47	74.2	68.1	80	6.7	NA	NA	NA	2
D199407	ewe	2022/9/16	21	45.7	76.9	68.3	87.2	7	NA	NA	NA	2
D2010120	ewe	2022/6/15	24	48.9	75.2	70.1	79.8	7.2	NA	NA	NA	4
D188441	ewe	2022/9/13	21	48.9	78.5	67.9	83.8	7.2	NA	NA	NA	2
F188479	ewe	2022/9/8	21	52.1	78.6	72	89.8	7.4	NA	NA	NA	2
K186303	ewe	2022/6/12	24	48.8	77.2	69	82.7	7.3	NA	NA	NA	2
G188109	ewe	2022/9/11	21	73.6	84.2	80.3	100.7	8.7	NA	NA	NA	NA
F188346	ewe	2019/11/5	55	50	75.7	66.8	87	7.2	43	2023/6/7	2	2
F2210501	ram	2019/1/19	65	44.8	71.5	61.5	94.5	7.6	NA	NA	NA	2
F227335	ewe	2018/9/12	69	43.5	69.5	62.4	88.4	8.1	57	2023/6/26	3	2
F227376	ewe	2021/4/19	38	41.9	63.5	64.1	85.2	8.1	NA	NA	NA	2
K228475	ewe	2021/4/24	38	40.5	70.5	67.5	95.9	7.5	NA	NA	NA	2
K228482	ewe	2021/5/3	37	36.9	68.5	64.4	82.5	6.7	NA	NA	NA	2
K228493	ewe	2021/5/29	36	38.4	66.6	65.2	82.3	7.4	NA	NA	NA	2
F227296	ewe	2020/7/7	47	45.6	67.2	64.6	87.6	6.7	NA	NA	NA	2
K228382	ewe	2021/5/27	36	38.1	71.5	67.5	83.5	7.1	NA	NA	NA	4
K228391	ewe	2019/1/9	65	40.7	75.5	64.7	82.3	7.7	NA	NA	NA	2

F227230	ewe	2018/11/7	67	48.8	70.5	67.6	90.5	8.9	54	2023/5/18	3	2
F227327	ewe	2022/2/15	28	45.7	71.2	66.6	86	7.7	14	2023/5/1	1	2
K2210416	ewe	2021/5/6	37	41.1	73.3	65.4	87.6	8	24	2023/5/6	2	2
F227225	ewe	2019/2/20	64	44.5	73.5	62.7	82.5	7.7	50	2023/5/16	2	2
F227316	ewe	2021/5/16	37	35.2	63.5	64.1	80.1	7.9	23	2023/5/11	2	4
M199441	ewe	2021/4/27	37	48.7	78.8	68.5	93.4	8	NA	NA	NA	2
D196024	ewe	2021/4/25	38	70.1	84.5	83.4	97.2	8.2	25	2023/5/31	3	NA
M186312	ewe	2019/3/30	62	47	75.6	67.1	83.4	7.1	NA	NA	NA	2
G218123	ewe	2021/5/11	37	43.2	70.4	67.2	77.3	6.7	NA	NA	NA	2
M218144	ram	2019/2/12	64	45.2	76.1	69.3	84.2	7	NA	NA	NA	2
C2110368	ewe	2021/12/29	29	47	76	67	87	7	NA	NA	NA	2
I2010259	ewe	2021/2/17	40	49.5	77.5	67.8	82	6.9	27	2023/5/17	3	2
K2110349	ram	2019/3/28	62	43.7	72.3	69	78.1	6.8	NA	NA	NA	4
F198005	ewe	2021/5/5	37	47.4	73.5	66.5	79.7	7	24	2023/5/24	2	3
C189334	ewe	2019/3/21	63	50	77.6	67.8	80.9	7.1	49	2023/5/19	2	2
K2210168	ewe	2018/6/4	72	40.5	72.6	68.5	86.3	7.8	60	2023/6/4	2	2
B217207	ewe	2018/6/7	72	49	78	68	83	7	NA	NA	NA	2
B196058	ewe	2021/5/27	36	51.7	78.7	72.5	85	7.7	24	2023/6/4	1	2
K2110238	ewe	2019/2/6	64	50.2	76.7	79.2	84.7	7	51	2023/6/3	2	2
G218152	ewe	2018/9/2	69	45.2	75.7	71.6	80.6	7	56	2023/5/18	2	2
I219097	ewe	2021/5/4	37	43.5	75	68	80	7	NA	NA	NA	2
F198060	ewe	2019/9/9	57	49.6	73.9	69.7	85.8	7.2	44	2023/5/19	3	2
F217261	ewe	2021/5/6	37	43.8	75.9	69.2	82.1	7	NA	NA	NA	4
D199046	ewe	2021/4/26	37	72.5	83.3	80.7	96.8	9.1	NA	NA	NA	NA
F217495	ewe	2021/4/27	37	37	75.5	66.5	74.8	NA	NA	NA	NA	3
M218070	ewe	2021/5/11	37	63.5	80.7	72	92.5	7.2	NA	NA	NA	2
B198050	ewe	2018/9/8	69	76.2	84.3	77.8	97.9	8.1	55	2023/5/6	1	NA
F196067	ram	2021/6/21	36	50.4	77.6	67.9	82.1	7.3	NA	NA	NA	3
C186166	ram	2019/1/25	65	48.7	77.6	69	84.8	7	NA	NA	NA	2
M186173	ram	2021/8/26	33	50	78.4	69.5	87.9	7.4	NA	NA	NA	2
C2110344	ram	2021/9/14	33	47	76	69	80	6.5	NA	NA	NA	2
M198033	ram	2019/11/16	55	50.3	75.5	67.5	84	7.9	NA	NA	NA	4
C189202	ram	2019/4/6	62	48.2	76.7	70.5	78.8	7	NA	NA	NA	2
M218189	ewe	2022/10/24	20	43.9	73.6	69.5	79.6	6.5	NA	NA	NA	2
E196286	ewe	2022/9/28	20	52.6	81.7	68.4	88.1	8	NA	NA	NA	4
E217210	ewe	2022/10/3	20	40.5	71.5	65.6	83.5	7.2	NA	NA	NA	2
A219103	ewe	2022/9/28	20	48.2	77.4	72.5	80.4	7	NA	NA	NA	4
A219114	ewe	2019/8/30	57	45.7	74.3	67.4	76.8	6.8	45	2023/5/30	3	2
E219162	ewe	2020/10/20	44	50.4	77.2	71.7	82.9	7	31	2023/6/8	2	4
A189231	ewe	2020/7/10	47	50.1	78.7	73	82.8	7.4	34	2023/6/9	2	4
M218346	ewe	2021/4/24	38	62.7	NA	79.8	NA	NA	25	2023/6/6	2	NA
M198029	ewe	2019/7/6	59	80.7	NA	84.2	98.4	8.5	42	2023/1/15	2	NA
C2110437	ewe	2020/9/11	45	68.2	85.6	80.1	NA	NA	32	2023/5/15	2	NA

C2110469	ewe	2020/6/25	48	57.5	81.5	76.3	103.7	8.6	35	2023/6/3	2	NA
K199482	ewe	2018/6/21	72	74.6	83.5	76.2	101.2	8.2	60	2023/7/7	2	NA
K198091	ewe	2020/11/6	43	NA	86.5	79.8	102.6	8.4	31	2023/6/12	3	NA
I229449	ewe	2020/5/30	48	38.9	70.5	66.2	78.6	6.8	36	2023/6/13	3	3
I229406	ewe	2020/9/7	45	41.5	68.2	64.2	89.5	7.4	32	2023/5/18	2	2
I229423	ewe	2020/9/20	45	40.8	71.2	70.5	82.3	7.6	27	2023/1/13	1	2
I229401	ewe	2020/11/18	43	37.4	69.5	64.2	82.3	7.6	27	2023/2/27	3	2
F199367	ewe	2020/7/2	47	52.3	83.7	73.8	94.9	7.5	31	2023/2/19	1	2
D2010472	ewe	2021/5/1	37	45.5	74.2	68.9	85.1	7.3	22	2023/3/6	3	2
E207155	ewe	2022/5/10	25	52.1	81	71.5	88.6	7.5	12	2023/5/31	1	4
I219090	ewe	2018/11/15	67	42.5	72.3	70.1	74.8	6.8	54	2023/5/16	3	2
A198265	ewe	2018/2/23	76	50.7	81.3	72.2	94.6	7.5	NA	NA	NA	2
D2010339	ewe	2018/12/22	66	47.2	72.9	71	79.6	7	52	2023/5/21	3	2
E2010208	ewe	2021/5/12	37	51.4	79.9	71.5	90.1	7.5	NA	NA	NA	2
B186214	ewe	2018/9/21	69	54.1	81.5	69.2	90.1	7.6	55	2023/5/6	2	2
E208361	ewe	2018/11/17	67	51	78.6	70.8	85.5	7.7	54	2023/5/20	2	2
D2010174	ewe	2018/2/15	76	48.5	73.6	69.4	80.1	7.7	63	2023/5/18	2	4
D2010320	ewe	2018/1/9	77	46.4	70.7	66	76.1	7	64	2023/5/18	2	2
F209309	ewe	2018/11/17	67	51.4	76.1	68.5	93.1	7.4	54	2023/6/1	2	2
M209386	ewe	2018/1/28	76	55.5	81.2	72.1	88.7	7	NA	NA	NA	2
E2010231	ewe	2018/10/6	68	48.1	73.5	69.1	85.1	7.2	55	2023/5/17	1	2
E219132	ewe	2018/6/20	72	52.5	79.2	67.4	87.3	7	NA	NA	NA	2
F227116	ewe	2021/5/22	37	42.5	73.7	68.2	83.8	7.6	24	2023/6/3	2	3
C189348	ewe	2019/3/19	63	51.6	78.2	70.5	88.6	7.6	49	2023/5/6	4	2
F188152	ewe	2018/4/6	74	54.2	78.1	74	88.8	7.7	NA	NA	NA	2
F188462	ewe	2018/9/21	69	49.3	74.5	70.1	83.4	7	55	2023/5/15	2	3
F217273	ewe	2021/5/29	36	43.2	77.2	66.7	80.5	7	NA	NA	NA	4
C189279	ewe	2019/11/9	55	48.9	77.4	66.5	83.8	7	NA	NA	NA	3
C189367	ewe	2021/4/28	37	49.5	76.8	70.2	78.5	7	NA	NA	NA	2
C189117	ewe	2021/5/27	36	52.7	84	71.5	86.7	7.5	NA	NA	NA	2
M186004	ewe	2021/5/11	37	52.2	78.3	69.1	91.6	7.8	NA	NA	NA	4
C189363	ewe	2019/9/16	57	52.3	78.5	69.5	92	8.1	NA	NA	NA	3
F188092	ewe	2021/7/3	35	50.6	76.4	68.7	86.5	7.1	NA	NA	NA	4
F188317	ewe	2021/5/10	37	48.9	74.6	69.5	85.2	7.2	NA	NA	NA	3
F186205	ewe	2021/5/23	37	51.4	78.9	70	90.2	7.6	NA	NA	NA	2
M218259	ewe	2021/4/15	38	49.5	76.7	74.5	86.4	7.3	NA	NA	NA	2
F196063	ram	2019/11/25	55	50.9	78.6	69.5	83.2	7.8	NA	NA	NA	4
M186130	ewe	2018/1/27	76	61.3	85.3	69.5	101.8	7.7	63	2023/5/10	4	4
F188291	ewe	2021/9/13	33	49.3	76.6	70.1	79.8	7	19	2023/5/2	3	4
K2110372	ewe	2020/4/10	50	43.7	74.3	69.5	81	7	36	2023/5/6	3	2
M198362	ewe	2021/9/19	33	46.5	75.3	67.7	80.5	7	20	2023/6/16	2	4
B217158	ewe	2019/8/28	57	44.5	73	69	80	7.3	45	2023/6/4	2	2
C2110355	ewe	2021/9/14	33	48.5	77	70	83	7.2	20	2023/6/2	2	2

C2110207	ewe	2019/4/25	62	48	77	68	82	7	NA	NA	NA	2
K196298	ewe	2019/11/5	55	52.8	84.4	73.6	94.5	7.6	41	2023/5/1	1	2
C2110426	ewe	2018/2/23	76	38.8	74.5	69.3	89.7	7.7	NA	NA	NA	2
C2110198	ewe	2022/1/13	29	48.5	78	68	82	7	11	2023/1/4	1	2
C2110306	ewe	2018/9/19	69	48	78	NA	83	7	56	2023/5/20	3	2
F218102	ram	2019/4/29	61	46.5	77.2	NA	79.3	7.5	NA	NA	NA	2
B199545	ewe	2021/2/13	40	80.1	NA	82.5	105.6	8.8	27	2023/5/17	2	NA
F217325	ewe	2019/10/30	55	52.5	79.5	73.7	89.1	8.2	42	2023/5/13	3	2
F209103	ewe	2018/9/3	69	54.1	76.5	72.4	88.8	8.5	56	2023/5/19	3	4
D2110486	ewe	2022/1/13	29	32.8	68.6	75.6	81.5	7.2	15	2023/5/4	3	4
D196249	ewe	2018/9/3	69	54.2	86.2	74.3	96.1	7.5	57	2023/6/12	4	2
D2110472	ewe	2018/1/11	77	34.5	70.5	69.5	76.3	6.8	63	2023/5/10	3	2
F199232	ewe	2019/1/16	65	49	77.7	66.5	84.3	7	52	2023/6/8	5	4
D199444	ewe	2018/12/27	65	50.5	81.8	70.6	88.9	8	52	2023/5/5	2	4
D188153	ewe	2018/12/9	66	53	79.5	68	87.4	7.4	53	2023/5/27	2	4
F227045	ewe	2019/11/28	54	39.3	70.5	69.5	89.2	6.7	42	2023/6/12	2	3
F188296	ewe	2021/6/3	36	59.8	82.3	77.4	97	8.3	23	2023/5/17	1	4
G198194	ewe	2019/1/21	65	76.8	84.5	77.5	98.6	8.5	52	2023/5/21	3	NA
F217089	ewe	2019/9/5	57	59	76.7	67.3	87.2	7.5	44	2023/5/12	1	4
D199437	ewe	2019/1/21	65	42.7	77.5	70.2	86.4	6.8	52	2023/6/5	3	2
K186244	ewe	2018/12/18	66	52	78.5	67.9	92.1	7.5	52	2023/5/4	3	2
F227042	ewe	2020/1/4	53	32.5	76.2	64.9	80	7.8	42	2023/7/8	3	2
D188264	ewe	2019/11/17	55	45.5	72.9	68.5	76.7	6.5	NA	NA	NA	4
D188026	ewe	2019/9/24	57	46.6	76.9	67.5	78	6.7	44	2023/6/16	3	2
D188484	ewe	2021/10/12	32	47.6	74.8	70	81.6	6.9	18	2023/5/6	2	2
D188447	ewe	2021/2/24	40	47.3	75.2	69.2	78	6.8	26	2023/5/10	1	4
K199568	ewe	2021/9/21	33	51	77.8	71	88.5	7.8	19	2023/5/6	1	2
K2110390	ewe	2018/11/9	67	43.5	73.4	60.2	75.9	6.6	54	2023/5/12	3	4
F198023	ram	2021/7/3	35	49	76.7	69.1	82.6	7.2	NA	NA	NA	2
F199386	ewe	2018/11/5	67	51.2	82.3	74.1	95.2	7.4	54	2023/5/8	3	4
D199019	ewe	2021/9/26	32	48.5	76.9	69	79.2	7	21	2023/7/10	1	2
D188456	ewe	2019/6/9	60	47.7	75.7	68.5	79.1	6.9	48	2023/6/14	1	2
K2010010	ewe	2019/10/27	55	47.6	77.2	67.3	83.7	7.2	42	2023/5/16	3	4
F199418	ewe	2021/9/10	33	49.3	80.6	70.2	92.4	7.2	20	2023/5/11	2	2
D2110547	ewe	2018/2/26	75	40.2	70.5	66.5	82.4	6.8	60	2023/3/19	2	2
F217108	ewe	2019/8/25	58	44.2	72.8	66.5	82.3	7.3	44	2023/5/16	3	4
F217360	ewe	2020/9/22	45	44	79.5	66.8	80.4	7.4	31	2023/5/11	2	3
D188410	ewe	2019/11/12	55	48.1	75.2	68.5	81.7	7	43	2023/6/14	3	2
F217301	ewe	2021/9/8	33	60.5	83.8	78.5	79.1	9.2	20	2023/5/28	3	NA
E188386	ewe	2021/10/3	32	50.2	76.5	69.7	85.3	7	23	2023/9/3	3	2
E217369	ewe	2021/1/12	41	46.2	74.8	68.4	91.5	7.7	28	2023/5/14	3	2
C199307	ewe	2021/9/28	32	49.9	75.7	71.2	85.3	7.1	19	2023/5/18	3	3
C198355	ewe	2020/5/14	49	46.9	78.7	70.4	90.2	7	37	2023/6/14	2	2

E217317	ewe	2021/2/3	40	36.8	71.3	68.5	84.2	6.9	27	2023/5/5	3	3
I186077	ewe	2021/9/26	32	47.8	73.4	70.1	84.6	7.2	19	2023/5/14	3	4
E208260	ewe	2019/9/26	56	53.1	81.2	70.6	89.5	7.5	43	2023/5/5	3	2
E196341	ewe	2020/2/3	52	49.2	80.9	71	90.7	7.3	37	2023/3/3	3	2
C2110448	ewe	2018/2/19	76	40.5	76.8	70.6	82.8	8.2	60	2023/3/10	2	2
C2110515	ewe	2020/9/17	45	34.6	72.4	68.5	81.5	7.5	30	2023/3/26	2	2
E217024	ewe	2019/11/23	55	50.1	76.9	69.6	83.8	6.7	39	2023/3/16	3	2
C2110499	ewe	2019/11/16	55	39.5	71.2	68.5	81.3	7.8	39	2023/3/11	3	2
E2010154	ewe	2021/1/29	40	50.1	78.6	70.1	88.2	7.3	25	2023/3/3	3	2
I219037	ewe	2020/1/21	53	55	75.6	66	82.4	7	37	2023/2/27	4	2
C2110494	ewe	2019/8/25	58	40.1	75.3	68.3	86.2	7.9	42	2023/3/15	5	2
C196314	ewe	2018/1/23	77	46.2	79.2	68.6	88.3	7	61	2023/3/17	2	2
C2010077	ewe	2021/1/29	40	48.7	75.2	68.4	78.9	7	27	2023/5/4	4	2
C189121	ewe	2018/1/19	77	50	78.7	69.4	84.6	7.5	63	2023/5/15	3	2
I2010371	ewe	2021/10/6	32	48	73.4	67.2	77.6	6.8	19	2023/5/14	2	2
C199534	ewe	2021/10/8	32	47.3	76.8	68.5	82.7	7.5	19	2023/5/28	2	3
C199477	ewe	2020/1/8	53	56.4	84.9	75.7	99.2	7.6	41	2023/6/27	1	2
C2110026	ewe	2021/9/18	33	52.1	78.4	71.5	85.8	6.8	20	2023/6/12	2	4
I2010058	ewe	2021/10/3	32	54.5	76.7	74	90	7.5	20	2023/6/10	3	4
C196234	ewe	2018/1/31	76	50.8	81.6	72.6	95.1	7.5	60	2023/2/9	4	5
C189070	ewe	2018/10/23	68	52.9	80.1	70.5	90.3	8	NA	NA	NA	2
I219018	ewe	2019/12/10	54	60.2	81.2	68.6	93.3	7.5	38	2023/2/15	1	2
E188051	ewe	2020/4/4	50	49.8	77.1	71.2	88.6	7.3	NA	NA	NA	2
C2110527	ewe	2018/9/2	69	37.2	74.8	68.3	81.3	8.6	NA	NA	NA	2
C2110533	ewe	2021/10/6	32	29.6	70.5	69.3	79.2	7.2	NA	NA	NA	2
C2010028	ewe	2019/12/23	54	48.2	79.1	67.8	87	7.7	38	2023/3/17	2	2
I219368	ewe	2019/4/6	62	39.8	74.2	68.3	81.2	8.4	46	2023/3/2	3	2
E217394	ewe	2022/1/27	28	41.1	78.6	67.5	86.9	7.3	13	2023/3/21	1	2
B189089	ewe	2022/1/27	28	48.2	75.2	69.1	84.7	7.2	13	2023/3/22	2	3
A189304	ewe	2018/1/18	77	48.2	74.5	69.1	84.6	7	60	2023/1/19	1	2
B198480	ewe	2016/10/24	92	51.3	77.5	68.6	86.8	8.2	76	2023/3/3	1	4
A209088	ewe	2020/1/19	53	49.7	75.6	71.2	87.4	7.3	37	2023/3/5	3	4
M218451	ewe	2021/10/2	32	46.3	81.4	66.4	94.5	7.4	15	2023/1/13	2	2
M198511	ewe	2019/11/25	55	47.2	73.6	67.5	80.8	6.9	39	2023/3/14	4	2
M199133	ewe	2020/4/5	50	51.7	76.5	70.1	91.2	7.6	37	2023/5/17	3	4
G228137	ewe	2018/1/23	77	40.9	68.4	65.6	85.5	7.1	62	2023/4/18	1	3
G228134	ewe	2021/9/23	33	42.5	72.4	66.2	83.8	7.6	19	2023/5/16	2	2
B189044	ewe	2019/5/30	60	49.9	76.6	73	86.4	7.3	48	2023/6/3	2	4
M168163	ewe	2021/6/18	36	46.3	73	65	87	7.4	23	2023/6/3	3	2
M209044	ewe	2018/10/8	68	51.1	80.4	72.2	91.1	7.5	55	2023/5/18	3	2
M218450	ewe	2021/2/12	40	50.6	74.1	72.6	84.5	8.1	27	2023/5/31	2	2
M198431	ewe	2018/2/24	76	50.4	75.5	70.8	89.6	7	62	2023/5/15	3	2
A207025	ewe	2019/5/7	61	51.6	80.5	70.5	89	7.5	44	2023/1/17	3	3

B189068	ewe	2019/12/26	53	54.9	82.4	70.6	90.1	7.4	40	2023/5/6	1	4
M218398	ewe	2021/6/7	36	39.6	75.4	67.5	84.1	6.8	23	2023/5/14	2	3
M218339	ewe	2021/10/14	32	43.5	70	68	82	7	19	2023/6/2	3	2
G188326	ewe	2018/10/3	68	50.7	76.5	69	83.4	7.2	51	2023/1/26	2	2
G218060	ewe	2019/4/25	62	45	70.4	66	79.5	7	45	2023/1/26	1	2
B196202	ewe	2022/1/7	29	49.9	76.5	70.5	83.1	7	11	2023/1/4	2	2
A198513	ewe	2018/1/12	77	45.4	70.9	68.5	78.9	7	NA	NA	NA	2
G218308	ewe	2020/1/20	53	48	75	68	89	7	35	2023/1/6	2	2
G218469	ewe	2018/9/12	69	40.1	70.1	66.5	86.2	7.4	55	2023/5/3	2	2
B189288	ewe	2021/9/24	33	56.4	82.8	71.5	94	7.6	19	2023/5/15	2	4
G198181	ewe	2021/9/16	33	51.7	75.5	73.6	88.7	7	20	2023/6/4	1	2
I229452	ewe	2018/9/14	69	35.5	74.5	68.3	86.8	7.6	56	2023/6/6	2	2
M186008	ewe	2021/6/11	36	52.7	75.5	71	97.3	7.2	22	2023/5/9	2	2
B209052	ewe	2018/2/25	76	54.8	80.5	69.4	91	7.5	62	2023/5/17	3	4
A189257	ewe	2019/11/28	54	48.2	75.9	69.1	82.7	7.1	41	2023/4/30	3	2
M218404	ewe	2021/10/1	32	36.4	70.7	69.9	87.2	7.8	19	2023/5/9	2	2
A219358	ewe	2018/2/18	76	35.4	73.8	70.2	78.2	7.9	62	2023/5/8	2	2
A189269	ewe	2021/1/31	40	50	76.7	70.4	89	7.2	28	2023/6/7	4	2
G218321	ewe	2019/11/15	55	43.5	68	66	80	6.7	42	2023/6/9	4	2
M186074	ewe	2019/6/16	60	48.7	77.6	70.2	85.6	7.3	46	2023/5/8	3	2
G186059	ewe	2018/12/14	66	48.5	77.4	70.1	83.6	7.3	54	2023/6/24	2	2
B217054	ewe	2018/4/5	74	47.3	78.5	70	80.9	7	57	2023/1/8	3	2
A198381	ewe	2021/1/28	40	51.7	85.6	72.8	87.9	7.7	27	2023/5/17	2	2
A198257	ewe	2019/12/14	54	45.8	71.8	66.7	81.5	6.6	41	2023/6/12	3	2
A189390	ewe	2021/2/5	40	50.7	78.1	68.4	88.3	7	27	2023/5/14	2	2
B186126	ewe	2021/1/30	40	55.1	75.6	70	97.2	8	28	2023/5/31	2	3
B217045	ewe	2018/2/27	75	45.6	75	69	84.5	7	62	2023/5/17	3	2
A196435	ewe	2021/2/17	40	50.8	79.6	68.7	85.4	7.8	27	2023/5/19	2	2
M218022	ewe	2020/2/1	52	44.4	79.2	69.5	80.2	7	40	2023/6/7	4	2
M186079	ewe	2019/9/25	57	51.9	79.2	73	90.1	7.5	43	2023/5/19	3	2
B219070	ewe	2021/10/6	32	52.2	76.7	70	84	7.2	19	2023/5/20	2	2
M209058	ewe	2019/8/28	57	59.2	81.9	74	94.7	8	44	2023/5/12	2	4
A198339	ewe	2018/12/15	66	49.4	78.5	70.3	91.2	7.3	53	2023/6/1	2	2
M198277	ewe	2019/6/2	60	50.5	79.9	68.9	84.8	7.3	46	2023/4/27	1	2
A189392	ewe	2019/11/15	55	54	80.5	70.9	91.6	7.7	42	2023/5/16	3	2
A198245	ewe	2021/2/3	40	49.7	75.8	68.5	87.6	7.4	28	2023/6/9	3	2
M218039	ewe	2022/1/11	29	48	75	68.5	86	7	15	2023/4/22	2	2
G228040	ewe	2018/9/8	69	39.8	67.8	67.8	77.7	7.3	57	2023/6/8	3	2
G186055	ewe	2018/2/16	76	50.4	79	71.2	87.3	7.4	62	2023/5/14	3	4
G228072	ewe	2022/1/19	29	36.3	64.2	60.6	83.4	6.8	14	2023/4/4	2	2
A209086	ewe	2020/4/2	50	45.8	71.7	66.4	75.8	6.6	38	2023/6/7	2	4
A198335	ewe	2019/9/19	57	46.7	77.7	69.2	87.6	6.9	43	2023/5/7	2	2
G218333	ewe	2021/6/16	36	47.8	75	68	84	7	22	2023/5/12	2	2

A189004	ewe	2018/1/2	77	51.2	76.2	71.5	90.1	7.2	64	2023/5/10	3	2
I229356	ewe	2019/12/3	54	47.1	69.1	71.8	78.9	7.1	41	2023/5/17	3	2
